# Synthesis and Development of *N*-2,5-Dimethylphenylthioureido Acid Derivatives as Scaffolds for New Antimicrobial Candidates Targeting Multidrug-Resistant Gram-Positive Pathogens

**DOI:** 10.3390/antibiotics12020220

**Published:** 2023-01-20

**Authors:** Povilas Kavaliauskas, Birutė Grybaitė, Rita Vaickelionienė, Birutė Sapijanskaitė-Banevič, Kazimieras Anusevičius, Agnė Kriaučiūnaitė, Gabrielė Smailienė, Vidmantas Petraitis, Rūta Petraitienė, Ethan Naing, Andrew Garcia, Vytautas Mickevičius

**Affiliations:** 1 Department of Organic Chemistry, Kaunas University of Technology, Radvilėnų Rd. 19, LT-50254 Kaunas, Lithuania; 2 Transplantation-Oncology Infectious Diseases Program, Division of Infectious Diseases, Department of Medicine, Weill Cornell Medicine of Cornell University, 1300 York Ave., New York, NY 10065, USA; 3 Institute for Genome Sciences, School of Medicine, University of Maryland, 655 W. Baltimore Street, Baltimore, MD 21201, USA; 4 Institute of Infectious Diseases and Pathogenic Microbiology, Birštono Str. 38A, LT-59116 Prienai, Lithuania

**Keywords:** 2-aminothiazoles, antifungal, antibacterial, anticancer activity, hydrazone, benzimidazole, sulphanilamide

## Abstract

The growing antimicrobial resistance to last-line antimicrobials among Gram-positive pathogens remains a major healthcare emergency worldwide. Therefore, the search for new small molecules targeting multidrug-resistant pathogens remains of great importance. In this paper, we report the synthesis and in vitro antimicrobial activity characterisation of novel thiazole derivatives using representative Gram-negative and Gram-positive strains, including tedizolid/linezolid-resistant *S. aureus,* as well as emerging fungal pathogens. The 4-substituted thiazoles **3h**, and **3j** with naphthoquinone-fused thiazole derivative **7** with excellent activity against methicillin and tedizolid/linezolid-resistant *S. aureus*. Moreover, compounds **3h**, **3j** and **7** showed favourable activity against vancomycin-resistant *E. faecium*. Compounds **9f** and **14f** showed broad-spectrum antifungal activity against drug-resistant *Candida* strains, while ester **8f** showed good activity against *Candida auris* which was greater than fluconazole. Collectively, these data demonstrate that *N*-2,5-dimethylphenylthioureido acid derivatives could be further explored as novel scaffolds for the development of antimicrobial candidates targeting Gram-positive bacteria and drug-resistant pathogenic fungi.

## 1. Introduction

Infections caused by Gram-positive pathogens, such as methicillin-resistant *Staphylococcus aureus* (MRSA) and vancomycin-resistant enterococci (VRE) remain among one of the most common infectious agents worldwide. Infections caused by MRSA and VRE are responsible for increased mortality rates among hospitalised patients with chronic illness [1,2]. Numerous virulence factors are harboured by MRSA and VRE as well as biofilm production often leading to infections in surgically implanted catheters, especially in patients receiving cancer chemotherapy, hematopoietic stem-cell transplantation, and solid-organ transplantation. The profound antimicrobial resistance among MRSA and VRSA shortens the available treatment options, resulting in the systemic manifestation of the pathogen and death. Therefore, it is important to develop and investigate novel compounds with antimicrobial activity directed to MRSA and VRE.

The ability of pathogens to form biofilms on indwelling catheters has previously been associated with greater morbidity [3,4]. Moreover, the formation of interkingdom biofilms, consisting of bacterial pathogens and *Candida* species worsens the clinical prognosis. Despite being the most common fungal pathogen in clinical settings responsible for great morbidity worldwide, *Candida* spp. can form synergistic relationships with *S. aureus* leading to bacterial protection from antimicrobial treatment. Moreover, there is evidence that *S. aureus* provides a niche for *C. albicans* to evade antifungal drugs and thus survive the treatment [5,6]. Moreover, the recent emergence of the highly resistant *C. auris* with an instinctive resistance to azoles remains an increasing hardcore threat. Therefore, it is critical to apply novel concepts and develop multifunctional compounds with the ability to evade pre-existing antimicrobial activity in Gram-positive pathogens and clinically important fungi [7,8,9]. 

The development of new antimicrobials is focused on aspects of enhancing antimicrobial properties as well as evading antimicrobial resistance of bacterial and fungal pathogens or restoring the susceptibility of the pathogens to clinically approved antimicrobials [10,11,12,13,14,15]. In addition, compounds should remain minimally toxic to the host and show good pharmacological properties. However, the main aspect of developing effective drugs is their structural characteristics and the rate of activity. Lipophilicity is a significant physicochemical parameter that influences the membrane’s transport and the binding’s ability to act [16,17,18,19]. The study of lipophilicity-related parameters of thiazole derivatives showed these compounds to be promising drug candidates [20]. 

Thiazole derivatives are a family of heterocyclic compounds with large-scale biological properties [21] and are well-known in medicinal chemistry as promising drug candidates [22,23,24]. Synthesis of variously substituted thiazole ring led to novel compounds with numerous interesting pharmacological properties, including antibacterial [25,26,27,28,29], antifungal [15,20,23,30,31], antiviral [32,33,34,35], anthelmintic [36], antihypertensive, antihistaminic [37,38] and analgesic [39,40,41] effect. For instance, a naphthyl-substituted thiazole was established to inhibit the allosteric cysteine in the p10 subunit of caspase-5, thus acting as a cell’s protective compound [42]. Furthermore, benzimidazole-thiazole hybrid [43] was shown to be a privileged scaffold with a potent anti-inflammatory effect [40], and benzene sulphonamide thiazoles revealed its potent inhibitory properties against DPP-4 [27]. 

The 2,5-dimethylphenyl scaffold is a common structural feature in many antimicrobial compounds, particularly in the class of compounds known as phenylpropanoids [44]. These compounds have been found to have antimicrobial activity against a wide range of microorganisms, including bacteria, fungi and viruses [45,46,47]. They have been the subject of extensive research for the development of new antimicrobial agents, particularly in the fight against antibiotic-resistant infections [48]. Some examples of drugs that have been developed from the 2,5-dimethylphenyl scaffold include antifungal echinocandins and antibacterial agents such as linezolid. Therefore, novel compounds bearing 2,5-dimethylphenyl substituents may pose antimicrobial activity against Gram-positive and Gram-negative pathogens with novel or emerging resistance mechanisms. To explore aminothiazole derivatives as novel candidates targeting clinically important and multidrug-resistant WHO priority pathogens, we generated a series of aminothiazole derivatives bearing *N*-2,5-dimethylphenyl and β-alanine and characterised their antimicrobial activity using pathogens with defined resistance mechanisms. In addition to that, we characterised anticancer activity using A549 and Caco-2 cell culture models. A SAR investigation of aminothiazole derivatives revealed the influence of modification to a carboxylic acid moiety on the antimicrobial activity of the synthesized compounds [49]. In this paper, we describe the synthesis and in vitro characterisation of antimicrobial and anticancer properties of a series of novel thiazole derivatives bearing 4-quinolone, quinoxaline, naphthoquinone, hydrazone, benzimidazole, and benzenesulphonamide moieties. It is known that thiazoles can be synthesized from α-bromoketone and thiourea via *Hantzsch* thiazole synthesis in high yields.

## 2. Results and Discussion

### 2.1. Synthesis

**Scheme 1:**
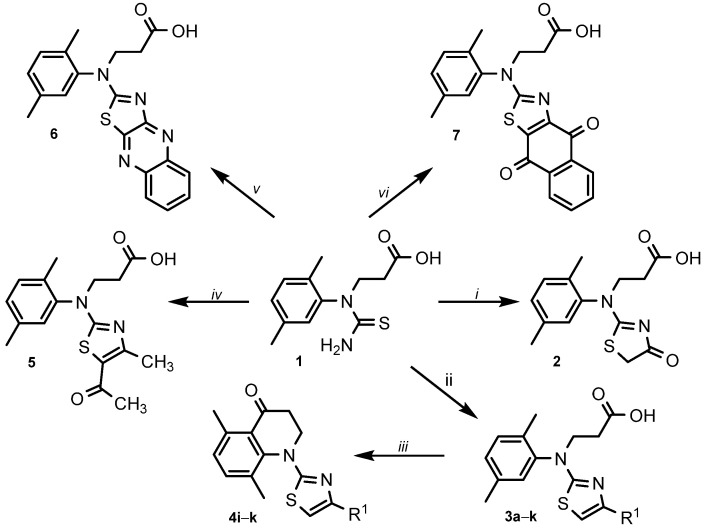
Synthesis of thiazole derivatives **2–7**. R^1^ = (**a**) H; (**b**) CH_3_; (**c**) C_6_H_5_; (**d**) 4-F-C_6_H_4_; (**e**) 4-CN-C_6_H_4_; (**f**) 4-Cl-C_6_H_4_; (**g**) 4-NO_2_-C_6_H_4_; (**h**) 3,4-diCl-C_6_H_4_; (**i**) 4-Br-C_6_H_4_; (**j**) naphthalen-2-yl; (**k**) chromenon-3-yl. Reagents and conditions: (i) ClCH_2_COOH, 10% K_2_CO_3_, r.t. 24 h, AcOH to pH 6; (ii) **a** 50% ClCH_2_CHO, acetone, reflux, 12 h; **b** ClCH_2_COCH_3_, water, rt, 24 h, AcONa, heated to boil; **c–k** *α*-bromoacetophenone, acetone, reflux, 2–5 h, (iii) PPA, 120 °C, 2–3 h, crushed ice; (iv) 3-chloropentane-2,4-dione, acetone, reflux, 2 h; (v) 2,3-dichloroquinoxaline or (vi) 2,3-dichloro-1,4-naphthoquinone, AcOH, AcONa, rt, 24 h, 70–80 °C, 10 h, water.

This research work is a continuation of projects on the synthesis and biological evaluation of various thiazole derivatives. 3-(1-(2,5-Dimethylphenyl)thioureido)propanoic acid (**1**) as the starting compound was synthesized by multistep reactions according to the method described in [50]. It is known that thiazoles can be synthesised from α-haloketone and a thioamide via *Hantzsch* thiazole synthesis in high yields [51]. To synthesize thiazolone **2** (Scheme 1), thioureido acid **1** was reacted with monochloroacetic acid in an aqueous 10% potassium carbonate solution at room temperature followed by the acidification with acetic acid to pH 6. In this reaction, the conventional reaction conditions at reflux were not suitable due to the formation of a large number of impurities. A singlet at 3.91 ppm of CH_2_ group protons in the ^1^H NMR spectrum and resonance lines at 183.3 ppm (C=O) and 187.0 ppm (C=N) in the ^13^C NMR spectrum of compound **2** confirmed the formation of the thiazolone ring. 2-Amino-1,3-thiazole derivative **3a** was obtained from thioureido acid **1** and a chloroacetaldehyde 50% aqueous solution. The reaction was performed by refluxing acetone for 12 h. Product **3a** was obtained in the form of water-soluble hydrochloride salt [52]. The convenience of the method is the crystallisation of the product from the reaction mixture already during the process. The data of the ^1^H and ^13^C NMR spectra of synthesized compounds **1**, **2 3a** are included in the Supplementary Material (Figures S1–S6).

Next, the 4-methyl substituted thiazole **3b** was synthesized as shown in Scheme 1. Previously, in [53], 4-methyl-2-aminothiazole was prepared in acetone, but for **3b**, the interaction of thioureido acid **1** with chloroacetone proceeded better in water than in acetone. The reaction at room temperature for 24 h and the following addition of sodium acetate to the reaction mixture gave the desired 4-methyl-1,3-thiazole derivative **3b**, the structure of which was easily confirmed by the NMR spectral data (Supplementary Material, Figures S7 and S8). 

According to the publication [54], 4-substituted thiazoles and especially those with chromen-3-yl and naphthalen-2-yl moieties demonstrate convincing antibacterial properties. Based on this, we have prepared a library of 4-substituted thiazoles **3c**–**k**. The well-known *Hantzsch* thiazole synthesis method was applied and the generation of the target products **3c**–**k** was performed by condensation of thioureido acid **1** with a series of *α*-bromoacetophenones without the use of a base. The isolated hydrobromide salts were transformed to free-based by dissolving them in 10% aqueous sodium carbonate and acidifying the solutions with acetic acid to pH 6. The structures of the obtained compounds **3c**–**k** were confirmed by the data of the ^1^H and ^13^C NMR spectra (Supplementary Material, Figures S9–S26).

The activity of broad-spectrum quinolone-based antibiotics against both Gram-positive and Gram-negative bacteria, including mycobacteria and anaerobes, promotes the synthesis and development of new quinolone-type compounds which are relevant to the development of knowledge on this topic [55]. Heating of *N*-aryl-*β*-alanines with strong dehydrating agents such as Eaton’s reagent [56], polyphosphoric acid [57,58] or phosphorus pentoxide [59] causes the formation of compounds containing 2,3-dihydroquinolin-4(1*H*)-one moiety. To obtain quinolone-type compounds **4i**–**k**, thiazoles **3i**–**k** were treated with polyphosphoric acid at 120 °C. The intramolecular cyclisation occurred over 2–3 h and 2,3-dihydroquinolin-4(1*H*)-ones **4i**–**k** were obtained in 71–88% yield. The data of the ^1^H and ^13^C NMR spectra of synthesized compounds **4i–k** are included in the Supplementary Material (Figures S27–S32). 

Compounds bearing a 5-acetyl-4-methylthiazole structure were found to exhibit high cytotoxicity against the MCF-7 cell line [60] as well as demonstrate up-and-coming antimicrobial properties [61,62]. For that purpose, we have included the synthesis of a compound containing this fragment into the goals of our study, and 5-acetyl-4-methylthiazole **5** was prepared by the same technique as for **3c**–**k**. The product was separated in 75% yield and its structure was confirmed by the methods of IR, NMR spectroscopy and the data of elemental analysis (NMR data are included in the Supplementary Material, Figures S33 and S34).

The previous study [63] on the synthesis and the assessment of biological properties of thiazole derivatives revealed naphthoquinone-fused derivatives to demonstrate good antimicrobial properties against Gram-positive and Gram-negative bacteria strains. The efforts to synthesize quinoxaline- and naphthoquinone-fused thiazoles **6** and **7** by the interaction of thioureido acid **1** with 2,3-dichloroquinoxaline or 2,3-dichloro-1,4-naphthoquinone, respectively, were successful. The stirring of the reaction mixture in glacial acetic acid with the presence of sodium acetate in the mixture at room temperature for 24 h and the additional stirring at the higher temperature of 70–80 °C for 10 h afforded thiazoles **6** and **7** (NMR data are included in the Supplementary Material, Figures S35–S38). An even higher temperature of the reaction mixture strongly reduces the yield of the products due to the formation 1-(2,5-dimethylphenyl)-2-thioxotetrahydropyrimidin-4(1*H*)-one caused by the intramolecular cyclisation of thioureido acid **1** [64]. Noteworthy, Matsuoka et al. state that in some cases the reactions of 2,3-dichloro-1,4-naphthoquinone with thioamides, thiourea and dithiooxamide led to the formation of dibenzo[*b,i*]thianthrene-5,7,12,14-tetraone as the main product of the reaction [65,66,67]. The same by-product was identified in the synthesis route of 3-[(2,5-dimethylphenyl)(4,9-dioxo-4,9-dihydronaphtho [2,3-*d*][1,3]thiazol-2-yl)amino]propanoic acid (**7**).

**Scheme 2:**
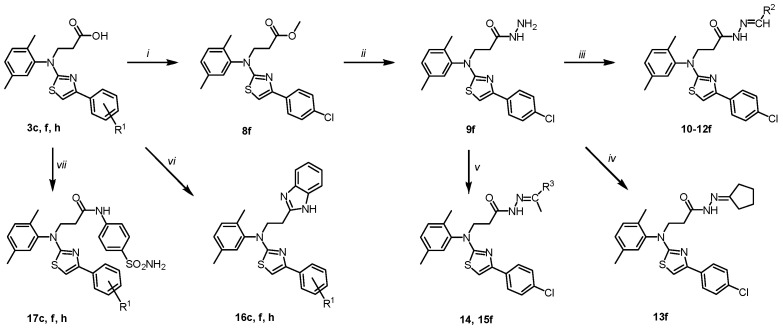
Chemical transformations of carboxylic acids **3c, f, h** and hydrazide **9f**. (**3**, **16**, **17c**) R^1^ = H; (**3**, **16**, **17f**) R^1^ = 4-Cl; (**3**, **16**, **17h**) R^1^ = 2,4-diCl; (**10f**) R^2^ = 5-nitrothiophen-2-yl; (**11f**) R^2^ = 5-nitrofuran-2-yl; (**12f**) R^2^ = indol-3-yl; (**14f**) R^3^ = Et; (**15f**) Hex. Reagents and conditions: (i) MeOH, a few drops of conc. H_2_SO_4_, reflux, 4 h; 5% Na_2_CO_3_ (ii) N_2_H_4_·H_2_O, 1,4-dioxane, reflux, 5 h; (iii) corresponding aldehyde, 1,4-dioxane, a few drops of glacial acetic acid, reflux, 2–12 h; (iv) cyclopentanone, 1,4-dioxane, a few drops of conc. acetic acid, reflux, 3 h; (v) corresponding ketone, 1,4-dioxane, a few drops of conc. acetic acid, reflux, 4 (**14**) or 8 (**15**) h; (vi) *o*-phenylenediamine, 15% HCl, reflux 72 h, water, 10% K_2_CO_3_; (vii) sulphanilamide, TEA, DMF, rt, 0.5 h, HBTU, DMF, argon, r.t. 72 h, 10% K_2_CO_3_.

The carboxyl functional group can be an important constituent of a pharmacophore [68]. Its functionalisation can greatly expand the library of biological properties of compounds [69,70]. To expand the library of thiazole derivatives and to evaluate the influence of substituents on their biological properties, some transformations of the carboxyl group were performed (Scheme 2). Firstly, acid **3f** was esterified with methanol to obtain ester **8f**. No additional catalyst was required for this reaction with hydrobromide analogue. Afterwards, ester **8f** was refluxed in 1,4-dioxane with hydrazine monohydrate and the resulting acid hydrazide **9f** was then condensed with aldehydes and ketones. The structure of **9f** was confirmed by the NMR techniques and microanalysis data. The presence of the CONHNH_2_ group protons were indicated by the singlets at 9.11 (NH) and 4.07 (NH_2_) ppm in the ^1^H NMR spectrum of **9f**, whereas the spectral line of the carbon of the C=O group resonated at the characteristic area of the ^13^C NMR spectrum, i.e., at 169.4 ppm (Supplementary Material, Figures S39 and S42).

As previously reported in [71], 4-aryl substituted thiazoles bearing hydrazonyl fragments appeared to be an eligible scaffold for the future development of antifungal and antibacterial agents targeting highly resistant pathogenic microorganisms. Further, a series of various hydrazones were synthesized and investigated for their bioactivity. To obtain hydrazones **10**–**15f**, hydrazide **9f** was condensed with heterocyclic and aliphatic ketones. The products were isolated in a 75–85% yield. The NMR spectra of the synthesized hydrazones **10**–**15f** showed that in the DMSO-*d*_6_ solution, they exist as a mixture of *E/Z* conformers due to the presence of a CO-NH fragment in the molecule and the restricted rotation around it; in the studies [57,72], the *Z*-form predominate are described NMR data are included in the Supplementary Material, Figures S43–S54). 

One of the methods for the synthesis of a benzimidazole heterosystem involves to condensation of carboxylic acids with 1,2-diaminobenzenes. The target compounds **16c, f, h** were synthesized by Phillip’s method (heating of both reagents in 4 M hydrochloric acid). The reflux for 72 h gave benzimidazoles **16c, f, h** in good yields (NMR data are in the Supplementary Material, Figures S45–S60). 

Finally, amides **17c**, **f**, **h** were prepared by direct coupling of acids **3** with the sulphanilamide using HBTU as the coupling reagent and triethylamine as the base (Scheme 2). 

The reactions were performed in dimethylformamide at room temperature in an inert argon atmosphere**.** The products were isolated by the dilution of the reaction mixture with an aqueous 10% potassium carbonate and were characterised using NMR and IR spectroscopy and elemental analysis (NMR data are included in the Supplementary Material, Figures S61–S66).

It is worth noting that the analysis of NMR spectra revealed that the CH_2_N protons are often observed as two broad singlets. The characteristic splitting of resonances was observed for all compounds except **4i**–**k**. The literature in [64] suggests that such splitting of the proton resonances of the compounds possessing methyl group in the 2nd position of the benzene ring was caused by the restricted rotation of benzene and other *N*-substituents around the C(1′)-N(1) bond and the possibility of diastereomers. 

### 2.2. Antibacterial Activity of Compounds ***1–17*** against Multidrug-Resistant Pathogens

In order to understand the structure-dependent antibacterial activity of novel thiazole derivatives **1**–**17,** we first used a representative collection of multidrug-resistant Gram-positive and Gram-negative bacterial pathogens with genetically defined antimicrobial resistance profiles [73]. The bacterial strains were selected to represent WHO ESKAPE group pathogens with emerging and challenging antimicrobial resistance mechanisms. To do so, the compounds were screened by using clinically approved minimal inhibitory concentration (MIC) determination by using the Clinical Laboratory Standard Institute (CLSI) recommendations.

Novel thiazole derivatives **1**–**17** exhibited structure-dependent antibacterial and antifungal activity. Interestingly, compounds **1**–**17** failed to show antimicrobial activity against multidrug-resistant Gram-negative pathogens, such as *K. pneumoniae, P. aeruginosa, A. baumannii* and *E. coli* (MIC > 64 µg/mL), suggesting the possible existence of Gram-positive bacteria-derived targets of compounds **1**–**17** (Table 1). On the other hand, only 4-substituted thiazoles **3h**, **3j** and ring-fused **7** showed favourable activity against Gram-positive bacteria (*S. aureus* and *E. faecium*) harbouring multidrug-resistance profiles (Table 2). Compound **3h** bearing 3,4-diCl-C_6_H_3_ moiety showed antimicrobial activity against *S. aureus* TCH 1516 (MIC 8 µg/mL) harbouring *mecA* gene conferring resistance to β-lactam antibiotics [1]. Moreover, **3h** showed one-fold lover antimicrobial activity against *E. faecium* AR-0783 (MIC 16 µg/mL) strain-harbouring *vanB* gene conferring resistance to vancomycin. The chlorines on the 3,4-diCl-C_6_H_4_ substituent could pose a synergistic activity by targeting multiple cellular targets on bacterial cells, in comparison to mono chlorine derivatives, thus exerting more potent antimicrobial activity. Moreover, dichloro substitutions could potentially greatly enhance lipophilicity, thus increasing compound penetration to bacterial cells. Finally, chlorine atoms are electron-windrowing groups and diCl substitution can greatly increase the electrophilicity of the compounds, thus greatly increasing reactivity and stronger interaction with microbial targets. Therefore, the chlorines at 3,4-positions of the phenyl ring are important for antimicrobial activity against Gram-positive multidrug-resistant pathogens [53]. The introduction of the 4-Cl-C_6_H_4_ substitution (**3f**) or the replacement of 3,4-diCl-C_6_H_4_ with 4-fluoro (**3d**) or 4-bromo (**3i**) phenyl substituents results in a complete loss of antimicrobial activity (MIC >64 µg/mL) against *S. aureus* and *E. faecium* suggesting the high importance of 3,4-diCl-C_6_H_4_ for the antimicrobial activity. Interestingly, the introduction of naphthalen-2-yl substitution (**3j**) greatly enhances the antimicrobial activity against *S. aureus* and *E. faecium* (MIC 2 µg/mL). 

The replacement of the thiazole ring with the ring-fused structure strongly enhanced the antibacterial activity against *S. aureus* TCH 1516 strain (MIC 1 µg/mL), while the antibacterial activity against *E. faecium* AR-0783 increased two-fold (MIC 8 µg/mL) if compared to **3h** (Table 1). The antibacterial activity of compound **7** against *S. aureus* was similar to daptomycin (MIC 1 µg/mL) and twice stronger than vancomycin (MIC 2 µg/mL) and excellent if compared to ampicillin (MIC > 64 µg/mL). 

The emerging resistance to oxazolidinones (linezolid and tedizolid) which are last-line antimicrobial pharmaceuticals used to treat infections caused by Staphylococcus remains one of the major healthcare threats worldwide. Therefore, we further evaluated if the most promising thiazole derivatives **3h, 3j** and **7** will be active against linezolid/tedizolid-resistant *S. aureus* strains (Table 2). The compounds **3h, 3j** and **7** showed promising activity against linezolid/tedizolid-resistant *S. aureus* strains with MIC ranging from 1 to 32 µg/mL (Table 2). Compound **3j** showed the highest activity against tested strains (MIC 1–2 µg/mL), and its antimicrobial activity was greater than linezolid (8–32 µg/mL). 

Collectively, these results demonstrated that substituted thiazoles exert in vitro antibacterial activity against Gram-positive bacterial pathogens harbouring emerging antibacterial resistance profiles. Moreover, 3,4-diCl-C_6_H_3_ and naphthalen-2-yl substitutions, as well as naphthothiazoledione moiety, are important for the potent in vitro antibacterial activity against *S. aureus* with challenging resistance mechanisms. 

### 2.3. Antifungal Activity of Thiazoles ***1–17*** against Drug-Resistant Candida Species

Antifungal drugs of the azole group are one of the most widely used for the treatment of invasive and systemic fungal infections [74]. Candida species are responsible for the majority of fungal infections, as rapidly rising resistance to azole antifungals among Candida species, in particular C. albicans is responsible for increased morbidity and mortality worldwide [75]. We, therefore, evaluated novel thiazoles **1–17** for their in vitro antifungal activity against multidrug-resistant Candida species by exposing the fungal strains with clinically relevant concentrations of each compound or control antifungal drug (Table 3). 

Interestingly, only thiazoles **8f, 9f** and **14f** showed in vitro antifungal activity against *Candida* strains, expressing the multidrug-resistance phenotype to azoles (Table 3). The transformation of thiazole **3f** (MIC > 64 µg/mL) to ester **8f** was observed to result in striking changes in antifungal activity. (Table 3). The derivative **8f** showed antifungal activity against all tested strains (1–8 µg/mL) including *Candida auris* AR-0383 strains to harbour instinctive resistance to clinically approved antifungal drugs. The ester **8f** transformation to hydrazide **9f** greatly affected the antifungal activity. The hydrazide **9f** was no longer active against *C. auris* (MIC > 64 µg/mL) and had decreased activity against *C. albicans* (MIC 8 µg/mL) in comparison to ester **8f** (MIC 1 µg/mL). Interestingly, despite the loss of activity against *C. auris,* hydrazide **9f** showed increased activity against *C. parapsilosis* AR-0335 (MIC 4 µg/mL). Numerous hydrazones were previously reported to show antifungal activity [76,77,78]; therefore, we further transformed hydrazide **9f** to the corresponding hydrazones **10–15f** and evaluated their structure-dependent antifungal activity in vitro. Hydrazone **14f** bearing the butan-2-ylidene substitution exhibited antifungal activity against the majority of tested fungi (MIC 8–32 µg/mL) with the exception of *C. auris* (MIC > 64 µg/mL). The substitution of this moiety with a longer octan-2-ylidene carbon chain (**15f**) results in a complete loss of antifungal activity against all tested strains. 

Thus, these results demonstrate novel thiazole derivatives **9f, 14f** and, especially, **8f** with a moderately functionalised carboxyl group exert the most promising antifungal activity against highly multidrug-resistant fungal pathogens. On the other hand, the functionalization of the carboxyl group is important for the broad-spectrum antifungal activity since bulky substitutions result in decreased antifungal activity spectrum or potency in these novel compounds. 

### 2.4. Anticancer Activity of Compounds ***1**–**17***

The thiazole scaffold of often explored by medicinal chemists as an attractive pharmacophore for the development of novel antineoplastic drug candidates [79,80,81]. Therefore, we used two well-described pulmonary (A549) [82] and corectal adenocarcinoma (Caco-2) [83] models to evaluate the anticancer activity of compounds **1–17.** We exposed the cells to 100 µM of each compound or cisplatin (CP) that was used as a clinically approved drug for the neoplastic disease treatment. The treatment was initiated for 24 h and after that, the cellular viability was measured by using MTT assay. 

**Figure 1:**
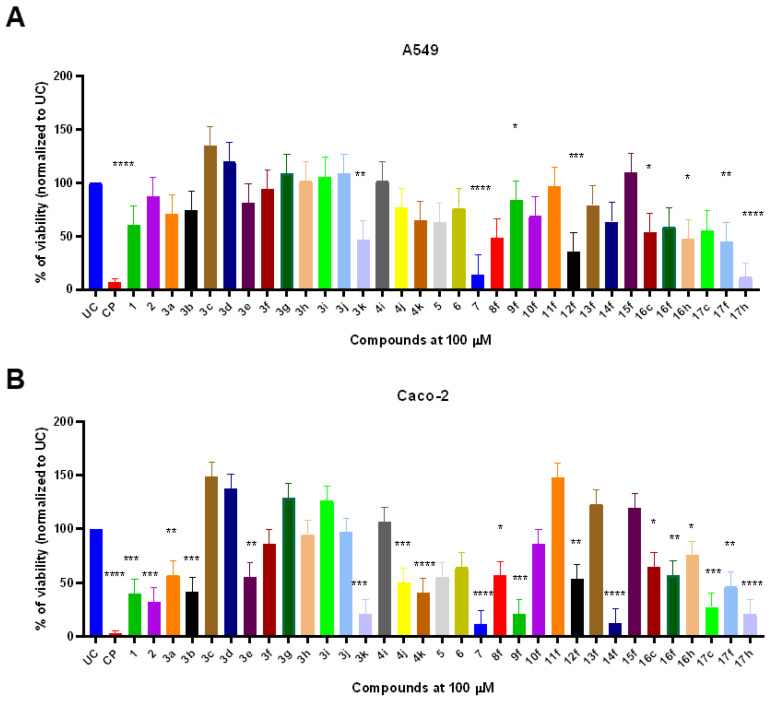
The in vitro anticancer activity of compounds **1–17** on A549 (**A**) and Caco-2 (**B**) adenocarcinoma cell line. Cells were exposed with fixed 100 µM of compounds **1–17** or cisplatin (CP) that served as a cytotoxicity control for 24 h. After the treatment, the remaining viability was determined by using MTT assay. Data represent the mean ± SD of triplicate experiments (*n* = 3). Statistical differences were determined using the Kruskal–Wallis test. * *p* < 0.05; ** *p* < 0.01, *** *p* < 0.001, **** *p* < 0.0001.

The novel thiazoles **1–17** demonstrated structure-dependent anticancer activity on A549 and Caco-2 cells. Interestingly, Caco-2 corectal adenocarcinoma cells showed significantly higher susceptibility to the treatment compounds in comparison to A549 cells (*p* < 0.05) suggesting the possible existence of selective novel thiazole-response involved targets in Caco-2. The starting compound **1** showed no significant anticancer activity against A549 human pulmonary adenocarcinoma cells. On the other hand, compound **1** significantly decreased the viability of Caco-2 cells (39.8%) in comparison to untreated control (UC) (*p* < 0.001) (Figure 1B). Thiazolone **2** resulted in slightly enhanced anticancer activity against Caco-2 (31.9%) while 2-amino-1,3-thiazole derivative **3a** showed decreased (56.9%) anticancer activity in comparison to UC (*p =* 0.0019). The addition of a 4-methyl group on the 1,3-thiazole ring (**3b**) enhanced the anticancer activity against Caco-2 cells (*p* = 0.004). Interestingly, compounds **2–3b** showed no anticancer activity against A549 cells (Figure 1A). 

Amongst 4-substituted thiazoles, only **3e** bearing 4-CN-C_6_H_4_ and **3k** with chromenon-3-yl substitutions showed anticancer activity against A549 and Caco-2 cells. Thiazole **3e** (R: 4-CN-C_6_H_4_) was able to significantly reduce the Caco-2 viability (54.9%) (*p =* 0.0011) while having no significant effect against A549 cells. Interestingly, the incorporation of chromenon-3-yl moiety (**3k**) significantly enhances the anticancer activity against Caco-2 (*p* = 0.001) as well as A549 cells (*p* = 0.0084) (Figure 1A,B). 

Quinolone-type compound **4i** bearing 4-Br-C_6_H_4_ substituent failed to significantly decrease the Caco-2 and A549 viability (106.1 and 101.4%, respectively). The incorporation of naphthalen-2-yl substitution (**4j**) significantly enhanced the anticancer activity against Caco-2 cells (*p* = 0.002) while chromenon-3-yl substitution (**4k**) resulted in significant 40.2% post-treatment viability of Caco-2 cells (*p <* 0.001) (Figure 1B).

5-Acetyl-4-methylthazole **5** did not significantly reduce the viability of A549 or Caco-2, although the compound was able to decrease the viability to 63 and 55%, respectively. Thiazole fusion with quinoxaline (compound **6**) did not result in significant viability reduction, although fusion with naphthoquinone lead to compound **7** with significantly high anticancer activity. Compound **7** exhibited broad-spectrum anticancer activity and was able to reduce A549 and Caco-2 viability to 14.0 and 13.1% compared to UC (*p* < 0.001) (Figure 1A,B). 

The carboxyl group transformation to ester **8f** in compound **3f** slightly enhanced the anticancer activity if compared to parent acid **3f** and showed similar anticancer efficacy against A549 and Caco-2 by reducing the viability to 48.1 and 56.4%, respectively. Interestingly subsequent ester **8f** transformation to hydrazide **9f** greatly enhanced the anticancer activity against Caco-2 cells (20.6%) while ameliorating compound-mediated cytotoxic activity against A549 cells (Figure 1A,B).

On the other hand, hydrazones **10–15f** showed highly structure-dependent activity. Hydrazones **10f** and **11f** bearing the 5-nitrothien-2-yl and 5-nitrofuran-2-yl substitutions showed no anticancer activity against both tested cancer cell lines. Compound **12f** containing indol-3-yl substitution demonstrated greater anticancer activity against A549 (35.0%) than Caco-2 (53.1%). The incorporation of cyclopentyl substitution resulted in compound **13f** with a complete loss of anticancer activity against both cell lines, while the incorporation of ethyl radical (**14f**) significantly enhanced the anticancer activity against Caco-2 cells, but not A549. On the other hand, the incorporation of the hexyl chain (**15f**) resulted in a complete loss of anticancer activity against Caco-2, suggesting the importance of ethyl radical in novel thiazole structures (Figure 1A,B). 

Benzimidazoles **16c,f,h** demonstrated similar anticancer activity in both A549 and Caco-2 cell lines by decreasing viability to approximately 47–60%. Amide **17c** containing 4-phenyl substituted thiazole showed potent anticancer activity against Caco-2 cells (27.2%) and little activity against A549 cells (55.4%). Compound **17f** bearing 4-Cl-C_6_H_4_ substituent at 4th position of the thiazole cycle showed similar activity in both cell lines (44.3 and 46.0%, respectively, while the incorporation of 2,4-diCl-C_6_H_3_ into the same position resulted in strong anticancer activity against both cell lines (Figure 1A,B). 

These results demonstrate that novel thiazole derivatives possess strong anticancer activity against different adenocarcinoma cell lines. The structure–activity relation studies showed that naphthoquinone-fusion and 2,4-diCl-C_6_H_3_ substitution are required for broad-spectrum anticancer activity. 

## 3. Materials and Methods

### 3.1. Synthesis

All reagents and used solvents were purchased from Sigma-Aldrich (St. Louis, MO, USA) and used as received without any purification. Reaction progress and compound purity were monitored by TLC using aluminium plates precoated with Silica gel with F254 nm (Merck KGaA, Darmstadt, Germany). Melting points were determined in an open capillary with a B-540 melting point apparatus (Büchi Corporation, New Castle, DE, USA) and were uncorrected. A Perkin–Elmer Spectrum BX FT–IR spectrometer (Perkin–Elmer Inc., Waltham, MA, USA) was used to record the IR spectra (ν, cm^−1^) and the pellets were prepared using KBr. The NMR spectra were recorded on Bruker Ascend 400 (^1^H 400 MHz, ^13^C 101 MHz) and Bruker Ascend (^1^H 700 MHz, ^13^C 176 MHz) spectrometers (Bruker BioSpin AG, Fällanden, Switzerland) using DMSO-*d_6_* and CDCl_3_ as solvents and TMS as an internal reference. The spectra data are presented as follows: chemical shift, multiplicity, integration, coupling constant [Hz] and allocation. The Elemental Analyzer CE-440 was used for elemental analyses (C, H, N) in good agreement (±0.3%) with the calculated values.

*3-[Carbamothioyl(2,5-dimethylphenyl)amino]propanoic acid* (**1**). The thioureido acid **1** was synthesized according to the method described in the literature [64]. Yield 85%, m.p. 134–135 °C (from water).

IR (KBr): ν 1167 (C=S), 1746 (C=O), 3342 (NH_2_), 3445 (OH) cm^−1^.

^1^H NMR (400 MHz, CDCl_3_): *δ* 2.13 (s, 3H, CH_3_), 2.29 (s, 3H, CH_3_), 2.49–2.56 (m, 2H, CH_2_CO), 3.76 (br. s,, 1H, NCH_2_), 4.44 (br. s, 1H, NCH_2_), 5.95 (br. s, 1H, NH_2_), 6.96 (s, 1H, H_Ar_), 7.10 (s, 1H, H_Ar_), 7.21 (s, 1H, H_Ar_), 7.50 (br. s, 1H, NH_2_), ppm.

^13^C NMR (101 MHz, CDCl_3_): *δ* 16.9 (CH_3_), 21.6 (CH_3_), 32.9 (CH_2_CO), 50.3 (NCH_2_), 128.4, 130.3, 132.1, 132.3, 138.1, 138.9 (C_Ar_), 176.9 (COOH), 181.6 (C=O) ppm.

Anal. Calcd for C_12_H_16_N_2_O_2_S: C, 57.12%; H, 6.39%; N, 11.10%; Found: C, 57.33%; H, 6.10%; N, 10.84%.

*3-[(2,5-Dimethylphenyl)(4-oxo-4,5-dihydro-1,3-thiazol-2-yl)amino]propanoic acid* (**2**). A mixture of thioureido acid **1** (1.26 g, 5 mmol), 40 mL of aqueous 10% potassium carbonate and chloroacetic acid (0.57 g, 6 mmol) was stirred at room temperature for 24 h. Then, the mixture was acidified with acetic acid to pH 6. Obtained the precipitate was filtered off, washed with water, and crystallized. Yield 0.88 g, 60%; m.p. 159–160 °C (from 2-propanol and water mixture 1:1).

IR (KBr): ν 1581 (C=N), 1674, 1725 (2C=O), 3290 (OH) cm^−1^.

^1^H NMR (700 MHz, DMSO-*d* _6_): *δ* 2.16 (s, 3H, CH_3_), 2.30 (s, 3H, CH_3_), 2.57 (t, 2H, *J* = 7.5 Hz, CH_2_CO), 3.72–3.81 (m, 1H, NCH_2_), 3.91 (s, 2H, SCH_2_), 4.24–4.39 (m, 1H, NCH_2_), 7.16 (s,1H, H_Ar_), 7.22 (d, *J* = 7.8 Hz, 1H, H_Ar_), 7.29 (d, *J* = 7.7 Hz, 1H, H_Ar_), 11.07 (br. s, 1H, OH) ppm.

^13^C NMR (176 MHz, DMSO-*d*_6_): *δ* 16.5 (CH_3_), 20.3 (CH_3_), 32.3 (CH_2_CO), 40.5 (SCH_2_), 49.5 (NCH_2_), 129.2, 130.6, 131.4, 132.7, 136.9, 138.7 (C_Ar_), 172.2 (COOH), 183.2 (C=O), 187.0 (C=N) ppm.

Anal. Calcd for C_14_H_16_N_2_O_3_S: C, 57.52%; H, 5.52%; N, 9.58%; Found: C, 57.27%; H, 4.28%; N, 9.39%.

*3-[(2,5-Dimethylphenyl)(1,3-thiazol-2-yl)amino]propanoic acid* (**3a**). A mixture of the thioureido acid **1** (0.5 g, 2 mmol), aqueous 50% chloroacetaldehyde solution (0.79 g, 10 mmol) and acetone (20 mL) was refluxed for 12 h. After that, the formed precipitate was filtered, washed with acetone and dried. Purification was performed by dissolving the crystals in aqueous 10% sodium carbonate (75 mL), filtering, and acidifying the filtrate with acetic acid to pH 6 (the procedure was repeated 2 times). Yield 0.35 g, 64%, m.p. 112–113 °C.

IR (KBr): ν 3076 (OH), 1717 (CO), 1514 (CN) cm^−1^. 

^1^H NMR (700 MHz, DMSO-*d* _6_): *δ* 2.10 (s, 3H, CH_3_), 2.29 (s, 3H, CH_3_), 2.62 (t, *J* = 7.3 Hz, 2H, CH_2_CO), 3.98 (br. s, 2H, NCH_2_), 6.66, 7.16 (2d, 2H, *J* = 3.6 Hz, SCH, NCH), 7.11 (s, 1H, H_Ar_), 7.13–7.16 (m, 1H, H_Ar_), 7.26 (d, 1H, *J* = 7.8 Hz, H_Ar_), 12.29 (s, 1H, OH) ppm.

^13^C NMR (176 MHz, DMSO-*d*_6_): *δ* 16.7 (CH_3_), 20.4 (CH_3_), 32.4 (CH_2_CO), 48.1 (NCH_2_), 108.0, 129.1, 129.2, 131.6, 133.0, 137.2, 139.3, 143.0 (C_Ar_ + SCH + NCH), 170.1 (C=N), 172.7 (COOH) ppm. 

Anal. Calcd for C_14_H_16_N_2_O_2_S: C, 60.85%; H, 5.48%; N, 10.14%; Found: C, 60.67%; H, 5.62%; N, 9.87%.

*3-[(2,5-Dimethylphenyl)(4-methyl-1,3-thiazol-2-yl)amino]propanoic acid* (**3b**). A mixture of thioureido acid **1** (1.26 g, 5 mmol), chloroacetone (0.64 g, 7 mmol) and water (20 mL) was stirred at room temperature for 24 h. Then, sodium acetate (0.82 g, 10 mmol) was added and the reaction mixture was brought to reflux. After cooling, the formed precipitate was filtered off and washed with water. Obtained organic salt was transformed to free-base by dissolving the crystals in 10% aqueous sodium carbonate (75 mL), filtering, and acidifying the filtrate with acetic acid to pH 6 (the purification procedure was repeated 2 times). Yield 0.88 g, 67%, m.p. 155–156 °C.

IR (KBr): ν 1527 (C=N), 1706 (C=O), 3392 (OH) cm^−1^. 

^1^H NMR (700 MHz, DMSO-*d* _6_): *δ* 2.09 (s, 3H, CH_3_), 2.15 (s, 3H, CH_3_), 2.28 (s, 3H, CH_3_), 2.60 (t, 2H, *J* = 7.3 Hz, CH_2_CO), 3.96 (br. s, 2H, NCH_2_), 6.20 (d, 1H, *J* = 1.1 Hz, SCH), 7.09 (d, 1H, *J* = 1.2 Hz, H_Ar_), 7.12 (dd, 1H, *J* = 7.9, 1.3 Hz, H_Ar_), 7.25 (d, 1H, *J* = 7.8 Hz, H_Ar_), 12.35 (s, 1H, OH) ppm.

^13^C NMR (176 MHz, DMSO-*d*_6_): *δ* 16.8 (CH_3_), 17.6 (CH_3_), 20.4 (CH_3_), 32.4 (CH_2_CO), 47.9 (NCH_2_), 102.1 (SCH), 129.2, 129.4, 131.6, 133.1, 137.1, 142.8, 148.5 (C_Ar_ + NC), 169.3 (C=N), 172.8 (COOH) ppm. 

Anal. Calcd for C_15_H_18_N_2_O_2_S: C, 62.04%; H, 6.25%; N, 9.65%; Found: C, 62.02%; H, 6.28%; N, 9.40%.

*General procedure for the synthesis of compounds***3c–k**. A mixture of the thioureido acid **1** (0.5 g, 2 mmol), the appropriate *α*-bromoacetophenone (2.2 mmol) and acetone (20 mL) was refluxed for 2–5 h and cooled down. The precipitate was filtered off, washed with acetone and dried. Obtained organic salts were transformed to free-base by dissolving the crystals in 10% aqueous sodium carbonate (75 mL), filtering and acidifying the filtrate with acetic acid to pH 6 (the purification procedure was repeated 2 times).

*3-[(2,5-Dimethylphenyl)(4-phenyl-1,3-thiazol-2-yl)amino]propanoic acid* (**3c**)

Yield 0.47 g, 67%, m.p. 104–105 °C. 

IR (KBr): ν 1537 (C=N), 1710 (C=O), 3390 (OH) cm^−1^. 

^1^H NMR (700 MHz, DMSO-*d*_6_): *δ* 2.14 (s, 3H, CH_3_), 2.29 (s, 3H, CH_3_), 2.63 (t, 2H, *J* = 7.3 Hz, CH_2_CO), 4.07 (br. s, 2H, NCH_2_), 7.08 (s, 1H, SCH), 7.11–7.17 (m, 2H, H_Ar_), 7.25–7.30 (m, 2H, H_Ar_), 7.39 (t, 2H, *J* = 7.7 Hz, H_Ar_) ppm. 

^13^C NMR (176 MHz, DMSO-*d*_6_): *δ* 16.8 (CH_3_), 20.4 (CH_3_), 33.1 (CH_2_CO), 48.5 (NCH_2_), 102.6 (SCH), 125.7, 127.4, 128.5, 129.2, 129.3, 131.5, 133.1, 134.8, 137.2, 142.7, 150.5 (C_Ar_ + NC), 169.3, (C=N), 173.1 (COOH) ppm.

Anal. Calcd for C_20_H_20_N_2_O_2_S: C, 68.16%; H, 5.72%; N, 7.95%; Found: C, 68.08%; H, 5.66%; N, 7.73%.

*3-{(2,5-Dimethylphenyl)[4-(4-fluorophenyl)-1,3-thiazol-2-yl]amino}propanoic acid* (**3d**)

Yield 0.56 g, 75%, m.p. 91–92 °C. 

IR (KBr): ν 1223 (C-F), 1540 (C=N), 1711 (C=O), 3384 (OH) cm^−1^. 

^1^H NMR (700 MHz, DMSO-*d*_6_): *δ* 2.14 (s, 3H, CH_3_), 2.30 (s, 3H, 2CH_3_), 2.68 (t, 2H, *J* = 7.2 Hz, CH_2_CO), 4.07 (br. s, 2H, NCH_2_), 7.07 (s, 1H, SCH), 7.12–7.17 (m, 2H, H_Ar_), 7.22 (t, 2H, *J* = 8.8 Hz, H_Ar_), 7.28 (d, 2H, *J* = 8.2 Hz, H_Ar_), 7.85–7.95 (m, 2H, H_Ar_) ppm. 

^13^C NMR (176 MHz, DMSO-*d*_6_): *δ* 16.8 (CH_3_), 20.4 (CH_3_), 32.6 (CH_2_CO), 48.2 (NCH_2_), 102.5 (SCH), 115.3, 115.4, 127.7, 129.3, 129.4, 131.3, 131.6, 133.0, 137.2, 142.7, 149.4, 160.9, 162.3 (C_Ar_ + NC), 169.4 (C=N), 172.8 (COOH) ppm. 

Anal. Calcd for C_20_H_19_FN_2_O_2_S: C, 64.85%; H, 5.17%; N, 7.56%; Found: C, 64.87%; H, 5.20%; N, 7.18%.

*3-{[4-(4-Cyanophenyl)-1,3-thiazol-2-yl](2,5-dimethylphenyl)amino}propanoic acid* (**3e**)

Yield 0.60 g, 80%, m.p. 132–133 °C. 

IR (KBr): ν 1536 (C=N), 1737 (C=O), 2223 (C≡N), 3261 (OH) cm^−1^. 

^1^H NMR (700 MHz, DMSO-*d*_6_): *δ* 2.15 (2s, 3H, CH_3_), 2.30 (2s, 3H, CH_3_), 2.65–2.75 (m, 2H, CH_2_CO), 3.86–4.28 (m, 2H, NCH_2_), 7.12–7.21 (m, 2H, H_Ar_ + SCH), 7.25–7.32 (m, 1H, H_Ar_), 7.41 (d, 1H, *J* = 8.0 Hz, H_Ar_), 7.85 (dd, 2H, *J* = 8.0, 4.9 Hz, H_Ar_), 8.05 (m, 2H, *J* = 8.3 Hz, H_Ar_) ppm. 

^13^C NMR (176 MHz, DMSO-*d*_6_): *δ* 16.6 (CH_3_), 20.4 (CH_3_), 32.2 (CH_2_CO), 47.4 (NCH_2_), 91.8 (SCH), 109.5, 119.0, 126.3, 129.5, 131.7, 132.61, 132.63, 133.0, 137.3, 138.8, 142.5, 148.7 (C_Ar_ + C≡N + NC), 169.6 (N=C), 172.7 (COOH) ppm. 

Anal. Calcd for C_21_H_19_N_3_O_2_S: C, 66.82%; H, 5.07%; N, 11.13%; Found: C, 66.63%; H, 5.04%; N, 11.06%.

*3-{[4-(4-Chlorophenyl)-1,3-thiazol-2-yl](2,5-dimethylphenyl)amino}propanoic acid* (**3f**)

Yield 0.64 g, 83%, m.p. 94–95 °C. 

IR (KBr): ν 835 (C-Cl), 1536 (C=N), 1710 (C=O), 3391 (OH) cm^−1^. 

^1^H NMR (700 MHz, DMSO-*d*_6_): *δ* 2.14, 2.16 (s, 3H, CH_3_), 2.30 (s, 3H, CH_3_), 2.57–2.75 (m, 2H, CH_2_CO), 4.10 (br. s, 2H, NCH_2_), 7.18 (d, 2H, *J* = 7.5 Hz, H_Ar_ + SCH), 7.30 (d, 1H, *J* = 7.6 Hz, H_Ar_), 7.47, 7.54 (2d, 2H, *J* = 8.4 Hz, H_Ar_), 7.80–8.01 (m, 2H, H_Ar_) ppm. 

^13^C NMR (176 MHz, DMSO-*d*_6_): *δ* 16.8 (CH_3_), 20.4 (CH_3_), 32.4 (CH_2_CO), 48.1 (NCH_2_), 103.7 (SCH), 127.4, 128.4, 128.6, 129.3, 129.4, 129.7, 131.7, 131.9, 133.0, 133.5, 137.3, 142.6, 149.1 (C_Ar_ + NC), 169.5 (C=N), 172.7 (COOH) ppm. 

Anal. Calcd for C_20_H_19_ClN_2_O_2_S: C, 62.09%; H, 4.95%; N, 7.24%; Found: C, 62.27%; H, 4.91%; N, 7.03%.

*3-{(2,5-Dimethylphenyl)[4-(4-nitrophenyl)-1,3-thiazol-2-yl]amino}propanoic acid* (**3g**)

Yield 0.63 g, 79%, m.p. 136–137 °C. 

IR (KBr): ν 1334, 1509 (NO_2_), 1540 (C=N), 1712 (C=O); 3430 (OH) cm^−1^. 

^1^H NMR (700 MHz, DMSO-*d*_6_): *δ* 2.13 (s, 3H, CH_3_), 2.28 (s, 3H, CH_3_), 2.46–2.61 (m, 2H, CH_2_CO), 3.85–4.22 (m, 2H, NCH_2_), 7.08–7.18 (m, 2H, H_Ar_ + SCH), 7.26 (d, 1H, *J* = 8.2 Hz, H_Ar_), 7.45 (s, 1H, H_Ar_), 8.10 (d, 2H, *J* = 8.8 Hz, H_Ar_), 8.24 (d, 2H, *J* = 8.9 Hz, H_Ar_) ppm. ^13^C NMR (176 MHz, DMSO-*d*_6_): *δ* 16.8 (CH_3_), 20.4 (CH_3_), 34.1 (CH_2_CO), 49.2 (NCH_2_), 107.3 (SCH), 124.0, 126.4, 129.3, 129.4, 131.6, 133.0, 137.2, 140.8, 142.5, 146.1, 148.5 (C_Ar_ + NC), 169.7 (C=N), 173.8 (COOH) ppm. 

Anal. Calcd for C_20_H_19_N_3_O_4_S: C, 60.44%; H, 4.82%; N, 10.57%; Found: C, 60.50%; H, 4.64%; N, 10.35%.

*3-{[4-(3,4-Dichlorophenyl)-1,3-thiazol-2-yl](2,5-dimethylphenyl)amino}propanoic acid* (**3h**)

Yield 0.71 g, 84%, m.p. 114–115 °C. 

IR (KBr): ν 1535 (C=N), 1710 (C=O), 3320 (OH) cm^−1^. 

^1^H NMR (400 MHz, DMSO-*d*_6_): *δ* 2.11, 2.27 (2s, 6H, 2CH_3_), 2.46–2.61 (m, 2H, CH_2_CO), 3.85–4.22 (m, 2H, NCH_2_), 7.04–7.19 (m, 2H, H_Ar_ + SCH), 7.20–7.38 (m, 2H, H_Ar_), 7.61 (d, 1H, *J* = 8.4 Hz, H_Ar_), 7.83 (dd, 1H, *J* = 8.2, 1.4 Hz, H_Ar_), 8.06 (s, 1H, H_Ar_) ppm. 

^13^C NMR (101 MHz, DMSO-*d*_6_): *δ* 16.8, 20.4 (2CH_3_), 33.8 (CH_2_CO), 48.9 (NCH_2_), 103.8 (SCH), 125.8, 127.1, 129.3, 129.4, 129.6, 130.8, 131.4, 131.6, 133.0, 135.3, 137.3, 142.5, 147.9 (C_Ar_, N–C), 169.5 (C=N), 171.7 (COOH) ppm. 

Anal. Calcd for C_20_H_18_Cl_2_N_2_O_2_S: C, 57.01%; H, 4.31% N, 6.65%; Found: C, 57.25%; H, 4.42%; N, 6.73%.

*3-{[4-(4-Bromophenyl)-1,3-thiazol-2-yl](2,5-dimethylphenyl)amino}propanoic acid* (**3i**)

Yield 0.64 g, 76%, m.p. 127–128 °C. 

IR (KBr): ν 1539 (C=N), 1710 (C=O), 3310 (OH) cm^−1^. 

^1^H NMR (400 MHz, DMSO-*d*_6_): *δ* 2.12 (s, 3H, CH_3_), 2.28 (s, 3H, CH_3_), 2.50–2.62 (m, 2H, CH_2_CO), 4.02 (br. s, 2H, NCH_2_), 7.06–7.30 (m, 4H, H_Ar_ + SCH), 7.56 (d, 2H, *J* = 8.5 Hz, H_Ar_), 7.81 (d, 2H, *J* = 8.6 Hz, H_Ar_) ppm. 

^13^C NMR (101 MHz, DMSO-*d*_6_): *δ* 16.8 (CH_3_), 20.4 (CH_3_), 33.7 (CH_2_CO), 49.0 (NCH_2_), 103.4 (SCH), 120.4, 127.7, 129.3, 129.4, 131.5, 131.6, 133.1, 134.0, 137.2, 142.7, 149.3 (C_Ar_, N–C), 169.5 (C=N); 173.7 (COOH) ppm. 

Anal. Calcd for C_20_H_19_BrN_2_O_2_S: C, 55.69%; H, 4.44%; N, 6.49%; Found: C, 55.93%; H, 4.46%; N, 6.28%.

*3-{(2,5-Dimethylphenyl)[4-(naphthalen-2-yl)thiazol-2-yl]amino}propanoic acid* (**3j**)

Yield 0.68 g, 84%, m.p. 121–122 °C. 

IR (KBr): ν 1536 (C=N), 1707 (C=O), 3363 (OH) cm^−1^. 

^1^H NMR (700 MHz, DMSO-*d*_6_): *δ* 2.16 (s, 3H, CH_3_), 2.29 (s, 3H, CH_3_), 2.48–2.54 (m, 2H, CH_2_CO), 4.09 (br. s, 2H, NCH_2_), 7.06–7.18 (m, 2H, H_Ar_ + SCH), 7.21 (s, 1H, H_Ar_), 7.27 (d, 1H, *J* = 7.7 Hz, H_Ar_), 7.36–7.61 (m, 2H, H_Ar_), 7.88, 7.95 (2d, 2H, *J* = 7.7 Hz, H_Ar_), 7.90 (d, 1H, *J* = 8.6 Hz, H_Ar_), 8.00 (d, 1H, *J* = 8.5 Hz, H_Ar_), ppm. 

^13^C NMR (176 MHz, DMSO-*d*_6_): *δ* 16.8 (CH_3_), 20.4 (CH_3_), 34.6 (CH_2_CO), 49.3 (NCH_2_), 103.1 (SCH), 124.1, 124.2, 125.8, 126.3, 127.5, 128.0, 128.1, 129.2, 129.4, 131.5, 132.3, 132.4, 133.1, 133.2, 137.1, 142.7, 150.5 (C_Ar_, N–C), 170.0 (C=N), 174.3 (COOH) ppm. 

Anal. Calcd for C_24_H_22_N_2_O_2_S: C, 71.62%; H, 5.51%; N, 6.96%; Found: C, 71.79%; H, 5.57%; N, 6.91%.

*3-{(2,5-Dimethylphenyl)[4-(2-oxo-2H-chromen-3-yl)thiazol-2-yl]amino}propanoic acid* (**3k**)

Yield 0.72 g, 86%, m.p. 164–165 °C. 

IR (KBr): ν 1540 (C=N), 1711, 1736 (2C=O), 3510 (OH) cm^−1^. 

^1^H NMR (400 MHz, DMSO-*d*_6_): *δ* 2.15 (s, 3H, CH_3_), 2.31 (s, 3H, CH_3_), 2.70 (t, 2H, *J* = 7.0 Hz, CH_2_CO), 3.95–4.35 (m, 2H, NCH_2_), 7.13–7.23 (m, 2H, H_Ar_ + SCH), 7.30 (d, 1H, *J* = 7.9 Hz, H_Ar_), 7.39 (t, 1H, *J* = 7.5 Hz, H_Ar_), 7.44 (d, 1H, *J* = 8.3 Hz, H_Ar_), 7.57 (s, 1H, H_Ar_), 7.62 (t, 1H, *J* = 7.8 Hz, H_Ar_), 7.90 (d, 1H, *J* = 7.7 Hz, H_Ar_), 8.67 (s, 1H, H_Ar_), 12.30 (s, 1H, OH) ppm. 

^13^C NMR (101 MHz, DMSO-*d*_6_): *δ* 16.7 (CH_3_), 20.4 (CH_3_), 32.4 (CH_2_CO), 47.7 (NCH_2_), 110.0, 115.9, 119.3, 120.5, 124.8, 128.9, 129.4, 129.6, 131.6, 131.7, 133.1, 137.3, 138.5, 142.4, 144.0, 152.3 (C_Ar_, N–C, SCH), 158.8 (O–C=O), 168.8 (C=N), 172.8 (COOH) ppm. 

Anal. Calcd for C_23_H_20_N_2_O_4_S: C, 65.70%; H, 4.79%; N, 6.66%; Found: C, 65.89%; H, 4.89%; N, 6.75%.

*General procedure for the synthesis of compounds***4i–k**. A mixture of the corresponding compound **3i–k** (2 mmol) and polyphosphoric acid (15 g) was stirred at 120 °C for 2–3 h; then, the reaction mixture was cooled down and crushed ice (150 g) was added. The precipitate was filtered off, washed with water and dried. Purification was performed by recrystallization from 2-propanol.

*1-[4-(4-Bromophenyl)-1,3-thiazol-2-yl]-5,8-dimethyl-2,3-dihydroquinolin-4(1H)-one* (**4i**)

Yield 0.73 g, 88%, m.p. 145–146 °C. 

IR (KBr): ν 1507 (C=N), 1673 (C=O) cm^−1^. 

^1^H NMR (400 MHz, DMSO-*d*_6_): *δ* 2.21 (s, 3H, CH_3_), 2.53 (s, 3H, CH_3_), 2.79 (t, 2H, *J* = 6.1 Hz, CH_2_CO), 4.28 (t, 2H, *J* = 6.0 Hz, NCH_2_), 7.18 (d, 1H, *J* = 7.9 Hz, H_Ar_), 7.42–7.49 (m, 2H, H_Ar_ + SCH), 7.57, 7.78 (2d, 4H, *J* = 8.5 Hz, H_Ar_) ppm. 

^13^C NMR (101 MHz, DMSO-*d*_6_): *δ* 18.1 (CH_3_), 22.4 (CH_3_), 39.3 (CH_2_CO), 49.8 (NCH_2_), 106.8, 121.3, 127.3, 128.2, 130.7, 131.9, 132.0, 133.9, 136.1, 139.1, 146.7, 149.9 (C_Ar_, SCN, N-C), 168.5 (C=N), 196.7 (C=O) ppm. 

Anal. Calcd for C_20_H_17_BrN_2_OS: C, 58.12%; H, 4.15%; N, 6.78%; Found: C, 58.32%; H, 4.23%; N, 6.80%.

*5,8-Dimethyl-1-[4-(naphthalen-2-yl)-1,3-thiazol-2-yl]-2,3-dihydroquinolin-4(1H)-one* (**4j**)

Yield 0.62 g, 81%, m.p. 141–142 °C. 

IR (KBr): ν 1547 (C=N), 1740 (C=O) cm^−1^. 

^1^H NMR (400 MHz, (CDCl_3_): *δ* 2.34 (s, 3H, CH_3_), 2.66 (s, 3H, CH_3_), 2.91 (t, 2H, *J* = 6.1 Hz, CH_2_CO), 4.48 (t, 2H, *J* = 6.0 Hz, NCH*_2_*), 6.96 (s, 1H, SCH), 7.12, 7.36 (2d, 2H, J = 7.8 Hz, H_Ar_), 7.39–7.56 (m, 2H, H_Ar_), 7.74–7.98 (m, 4H, H_Ar_), 8.38 (s, 1H, H_Ar_) ppm. 

^13^C NMR (101 MHz, (CDCl_3_): *δ* 18.2 (CH_3_), 22.9 (CH_3_), 39.4 (CH_2_CO), 49.9 (NCH_2_), 104.6 (SCH), 124.1, 125.2, 126.1, 126.4, 127.2, 127.8, 128.4, 128.5, 130.7, 132.0, 132.1, 133.2, 133.8, 136.1, 140.3, 147.0, 152.1 (C_Ar_, N–C), 169.0 (C=N), 196.9 (C=O) ppm. 

Anal. Calcd for C_24_H_20_N_2_OS: C, 74.97%; H, 5.24%; N, 7.29%; Found: C, 75.01%; H, 5.20%; N, 7.35%.

*5,8-Dimethyl-1-[4-(2-oxo-2H-1-benzopyran-3-yl)-1,3-thiazol-2-yl]-2,3-dihydroquinolin-4(1H)-one* (**4k**)

Yield 0.57 g, 71%, m.p. 205–206 °C.

IR (KBr): ν 1518 (C=N), 1681, 1716 (2C=O) cm^−1^. 

^1^H NMR (400 MHz, DMSO-*d*_6_): *δ* 2.23 (s, 3H, CH_3_), 2.54 (s, 3H, CH_3_), 2.81 (t, 2H, *J* = 6.2 Hz, CH_2_CO,), 4.37 (t, 2H, *J* = 6.1 Hz, NCH_2_), 7.21 (d, 1H, *J* = 7.9 Hz, H_Ar_), 7.34–7.54 (m, 3H, H_Ar_), 7.56–7.65 (m, 1H, H_Ar_), 7.79 (s, 1H, SCH), 7.86 (d, 1H, *J* = 6.9 Hz, H_Ar_), 8.63 (s, 1H, H_Chrom_) ppm. 

^13^C NMR (101 MHz, DMSO-*d*_6_): *δ* 17.5 (CH_3_), 22.0 (CH_3_), 38.9 (CH_2_CO), 49.2 (NCH_2_), 112.0, 115.9, 119.2, 120.2, 124.7, 126.9, 128.9, 130.5, 131.3, 131.8, 135.8, 138.8, 139.1, 144.1, 146.2, 152.4 (C_Ar_, SCN, N-C), 158.7 (O-C=O), 167.6 (C=N), 196.2 (CH_2_C=O) ppm. 

Anal. Calcd for C_23_H_18_N_2_O_3_S: C, 68.64%; H, 4.51%; N, 6.96%; Found: C, 68.49%; H, 4.53%; N, 6.99%.

*3-[(5-Acetyl-4-methyl-1,3-thiazol-2-yl)(2,5-dimethylphenyl)amino]propanoic acid* (**5**). A mixture of the thioureido acid **1** (0.5 g, 2 mmol), 3-chloropentane-2,4-dione (0.29 g, 2.2 mmol) and acetone (20 mL) was refluxed for 2 h and cooled down. The formed precipitate was filtered off, washed with acetone and dried. Purification was performed by dissolving the crystals in 10% aqueous sodium carbonate, filtering and acidifying the filtrate with acetic acid to pH 6 (the procedure was repeated 2 times).

Yield 0.50 g, 75%, m.p. 172–173 °C. 

IR (KBr): ν 1521 (C=N), 1656, 1717 (2C=O), 3631 (OH) cm^−1^. 

^1^H NMR (700 MHz, DMSO-*d*_6_): *δ* 2.11 (s, 3H, CH_3_), 2.29 (s, 3H, CH_3_), 2.30 (s, 3H, CH_3_), 2.50 (s, 3H, CH_3_), 2.57–2.65 (m, 2H, CH_2_CO), 3.79–3.93 (m, 1H, NCH_2_), 4.13–4.32 (m, 1H, NCH_2_), 7.13 (s, 1H, H_Ar_), 7.20 (d, 1H, *J* = 7.8 Hz, H_Ar_), 7.30 (d, 1H, *J* = 7.8 Hz, H_Ar_),12.36 (s, 1H, OH) ppm. 

^13^C NMR (176 MHz, DMSO-*d*_6_): *δ* 16.5 (CH_3_), 18.6 (CH_3_), 20.4 (CH_3_), 29.5 (CH_3_), 32.1 (CH_2_CO), 47.5 (NCH_2_), 122.8, 128.8, 130.0, 131.8, 132.5, 137.5, 141.5, 157.5 (C_Ar_ + SC + NC), 170.5 (C=N), 172.3 (COOH), 188.6 (CH_3_C=O) ppm. 

Anal. Calcd for C_17_H_20_N_2_O_3_S: C, 61.42%; H, 6.06%; N, 8.43%; Found: C, 61.19%; H, 6.20%; N, 8.53%.

*General procedure for the preparation of compounds* **6** *and* **7**. A mixture of thioureido acid **1** (1.26 g, 5 mmol), 2,3-dichloroquinoxaline (**6**) (1 g, 5 mmol) or 2,3-dichloro-1,4-naphthoquinone (1.18 g, 5 mmol), sodium acetate (1.64 g, 20 mmol) and glacial acetic acid (20 mL) was stirred at room temperature for 24 h. Then, the temperature was raised to 70–80 °C and the reaction was continued for another 10 h. After the completion of the rection, the mixture was diluted with water (30 mL), the precipitate was filtered off, washed with water and dried. Purification was performed by dissolving the crystals in 10% aqueous sodium carbonate, filtering and acidifying the filtrate with acetic acid to pH 6 (the procedure was repeated 2 times).

*3-[(2,5-Dimethylphenyl)([1,3]thiazolo [4,5-b]quinoxalin-2-yl)amino]propanoic acid* (**6**) 

Yield 1.30 g, 70%, m.p. 132–133 °C. 

IR (KBr): ν 1520, 1562, 1591 (3C=N), 1724 (C=O), 2922 (OH) cm^−1^. 

^1^H NMR (400 MHz, DMSO-*d*_6_): *δ* 2.20 (s, 3H, CH_3_), 2.33 (s, 3H, CH_3_), 2.50–2.61 (m, 2H, CH_2_CO), 3.91–4.00 (m, 1H, NCH_2_), 4.34–4.54 (m, 1H, NCH_2_), 7.06–7.37 (m, 3H, H_Ar_), 7.58, 7.67 (2t, 2H, *J* = 7.3 Hz, H_Ar_), 7.86 (t, 2H, *J* = 6.9 Hz, H_Ar_)ppm. 

^13^C NMR (101 MHz, DMSO-*d*_6_): *δ* 16.6, 20.4 (2CH_3_), 34.2 (CH_2_CO), 49.6 (NCH_2_), 126.9, 127.9, 129.0, 129.4, 130.7, 131.8, 132.8, 137.6, 137.9, 140.0, 140.6, 154.2, 158.7, 169.5 (C_Ar_ + 3C=N), 175.3 (COOH) ppm. 

Anal. Calcd for C_20_H_18_N_4_O_2_S: C, 63.47%; H, 4.79%; N, 14.80%; Found: C, 63.63%; H, 4.69%; N, 14.24%.

*3-[(2,5-Dimethylphenyl)(4,9-dioxo-4,9-dihydronaphtho [2,3-d][1,3]thiazol-2-yl)amino]propanoic acid* (**7**)

Yield 1.32 g, 65%, m.p. 194–195 °C. 

IR (KBr): ν 1530 (C=N), 1627, 1642, 1720 (3C=O), 3550 (OH) cm^−1^. 

^1^H NMR (400 MHz, CDCl_3_): *δ* 2.21 (s, 3H, CH_3_), 2.35 (s, 3H, CH_3_), 2.75–3.01 (m, 2H, CH_2_CO), 3.93–4.14 (m, 1H, NCH_2_), 4.30–4.92 (m, 1H, NCH_2_), 7.04, 7.06 (2s, 1H, H_Ar_), 7.20, 7.27 (2d, 2H, *J* = 7.8 Hz, H_Ar_), 7.42, 7.61 (2t, 2H, *J* = 7.4 Hz, H_Ar_), 7.99 (d, 1H, *J* = 7.5 Hz, H_Ar_), 8.04 (d, 1H, *J* = 6.3 Hz, H_Ar_) ppm.

^13^C NMR (101 MHz, CDCl_3_): *δ* 17.1, 21.0 (2CH_3_), 32.5 (CH_2_CO), 48.4 (NCH_2_), 122.30, 126.5, 127.4, 128.6, 129.7, 130.7, 130.8, 131.1, 132.3, 132.5, 132.6, 132.7, 133.5, 135.2, 138.5, 141.1, 162.0, 170.7 (C_Ar_), 176.2 (COOH), 177.3, 181.1 (2C=O) ppm. 

Anal. Calcd for C_22_H_18_N_2_O_4_S: C, 65.01%; H, 4.46%; N, 6.89%; Found: C, 64.88%; H, 4.57%; N, 6.74%.

*Methyl 3-{[4-(4-chlorophenyl)-1,3-thiazol-2-yl](2,5-dimethylphenyl)amino}propanoate* (**8f**). A mixture of acid **3f** (1.94 g, 5 mmol), methanol (50 mL) and H_2_SO_4_ (0.5 mL) was refluxed for 4 h. Then, the mixture was cooled down and the solvent was evaporated. Sodium bicarbonate solution (5%) was used to neutralize the residues, which was then extracted with diethyl ether (3 × 100 mL). Afterwards, the ether was evaporated to give the title compound **8f** (brown resin, 1.84 g, 92%), *R_f_* = 0.57 (ethyl acetate: hexane (1:10)). 

IR (KBr): ν 1174 (C-O), 1733 (C=O) cm^−1^.

^1^H NMR (400 MHz, DMSO-*d*_6_): *δ* 2.13 (s, 3H, CH_3_), 2.30 (s, 3H, CH_3_), 2.78 (t, *J* = 6.9 Hz, CH_2_CO), 3.53 (s, 3H, OCH_3_), 4.12 (br. s, 2H, NCH_2_), 7.07–7.22 (m, 3H, H_Ar_), 7.28 (d, 1H, *J* = 7.7 Hz, H_Ar_), 7.45 (d, 2H, *J* = 8.3 Hz, H_Ar_), 7.88 (d, 2H, *J* = 8.3 Hz, H_Ar_) ppm.

^13^C NMR (101 MHz, DMSO-*d*_6_): *δ* 16.7 (CH_3_), 20.4 (CH_3_), 32.3 (CH_2_CO), 48.0 (NCH_2_), 51.4 (OCH_3_), 103.7 (SCH), 127.3, 128.5, 129.2, 129.5, 131.6, 131.9, 133.0, 133.5, 137.2, 142.4, 149.2 (C_Ar_),169.4 (C=N), 171.6 (C=O) ppm. 

Anal. Calcd for C_21_H_21_ClN_2_O_2_S: C, 62.91%; H, 5.28%; N, 6.99%. Found: C, 62.99%; H, 5.14%; N, 7.07%.

*3-{[4-(4-Chlorophenyl)-1,3-thiazol-2-yl](2,5-dimethylphenyl)amino}propanehydrazide* (**9f**). A mixture of ester **8f** (1.20 g, 1 mmol), hydrazine monohydrate (0,45 g, 9 mmol) and 1,4-dioxane (20 mL) was refluxed in for 5 h. Then, the reaction mixture was cooled down, the formed precipitate was filtered off, washed with 2-propanol and dried. Purification was performed by recrystallization from 2-propanol.

Yield 1.02 g, 85%, m.p. 136–137 °C. 

IR (KBr): ν 1538 (C=N), 1662 (C=O), 3046 (NH), 3248 (NH_2_) cm^−1^.

^1^H NMR (400 MHz, DMSO-*d*_6_): *δ* 2.13 (s, 3H, CH_3_), 2.30 (s, 3H, CH_3_), 2.51–2.55 (m, 2H, CH_2_CO), 4.07 (br. s, 2H, NH_2_), 4.24 (br. s, 2H, NCH_2_), 7.08–7.24 (m, 3H, H_Ar_), 7.28 (d, 1H, *J* = 7.7 Hz, H_Ar_), 7.45 (d, 2H, *J* = 8.6 Hz, H_Ar_), 7.90 (d, 2H, *J* = 8.5 Hz, H_Ar_), 9.11 (s, 1H, NH) ppm. 

^13^C NMR (101 MHz, DMSO-*d*_6_): *δ* 16.8 (CH_3_), 20.4 (CH_3_), 31.9 (CH_2_CO), 48.7 (NCH_2_), 103.5 (SCH), 127.4, 128.5, 129.3, 129.4, 131.6, 131.8, 133.0, 133.6, 137.2, 142.7, 149.3 (C_Ar_, NC), 169.35 (C=N), 169.4 (C=O) ppm. 

Anal. Calcd for C_20_H_21_ClN_4_OS: C, 59.92%, H, 5.28%; N, 13.97%; Found: C, 59.75%; H, 5.36%; N, 13.91%.

*General procedure for the synthesis of hydrazones***10–12f**. A mixture of compound **8f** (0.40 g, 1 mmol), corresponding carbaldehyde (1.1 mmol), glacial acetic acid (2 drops) and 1,4-dioxane (20 mL) was heated at refluxed for 2–12 h. Then, the reaction mixture was cooled down, the formed precipitate was filtered off, washed with 2-propanol and dried. Purification was performed by recrystallization from 2-propanol. 

(*Z/E*)-3-{[4-(4-Chlorophenyl)thiazol-2-yl](2,5-dimethylphenyl)amino}-*N*’-[(5-nitrothiophen-2-yl)methylene]propanehydrazide (**10f**)

Yield 0.43 g, 79%, m.p. 102–103 °C. 

IR (KBr): ν 1534 (C=N), 1679 (C=O), 3121 (NH) cm^−1^.

^1^H NMR (400 MHz, DMSO-*d*_6_): *δ* 2.15, 2.16 (2s, 3H, CH_3_), 2.28, 2.29 (2s, 3H, CH_3_), 2.75 (t, 0.8H, *J* = 7.0 Hz, CH_2_CO), 3.08 (t, 1.2H, *J* = 7.0 Hz, CH_2_CO), 4.18 (br. s, 2H, NCH_2_), 6.96–7.35 (m, 6H, H_Ar_), 7.41, 7.44 (2d, 2H, *J* = 8.6 Hz, H_Ar_), 7.74, 7.77 (2d, 1H, *J* = 3.9 Hz, H_Ar_), 7.86–7.90 (m, 1H, H_Ar_), 7.91 (s, 0.6H, N=CH), 8.12 (s, 0.4H, N=CH), 11.78 (s, 0.6H, NH), 11.88 (s, 0.4H, NH) ppm.

^13^C NMR (101 MHz, DMSO-*d*_6_): *δ* 16.8 (CH_3_), 20.4 (CH_3_), 20.5 (CH_3_), 30.5 (CH_2_CO), 33.1 (CH_2_CO), 47.7 (NCH_2_), 48.2 (NCH_2_), 103.5 (SCH), 103.6 (SCH), 114.4, 114.6, 115.1, 127.3, 127.4, 128.5, 129.3, 129.4, 130.9, 131.6, 131.8, 133.0, 133.1, 133.5, 133.6, 134.0, 137.2, 142., 142.7, 149.2, 149.3, 151.6, 151.7 (C_Ar_, N-C_thiaz_), 167.4 (N-C=N), 169,4 (C=N), 169,5 (C=N), 172,9 (C=O) ppm. 

Anal. Calcd for C_20_H_21_ClN_4_OS: C, 55.60%; H, 4.11%; N, 12.97%; Found: C, 55.63%; H, 3.97%; N, 13.06%.

*(Z/E)-3-((4-(4-Chlorophenyl)thiazol-2-yl)(2,5-dimethylphenyl)amino)-N’-((5-nitrofuran-2-yl)methylene)propanehydrazide* (**11f**)

Yield 0.45 g, 85%, m.p. 78–79 °C. 

IR (KBr): ν 1535 (C=N), 1679 (C=O), 3108 (NH) cm^−1^. 

^1^H NMR (400 MHz, DMSO-*d*_6_): *δ* 2.15, 2.16 (2s, 3H, CH_3_), 2.28, 2.30 (2s, 3H, CH_3_), 2.73 (t, 0.8H, *J* = 7.0 Hz, CH_2_CO), 3.08 (t, 1.2H, *J* = 7.0 Hz, CH_2_CO), 4.15 (br. s, 2H, NCH_2_), 7.07–7.23 (m, 3H, H_Ar_ + SCH), 7.28 (d, 1H, *J* = 7.7 Hz, H_Ar_), 7.32–7.44 (m, 2H, H_Ar_), 7.46, 7.50 (2d, 1H, *J* = 4.2 Hz, H_Ar_), 8.12, 8.41 (2s, 1H, N=CH), 11.79 (s, 0.6H, NH), 11.83 (s, 0.4H, NH) ppm. 

^13^C NMR (101 MHz, DMSO-*d*_6_): *δ* 16.7 (CH_3_), 16.8 (CH_3_), 20.4 (CH_3_), 30.7 (CH_2_CO), 33.1 (CH_2_CO), 47.9 (NCH_2_), 48.3 (NCH_2_), 103.5 (SCH), 103.6 (SCH), 127.3, 127.4, 128.4, 128.5, 128.8, 129.4, 129.5, 130.4, 130.5, 131.7, 131.8, 132.9, 133.0, 133.5, 133.6, 136.1, 137.2, 139.7, 142.4, 142.6, 146.8, 146.9, 149.3, 150.3, 150.64 (C_Ar_, N-C_thiaz_), 167.3 (N-C=N), 169.4 (C=N), 169.5 (C=N), 172.7 (C=O) ppm. 

Anal. Calcd for C_25_H_22_ClN_5_O_4_S: C, 57.31%; H, 4.23%; N, 13.37%; Found: C, 57.11%; H, 4.15%; N, 13.33%.

*(Z/E)-N′-((1H-Indol-3-yl)methylene)-3-((4-(4-chlorophenyl)thiazol-2-yl)(2,5-dimethylphenyl)amino)propanehydrazide* (**12f**)

Yield 0.40 g, 75%, m.p. 72–73 °C. 

IR (KBr): ν 1534, 1612 (C=N), 1660 (C=O), 3108, 3178 (2NH) cm^−1^. 

^1^H NMR (400 MHz, DMSO-*d*_6_): *δ* 2.16, 2.21, (2s, 3H, CH_3_), 2.27 (s, 3H, CH_3_), 2.69 (t, 0.8H, *J* = 7.2 Hz, CH_2_CO), 3.13 (s, 1.2H, CH_2_CO), 4.28 (br. s, 2H, NCH_2_), 6.92 (t, 1H, *J* = 7.5 Hz, H_Ar_), 7.10–7.45 (m, 8H, H_Ar_), 7.74, 7.76 (2d, 1H, *J* = 2.5 Hz, H_Ar_), 7.89, 7.92 (2d, 2.7H, *J* = 8.5 Hz, H_Ar_), 8.16, 8.32 (2s, 1H, N=CH), 8.21 (d, 0.3H, *J* = 7.8 Hz, H_Ar_), 11.06 (s, 0.6H, NH_ind_), 11.13 (s, 0.4H, NH_ind_), 11.50 (s, 0.6H, NH), 11.53 (s, 0.4H, NH) ppm. 

^13^C NMR (101 MHz, DMSO-*d*_6_): *δ* 16.80 (CH_3_), 16.83 (CH_3_), 20.4 (CH_3_), 20.5 (CH_3_), 30.6 (CH_2_CO), 33.0 (CH_2_CO), 47.9 (NCH_2_), 48.7 (NCH_2_), 103.4 (SCH), 103.5 (SCH), 111.5, 111.6, 111.7, 111.8, 120.3, 121.3, 121.9, 122.5, 122.6, 124.0, 124.3, 127.3, 127.4, 128.5, 128.6, 129.3. 129.4, 129.5, 130.0, 130.2, 131.6, 131.8, 131.9, 133.0, 133.1, 133.6, 133.7, 137.0, 137.1, 137.2, 140.3, 142.6, 142.7, 143.3, 149.3 (C_Ar_, N-C_thiaz_), 165.7 (N-C=N), 169.5 (C=N), 169.6 (C=N), 171.5 (C=O) ppm. 

Anal. Calcd for C_29_H_26_ClN_5_OS: C, 65.96%; H, 4.96%; N, 13.26%; Found: C, 66.17%; H, 4.91%; N, 13.02%.

*3-{[4-(4-Chlorophenyl)thiazol-2-yl](2,5-dimethylphenyl)amino}-N’-cyclopentylidenepropanehydrazide* (**13f**) 

A mixture of compound 8f (0.40 g, 1 mmol), cyclopentanone (0.092 g, 1.1 mmol), a few drops of glacial acetic acid and 1,4-dioxane (20 mL) was refluxed for 3 h. Then, the reaction mixture was cooled, and the precipitate was filtered, washed with 2-propanol and dried. Purification was performed by recrystallization from 1,4-dioxane.

Yield 0.34 g (73 %), m.p. 117–118 °C; 

IR (KBr): ν (cm^−1^) 1540 (C=N), 1662 (C=O), 3187 (NH) cm^−1^; 

^1^H NMR (400 MHz, DMSO-*d*_6_): *δ* 1.55–1.79 (m, 4H, CH_2_), 2.14 (s, 3H, CH_3_), 2.17–2.26 (m, 4H, CH_2_), 2.29 (s, 3H, CH_3_), 2.68 (t, 0.9H, *J* = 7.3 Hz, CH_2_CO), 2.97 (t, 1.1H, *J* = 7.3 Hz, CH_2_CO), 4.13 (br. s, 2H, NCH_2_), 7.13–7.18 (m, 3H, H_Ar_ + SCH), 7.25–7.30 (m, 1H, H_Ar_), 7.41–7.46 (m, 2H, H_Ar_), 7.88, 7.90 (2d, 2H, *J* = 6.0 Hz, H_Ar_), 9.93 (s, 0.45H, NH), 9.97 (s, 0.55H, NH); 

^13^C NMR (101 MHz, DMSO-*d*_6_): *δ* 16.8 (CH_3_), 20.4 (CH_3_), 24.2, 24.3, 24.4, 24.5, 28.0, 28.3, 32.3,32.8 (4CH_2_), 30.8 (CH_2_CO), 32.9 (CH_2_CO), 48.1 (NCH_2_), 48.5 (NCH_2_), 103.38 (SCH), 103.43 (SCH), 127.3, 127.4, 128.5, 129.29, 129.33, 131.6 131.8, 132.96, 133.02, 133.58, 133.62, 137.09, 137.14, 142.6, 142.7, 149.21, 149.24, (C_Ar_, N-C_thiaz_), 162.2 (N-C=N), 165.7, 166.4 (N-C=N), 169.38 (C=N), 169.43 (C=N), 172.3 (C=O); 

Anal. Calcd for C_25_H_27_ClN_4_OS: C, 65.96%; H, 4.96%; N, 13.26%; Found: C, 66.17%; H, 4.91%; N, 13.02%.

*General procedure for the synthesis of compounds***14f***and***15f.** A mixture of compound **8f** (0.40 g, 1 mmol), the corresponding ketone (1.1 mmol), 1,4-dioxane (20 mL) and glacial acetic acid (2 drops) was refluxed for 4–8 h. Then, the reaction mixture was cooled down, the obtained precipitate was filtered off, washed with 2-propanol and dried. Purification was performed by recrystallization from 1,4-dioxane. 

*(Z/E)-N′-(Butan-2-ylidene)-3-{[4-(4-chlorophenyl)thiazol-2-yl](2,5-dimethylphenyl)amino}propanehydrazide* (**14f**)

Yield 0.35 g, 77%, m.p. 86–87 °C. 

IR (KBr): ν 1514, 1537 (2C=N), 1669 (C=O), 3187 (NH) cm^−1^. 

^1^H NMR (400 MHz, DMSO-*d*_6_): *δ* 1.69 (m, 5H, CH_3_ and CH_2_), 2.14 (s, 3H, CH_3_), 2.29 (s, 3H, CH_3_), 2.50 (s, 3H, CH_3_), 2.68 (t, 1H, *J* = 7.3 Hz, CH_2_CO), 2.98 (t, 1H, *J* = 7.3 Hz, CH_2_CO), 4.12 (br. s, 2H, NCH_2_), 7.13–7.18 (m, 3H, H_Ar_ + SCH), 7.28 (d, 1H, *J* = 7.6 Hz, H_Ar_), 7.44 (d, 2H, *J* = 8.3 Hz, H_Ar_), 7.89 (dd, 2H, *J* = 8.0, 6.1 Hz, H_Ar_), 10.03 (s, 0.5H, NH), 10.08 (s, 0.5H, NH) ppm. 

^13^C NMR (101 MHz, DMSO-*d*_6_): *δ* 16.8 (CH_3_), 17.0 (CH_3_), 17.5 (CH_3_), 20.4 (CH_3_), 24.9 (CH_2_), 25.1 (CH_2_), 30.9 (CH_2_CO), 32.4 (CH_2_CO), 48.1 (NCH_2_), 48.5 (NCH_2_), 103.4 (SCH), 103.5 (SCH), 127.3, 127.4, 128.5, 129.3, 131.6, 131.8, 132.9, 133.0, 133.58, 133.62, 137.1, 137.2, 142.6, 142.7 149.2, 150.1, 154.7, (C_Ar_, N-C_thiaz_), 166.5 (N-C=N), 169.38 (C=N), 169.44 (C=N), 172.4 (C=O) ppm. 

Anal. Calcd for C_24_H_27_ClN_4_OS: C, 63.35%; H, 5.98%; N, 12.31%; Found: C, 63.41%; 5.92%; N, 12.09%.

*(Z/E)-3-{[4-(4-Chlorophenyl)thiazol-2-yl](2,5-dimethylphenyl)amino}-N’-(octan-2-ylidene)propanehydrazide* (**15f**)

Yield 0.38 g, 75%, m.p. 70–71 °C. 

IR (KBr): ν 1538 (C=N), 1669 (C=O), 3178 (NH) cm^−1^. 

^1^H NMR (400 MHz, DMSO-*d*_6_): *δ* 0.72–0.83 (m, 3H, CH_3_), 1.10–1.45 (m, 8H, 4CH_2_), 1.78, 1.81, 1.87 (3s, 3H, CH_3_), 2.01–2.25 (m, 5H, CH_2_ and CH_3_), 2.29 (2s, 3H, CH_3_), 2.69 (t, 0.8H, *J* = 7.3 Hz, CH_2_CO), 3.00 (t, 1.2H, *J* = 7.3 Hz, CH_2_CO), 4.12 (br. s, 2H, NCH_2_), 7.01–7.22 (m, 3H, H_Ar_ + SCH), 7.23–7.31 (m, 1H, H_Ar_), 7.36–7.51 (m, 2H, H_Ar_), 7.89 (t, 2H, J = 8.0 Hz, H_Ar_), 10.00 (s, 0.3H, NH), 10.08 (s, 0.1H, NH), 10.10 (s, 0.5H, NH), 10.22 (s, 0.1H, NH) ppm. 

^13^C NMR (101 MHz, DMSO-*d*_6_): *δ* 13.88 (CH_3_), 13.92 (CH_3_), 15.9 (CH_3_), 16.0 (CH_3_), 16.75 (CH_3_), 16.79 (CH_3_), 20.4 (CH_3_), 20.4 (CH_3_), 22.0 (CH_2_), 25.0 (CH_2_), 25.3 (CH_2_), 26.0 (CH_2_), 28.2 (CH_2_), 28.4 (CH_2_), 28.7 (CH_2_), 30.0 (CH_2_CO), 31.05 (CH_2_CO), 31.09 (CH_2_CO), 32.5 (CH_2_CO), 38.3 (CH_2_CO), 48.2 (NCH_2_), 48.5 (NCH_2_), 103.3 (SCH), 103.4 (SCH), 127.3, 127.4, 128.5, 129.27, 129.31, 131.5, 131.6, 131.7, 131.8, 132.95, 133.02, 133.6, 133.6, 137.1, 137.12, 142.6, 142.7, 149.2, 152.6, 157.5 (C_Ar_, N-C_thiaz_), 166.5 (N-C=N), 169.3 (C=N), 169.4 (C=N), 172.5 (C=O), 172.6 (C=O) ppm. 

Anal. Calcd for C_24_H_27_ClN_4_OS: C, 65.80%; H, 6.90%; N, 10.96%; Found: C, 65.94%; H, 6.86%; N, 11.15%.

*General procedure for the synthesis of compounds* **16c**, **f**, **h**. A mixture of the corresponding compound **3c**, **f**, **h** (2 mmol), *o*-phenylenediamine (0.32 g, 3 mmol) and 15% hydrochloric acid (30 mL) was refluxed for 72 h. Then, the reaction mixture was cooled down, diluted with water (150 mL), and the obtained precipitate was filtered off, washed with water and dried. Then, the crystals were dissolved in 2-propanol and diluted with an aqueous 10% potassium carbonate solution. The formed precipitate was filtered off, washed with water and dried. Purification was performed by recrystallization from a mixture of toluene with hexane (1:1).

*N-[2-(1H-Benzo[d]imidazol-2-yl)ethyl]-N-(2,5-dimethylphenyl)-4-phenylthiazol-2-amine* (**16c**)

Yield 0.55 g (65 %), m.p. 95–97 °C; 

IR (KBr): ν 1538, 1621 (C=N), 3055 (NH) cm^−1^; 

^1^H NMR (400 MHz, CDCl_3_): *δ* 2.12 (s, 3H, CH_3_), 2.34 (s, 3H, CH_3_), 3.40–3.59 (m, 2H, CH_2_), 4.53, 4.67 (2br. s, 2H, NCH_2_), 6.72 (s, 1H, H_Ar_), 7.01 (s, 1H, SCH), 7.14–7.47 (m, 7H, H_Ar_), 7.54 (t, 2H, *J* = 7.5 Hz, H_Ar_), 8.00 (d, 2H, *J* = 7.7 Hz, H_Ar_), 12.39 (br. s, 1H, NH) ppm; 

^13^C NMR (101 MHz, CDCl_3_): *δ* 17.1 (CH_3_), 21.0 (CH_3_), 29.2 (CH_2_), 49.8 (NCH_2_), 102.5 (SCH), 122.1, 126.0, 128.3, 129.1, 129.6, 130.27, 132.3, 133.6, 134.6, 138.3, 141.8, 150.7, 153.2 (C_Ar_, C_benzimid_.), 172.4 (N-C=N) ppm; 

Anal. Calcd for C_26_H_24_N_4_S: C, 73.55%; H, 5.70%; N, 13.20%; Found: C, 73.71%; H, 5.93%; N, 13.37%.

*N-[2-(1H-Benzo[d]imidazol-2-yl)ethyl]-4-(4-chlorophenyl)-N-(2,5-dimethylphenyl)thiazol-2-amine* (**16f**) 

Yield 0.59 g (64 %), m.p. 85–86 °C; 

IR (KBr): ν 1537, 1620 (2C=N), 3051 (NH) cm^−1^; 

^1^H NMR (400 MHz, CDCl_3_): *δ* 2.04 (s, 3H, CH_3_), 2.11 (s, 3H, CH_3_), 3.36 (s, 2H, CH_2_), 4.16, 4.45 (2br. s, 2H, NCH_2_), 6.79 (s, 1H, 1H, H_Ar_), 7.08–7.27 (m, 5H, H_Ar_ + SCH), 7.39, 7.52 (2d, 2H, *J* = 6.1 Hz, H_Ar_), 7.45 (d, 2H, *J* = 8.1 Hz, H_Ar_), 7.91 (d, 2H, *J* = 7.8 Hz, H_Ar_), 12.30 (s, 1H, NH) ppm; 

^13^C NMR (101 MHz, CDCl_3_) *δ* 16.7 (CH_3_), 20.1 (CH_3_), 27.2 (CH_2_), 50.8 (NCH_2_), 103.6 (SCH), 110.8, 118.2, 120.9, 121.6, 127.4, 128.5, 129.3, 129.3, 131.5, 131.8, 132.8, 133.6, 134.2, 137.1, 142.8, 143.3, 149.4, 152.4 (C_Ar_, C_benzimid_.), 169.3 (N-C=N) ppm; 

Anal. Calcd for C_26_H_23_ClN_4_S: C, 68.03%; H, 5.05%; N, 12.21%; Found: C, 68.22%; H, 5.29%; N, 12.39%.

*N-[2-(1H-Benzo[d]imidazol-2-yl)ethyl]-4-(3,4-dichlorophenyl)-N-(2,5-dimethylphenyl)thiazol-2-amine* (**16h**) 

Yield 0.61 g (62 %), m.p. 100–101 °C; 

IR (KBr): ν 1533 (2C=N), 1621, 3051 (NH) cm^−1^; 

^1^H NMR (400 MHz, DMSO-*d*_6_): *δ* 2.04 (s, 3H, CH_3_), 2.10 (s, 3H, CH_3_), 3.30–3.34 (m, 2H, CH_2_), 4.13, 4.50 (br. s, 2H, NCH_2_), 6.78 (s, 1H, 1H, H_Ar_), 7.05–7.15 (m, 3H, H_Ar_ + SCH), 7.25 (d, 1H, *J* = 7.8 Hz, H_Ar_), 7.32 (s, 1H, H_Ar_), 7.39 (d, 1H, *J* = 6.7 Hz, H_Ar_), 7.52 (d, 1H, *J* = 7.9 Hz, H_Ar_), 7.65 (d, 1H, *J* = 8.4 Hz, H_Ar_), 7.87 (dd, 1H, *J* = 8.4, 1.8 Hz, H_Ar_), 8.13 (d, 1H, *J* = 1.8 Hz, H_Ar_), 12.30 (s, 1H, NH) ppm; 

^13^C NMR (101 MHz, DMSO-*d*_6_): ***δ*** 16.6 (CH_3_), 20.1 (CH_3_), 27.2 (CH_2_), 50.8 (NCH_2_), 105.0 (SCH), 110.7, 118.2, 120.9, 121.6, 125.8, 127.2, 129.3, 129.4, 129.6, 130.7, 131.4, 131.6, 132.8, 134.2, 135.3, 137.1, 142.7, 143.3, 148.0, 152.4 (C_Ar_, C_benzimid_.), 169.4 (N-C=N) ppm; 

Anal. Calcd for C_26_H_22_Cl_2_N_4_S: C, 63.29%; H, 4.49%; N, 11.35%; Found: C, 63.10%; H, 4.55%; N, 11.47%.

*General procedure for the synthesis of compounds* **17c**, **f**, **h**. A mixture of the corresponding compound **3c**, **f**, **h** (1 mmol), sulfanilamide (0.19 g, 1.1 mmol), triethylamine (0.30 g, 3 mmol) and DMF (5 mL) was stirred at room temperature for 0.5 h. HBTU (0.57 g, 1.5 mmol) was dissolved in DMF (3 mL) at room temperature in an inert atmosphere and then added to the reaction mixture over 15 min. The reaction mixture was stirred at room temperature for 72 h, then diluted with an aqueous 10% potassium carbonate (50 mL) solution. The obtained precipitate was filtered off, washed with water and dried. Purification was performed by column chromatography using hexane: ethyl acetate (1:2) as eluent. 

*3-[(2,5-Dimethylphenyl)(4-phenylthiazol-2-yl)amino]-N-(4-sulfamoylphenyl)propanamide* (**17c**)

Yield 0.43 g, 85%, R*_f_* = 0.62 (hexane: ethyl acetate (1:2)), m.p. 78–79 °C.

IR (KBr): ν (cm^−1^) 1538 (C=N), 1681 (C=O), 3253 (NH), 3600 (NH_2_) cm^−1^. 

^1^H NMR (400 MHz, DMSO-*d*_6_): *δ* 2.15 (s, 3H, CH_3_), 2.22 (s, 3H, CH_3_), 2.71–2.93 (m, 3H, COCH_2_), 4.11 (br. s, 2H, NCH_2_), 7.11, 7.15 (2s, 3H, H_Ar_, SCH), 7.24 (s, 2H, NH_2_), 7.25, 7.29 (2d, 2H, *J* = 6.9 Hz, H_Ar_), 7.38, 7.89 (t, 4H, *J* = 7.5 Hz, H_Ar_), 7.70, 7.74 (2d, 4H, *J* = 8.9 Hz, H_Ar_), 10.35 (s, 1H, NH) ppm. 

^13^C NMR (101 MHz, DMSO-*d*_6_): *δ* 16.5 (CH_3_), 16.6 (CH_3_), 20.2 (CH_3_), 34.9 (CH_2_CO), 48.1 (NCH_2_), 102.6 (SCH), 118.4, 125.5, 126.4, 127.3, 128.3, 128.6, 129.0, 129.1, 131.4, 132.8, 134.5, 134.6, 136.9, 138.0, 141.8, 142.6, 150.3 (C_Ar_, N-C_thiaz_), 169.0 (N-C=N), 169.1 (N-C=N), 169.6 (C=O) ppm. 

Anal. Calcd for C_26_H_22_Cl_2_N_4_S: C, 61.64%; H, 5.17%; N, 11.06%; Found: C, 61.37%; H, 5.05%; N, 10.98%.

*3-[(4-(4-Chlorophenyl)thiazol-2-yl)(2,5-dimethylphenyl)amino]-N-(4-sulfamoylphenyl)propanamide* (**17f**)

Yield 0.44 g, 81%, R*_f_* = 0.63 (hexane: ethyl acetate (1:2)), m.p. 126–127 °C. 

IR (KBr): ν 1533 (C=N), 1667 (C=O), 3248 (NH), 3600 (NH_2_) cm^−1^. 

^1^H NMR (400 MHz, DMSO-*d*_6_): *δ* 2.14 (s, 3H, CH_3_), 2.22 (s, 3H, CH_3_), 2.74–2.89 (m, 2H, COCH_2_), 4.19 (br. s, 2H, NCH_2_), 7.14 (s, 2H, H_Ar_), 7.18 (s, 1H, SCH), 7.25–7.29 (m, H, H_Ar_), 7.44, 7.90 (2d, 4H, *J* = 8.4 Hz, H_Ar_), 7.61, 7.66 (2d, 4H, *J* = 8.6 Hz, H_Ar_), 8.76 (br. s, 2H, NH_2_) ppm. 

^13^C NMR (101 MHz, DMSO-*d*_6_): *δ* 16.7 (CH_3_), 20.4 (CH_3_), 34.9 (CH_2_CO), 48.4 (NCH_2_), 103.6 (SCH), 118.3, 126.2, 127.4, 128.5, 129.3, 129.4, 131.6, 131.8, 133.0, 133.6, 137.2, 140.7, 141.8, 142.7, 149.3 (C_Ar_, N-C_thiaz_), 169.5 (N-C=N), 169.6 (C=O) ppm. 

Anal. Calcd for C_26_H_25_ClN_4_O_3_S_2_: C, 57.72%; H, 4.66%; N, 10.35%; Found: C, 57.86%; H, 4.79%; N, 10.51%.

*3-((4-(3,4-Dichlorophenyl)thiazol-2-yl)(2,5-dimethylphenyl)amino)-N-(4-sulfamoylphenyl)propanamide* (**17h**)

Yield 0.45 g, 78%, R*_f_* = 0.62 (hexane: ethyl acetate (1:2)), m.p. 98–99 °C. 

IR (KBr): ν 1532 (C=N), 1682 (C=O), 3253 (NH), 3653 (NH_2_) cm^−1^. 

^1^H NMR (400 MHz, DMSO-*d*_6_): *δ* 2.14 (s, 3H, CH_3_), 2.22 (s, 3H, CH_3_), 2.84 (t, 2H, *J* = 6.9 Hz, COCH_2_), 4.21 (br. s, 2H, NCH_2_), 7.14, 7.15 (2s, 2H, H_Ar_, SCH), 7.24 (s, 2H, NH_2_), 7.28 (d, 1H, *J* = 8.2 Hz, H_Ar_), 7.33 (s, 1H, H_Ar_), 7.64 (d, 1H, *J* = 8.4 Hz, H_Ar_), 7.70, 7.74 (2d, 4H, *J* = 8.9 Hz, H_Ar_), 7.87 (dd, 1H, *J* = 8.4, 1.5 Hz, H_Ar_), 8.12 (s, 1H, H_Ar_), 10.35 (s, 1H, NH) ppm. 

^13^C NMR (101 MHz, DMSO-*d*_6_): *δ* 16.7 (CH_3_), 20.4 (CH_3_), 35.0 (CH_2_CO), 48.3 (NCH_2_), 105.0 (SCH), 118.6, 125.7, 126.6, 127.2, 129.2, 129.5, 129.6, 130.7, 131.4, 131.6, 132.9, 135.3, 137.2, 138.2, 141.9, 142.6, 147.9 (C_Ar_, N-C_thiaz_), 169.6 (N-C=N), 169.8 (C=O) ppm.

Anal. Calcd for C_26_H_24_Cl_2_N_4_O_3_S_2_: C, 54.26%; H, 4.20%; N, 9.74%; Found: C, 54.08%; H, 4.12%; N, 9.83%.

### 3.2. Bacterial Strains and Culture Conditions

The multidrug-resistant and genetically defined isolates were obtained from the AR isolate bank at the Centre for Disease Control (CDC, United States). *S. aureus* TCH 1516 (USA300) was obtained from the American Type Culture Collection. Prior to the study, all strains were maintained in commercial cryopreservation systems at −80 °C. Bacterial strains were subcultured on Columbia Sheep Blood agar or Tryptic-Soy agar (Becton Dickenson, Franklin Lakes, NJ, USA). Fungal strains were cultured on Saburoud-Dextrose agar. Unless otherwise specified, all antimicrobial susceptibility studies with bacterial pathogens were performed in Cation-Adjusted Mueller–Hinton broth (CAMBH) for liquid cultures (Liofilchem, Italy). Antifungal studies were conducted using RPMI/MOPS broth. 

### 3.3. Minimal Inhibitory Concentration Determination

#### 3.3.1. Antibacterial Activity Characterization

The minimal inhibitory concentrations (MICs) of compounds **1–17** as well as various antibiotics were determined according to the recommendations of the Clinical and Laboratory Standards Institute (CLSI). The MICs for the compounds and comparator antibiotics were determined according to the testing standard broth microdilution methods described in CLSI document M07-A8 against the libraries of Gram-positive and Gram-negative pathogens. Compounds and antibiotics that were used as a control were dissolved in dimethyl sulfoxide (DMSO) to achieve a final concentration of 15–30 mg/mL. A series of compound dilutions were prepared in deep, polypropylene 96-well microplates to achieve 2× of concentrations (0.5–64 µg/mL) and were then transferred to the assay single-use, flat bottom plates. The prepared microplates were stored at −80 °C until the day of the experiment. 

A standardized bacterial inoculum was prepared using the colony suspension method. The inoculum suspension was diluted in sterile CAMBH to achieve final concentrations of approximately 5 × 10^5^ CFU/mL (range, 2 × 10^5^ to 8 × 10^5^ CFU/mL) in each well. The inoculum was transferred to the assay plates to achieve 1× assay concentration. Inoculated microdilution plates were incubated at 35 °C for 16 to 20 h in an ambient-air incubator within 15 min of the addition of the inoculum.

#### 3.3.2. Antifungal Activity Characterization

The MIC of compounds **1–17** as well as clinically approved antifungal drugs was determined by CLSI recommendations, described in document M27-A3 [84,85]. Briefly, before the experiments, multidrug-resistant *Candida* spp., strains were sub-cultured on Saburoud-Dextrose agar for 24 h at 35 °C. The colonies were suspended in sterile saline to reach approximately 5 × 10^6^ CFU/mL. Then, the fungal suspension is diluted in RPMI/MOPS broth to reach 5 × 10^5^ CFU/mL and microplates containing test compounds, prepared as described above are inoculated using a multichannel pipette. Inoculated microdilution plates were incubated at 35 °C for 16 to 20 h in an ambient-air incubator within 15 min of the addition of the inoculum.

### 3.4. Cell Lines and Culture Conditions

The non-small-cell human lung carcinoma A549 cells were obtained from American Type Culture Collection (Rockville, MD, USA). Caco-2 human corectal adenocarcinoma cells were obtained from Dr. Iliyan IIiev’s laboratory (Institute for Research in IBD, Weill Cornell Medicine of Cornell University). Cells were maintained in Dulbecco’s Modified Eagle Medium/Nutrient Mixture F-12 (DMEM/F-12) (Gibco, Waltham, MA, USA) supplemented with 10% fetal bovine serum (10% FBS) (Gibco, Waltham, MA, USA) and 100 U/mL penicillin and 100 μg/mL streptomycin (P/S). Cells were cultured at 37 °C humidified atmosphere containing 5% of CO_2_. Cells were fed every 2–3 days and subculture upon reaching 70–80% confluence.

### 3.5. In Vitro Cytotoxic Activity Determination

The viability of A549 and Caco-2 cells after the treatment with compounds or cisplatin that served as cytotoxicity control was evaluated by using commercial MTT (3-[4,5-methylthiazol-2-yl]-2,5-diphenyltetrazolium bromide) assay (ThermoFisher Scientific, United States). Briefly, cells were plated in the flat-bottomed 96-well microplates (1 × 10^4^ cells/well) and incubated overnight to facilitate the attachment. The test compounds were dissolved in hybridoma-grade DMSO (Sigma-Aldrich, St. Louis, MO, USA) and then further serially diluted in cell culture media containing 0.25 % DMSO to achieve 100 µM of each compound. 

Subsequently, the media from the cells was aspirated and the compounds were added to the microplates. The cells were incubated at 37 °C, 5% CO_2_ for 24 h. After incubation, a 10 μL of Vybrant^®^ MTT Cell Proliferation Reagent (ThermoFisher Scientific) was added, and cells were further incubated for 4 h. After incubation, the media was aspirated, and the resulting formazan was solubilized by the addition of 100 μL of DMSO. The absorbance was then measured at 570 nm by using a microplate reader (Multiscan, ThermoFisher Scientific). The following formula was used to calculate the % of A549 viability: ([AE-AB]/[AC-AB]) × 100%. AE, AC, and AB were defined as the absorbance of experimental samples, untreated samples, and blank controls, respectively. The experiments were performed in triplicates.

### 3.6. Statistical Analysis

The results are expressed as mean ± standard deviation (SD). Statistical analyses were performed with Prism (GraphPad Software, San Diego, CA, USA), using Kruskal–Wallis test and two-way ANOVA. *p* < 0.05 was accepted as significant.

## 4. Conclusions

In the present study, a series of 2-aminothiazole derivatives containing *N*-2,5-dimethyl phenyl and β-alanine moieties in the molecules were synthesized and evaluated for their in vitro antimicrobial activity using a panel of multidrug-resistant bacterial and fungal pathogens with emerging and genetically defined resistance mechanisms. In addition, we characterized the cytotoxic and anticancer properties using A549 and Caco-2 pulmonary and corectal adenocarcinoma models. 

The results revealed that thiazoles with the 4-aryl substituted showed profound and selective antimicrobial activity against Gram-positive pathogens. Compound **3j** possessed the most potent activity against tested strains multidrug-resistant *S. aureus* and *E. faecium* (MIC 1–2 µg/mL). Strikingly, compounds **3h, 3j** and **7** showed antibacterial activity against especially clinically challenging tedizolid/linezolid-resistant *S. aureus* strains. 

On the other hand, the modified carboxyl group in thiazoles **8f**, **9f** and **14f** provided promising antifungal properties against drug-resistant *Candida* strains, including *C. glabrata, C. parapsilosis* and *C. hemulonii.* Moreover, ester **8f** was found to have broader antifungal activity on multiple *Candida* species (1–8 µg/mL) including the emerging fungal pathogen *C. auris*. 

During the anticancer activity study, a structure-dependent anticancer activity against A549 and Caco-2 cells was observed. Structure–activity relation analysis revealed naphthoquinone-fused thiazole **7** structure and 2,4-diCl-C_6_H_3_ moiety **3j** to provide broad-spectrum anticancer against both A549 and Caco-2 cells. 

Compounds based on 2-aminothiazole derivatives containing *N*-2,5-dimethyl phenyl and β-alanine moieties could be further explored as novel scaffolds for the development of novel and highly active compounds targeting multidrug-resistant pathogens, including tedizolid/linezolid-resistant *S. aureus* as well as vancomycin-resistant *Enterococcus.* Furthermore, a series of novel compounds based on **8f** could be further explored as novel antifungals targeting challenging and multidrug-resistant *Candida auris.* Further studies are needed to better understand the safety, pharmacological properties as well as cellular targets of 2-aminothiazole derivatives **1–17**. 

## Figures and Tables

**Table 1:** In vitro antibacterial activity of compounds **1**–**17** against the representative, multidrug-resistant Gram-positive and Gram-negative bacterial strains harbouring genetically defined resistance mechanisms.

Compounds	Minimal Inhibitory Concentration (µg/mL)					
	*S. aureus* TCH 1516 ^1^	*E. faecium* AR-0783 ^2^	*K. pneumoniae* AR-0139 ^3^	*E. coli* AR-0149 ^4^	*A. baumannii* AR-0037 ^5^	*P. aeruginosa* AR-0100 ^6^
**1**	>64	>64	>64	>64	>64	>64
**2**	>64	>64	>64	>64	>64	>64
**3a**	>64	>64	>64	>64	>64	>64
**3b**	>64	>64	>64	>64	>64	>64
**3c**	>64	>64	>64	>64	>64	>64
**3d**	>64	>64	>64	>64	>64	>64
**3e**	>64	>64	>64	>64	>64	>64
**3f**	>64	>64	>64	>64	>64	>64
**3g**	>64	>64	>64	>64	>64	>64
**3h**	8	16	>64	>64	>64	>64
**3i**	>64	>64	>64	>64	>64	>64
**3j**	2	2	>64	>64	>64	>64
**3k**	>64	>64	>64	>64	>64	>64
**4i**	>64	>64	>64	>64	>64	>64
**4j**	>64	>64	>64	>64	>64	>64
**4k**	>64	>64	>64	>64	>64	>64
**5**	>64	>64	>64	>64	>64	>64
**6**	>64	>64	>64	>64	>64	>64
**7**	1	8	>64	>64	>64	>64
**8f**	>64	>64	>64	>64	>64	>64
**9f**	>64	>64	>64	>64	>64	>64
**10f**	>64	>64	>64	>64	>64	>64
**11f**	16	>64	>64	>64	>64	>64
**12f**	>64	>64	>64	>64	>64	>64
**13f**	>64	>64	>64	>64	>64	>64
**14f**	>64	>64	>64	>64	>64	>64
**15f**	>64	>64	>64	>64	>64	>64
**16c**	>64	>64	>64	>64	>64	>64
**16f**	>64	>64	>64	>64	>64	>64
**16h**	>64	>64	>64	>64	>64	>64
**17c**	>64	>64	>64	>64	>64	>64
**17f**	>64	>64	>64	>64	>64	>64
**17h**	>64	>64	>64	>64	>64	>64
Vancomycin	2	64	N/A	N/A	N/A	N/A
Ampicillin	>64	64	>64	64	>64	>64
Daptomycin	1	4	N/A	N/A	N/A	N/A
Aztreonam	N/A	N/A	4	2	2	16
Meropenem	N/A	N/A	8	16	32	32
Imipenem	N/A	N/A	>64	>64	>64	>64

^1^*S. aureus* TCH 1516 *mecA*, USA300 lineage. ^2^ *E. faecium* AR-0783 *VanB*. ^3^ *K. pneumoniae* AR-0193 *ac(6′)-IIa, armA, ARR-3, cmlA1, CMY-4, CTX-M-15, dfrA1, fosA, mph(E), msr(E), NDM-1, oqxA, oqxB, OXA-10, SHV-11, strA, strB, sul1, sul2*. ^4^ *E. coli* AR-0149 *CMY-42, NDM-7*. ^5^ *A. baumannii* AR-0037 *NDM-1, OXA-94, sul2*. ^6^ *P. aeruginosa* AR-0100 *aac(3)-Id, aadA6, dfrB5, OXA-50, PAO, sul1, VIM-2*.

**Table 2:** In vitro antibacterial activity of the most promising thiazole derivatives **3h**, **3j** and **7** against tedizolid/linezolid resistant *S. aureus* strains.

Compounds	*S. a* AR-0701	*S. a* AR-0702	*S. a* AR-0703	*S. a* AR-0704	*S. a* AR-0705
**3h**	4	8	8	32	8
**3j**	2	2	1	2	2
**7**	4	16	8	8	8
Vancomycin	1	2	1	1	1
Daptomycin	0.5	1	0.5	0.5	1
Linezolid	16	16	32	8	16

Abbreviations: *S. a*—*Staphylococcus aureus (S. aureus)*.

**Table 3:** In vitro antifungal activity of compounds **1–17** against the representative, multidrug-resistant fungal pathogens.

Compounds	Minimal Inhibitory Concentration (µg/mL)				
	*C. auris* AR-0383	*C. albicans*	*C. glabrata* AR-315	*C. parapsilosis* AR-0335	*C. haemulonii* AR-395
**1**	>64	>64	>64	>64	>64
**2**	>64	>64	>64	>64	>64
**3a**	>64	>64	>64	>64	>64
**3b**	>64	>64	>64	>64	>64
**3c**	>64	>64	>64	>64	>64
**3d**	>64	>64	>64	>64	>64
**3e**	>64	>64	>64	>64	>64
**3f**	>64	>64	>64	>64	>64
**3g**	>64	>64	>64	>64	>64
**3h**	>64	>64	>64	>64	>64
**3i**	>64	>64	>64	>64	>64
**3j**	>64	>64	>64	>64	>64
**3k**	>64	>64	>64	>64	>64
**4i**	>64	>64	>64	>64	>64
**4j**	>64	>64	>64	>64	>64
**4k**	>64	>64	>64	>64	>64
**5**	>64	>64	>64	>64	>64
**6**	>64	>64	>64	>64	>64
**7**	>64	>64	>64	>64	>64
**8f**	2	1	4	8	2
**9f**	>64	8	4	4	2
**10f**	>64	>64	>64	>64	>64
**11f**	>64	>64	>64	>64	>64
**12f**	>64	>64	>64	>64	>64
**13f**	>64	>64	>64	>64	>64
**14f**	>64	8	16	32	32
**15f**	>64	>64	>64	>64	>64
**16c**	>64	>64	>64	>64	>64
**16f**	>64	>64	>64	>64	>64
**16h**	>64	>64	>64	>64	>64
**17c**	>64	>64	>64	>64	>64
**17f**	>64	>64	>64	>64	>64
**17h**	>64	>64	>64	>64	>64
Amphotericin B	<0.5	<0.5	<0.5	<0.5	<0.5
Fluconazole	32	16	16	8	4
Posaconazole	0.5	1	0.5	1	1

## Data Availability

Not applicable.

## Supplementary Materials

The following supporting information can be downloaded at: https://www.mdpi.com/article/10.3390/antibiotics12020220/s1, Figure S1: ^1^H-NMR of compound **1**, Figure S2: ^13^C-NMR of compound **1**, Figure S3: ^1^H-NMR of compound **2**, Figure S4: ^13^C-NMR of compound **2**, Figure S5: ^1^H-NMR of compound **3a**, Figure S6: ^13^C-NMR of compound **3a**, Figure S7: ^1^H-NMR of compound **3b**, Figure S8: ^13^C-NMR of compound **3b**, Figure S9: ^1^H-NMR of compound **3c**, Figure S10: ^13^C-NMR of compound **3c**, Figure S11: ^1^H-NMR of compound **3d**, Figure S12: ^13^C-NMR of compound **3d**, Figure S13: ^1^H-NMR of compound **3e**, Figure S14: ^13^C-NMR of compound **3e**, Figure S15: ^1^H-NMR of compound **3f**, Figure S16: ^13^C-NMR of compound **3f**, Figure S17: ^1^H-NMR of compound **3g**, Figure S18: ^13^C-NMR of compound **3g**, Figure S19: ^1^H-NMR of compound **3h**, Figure S20: ^13^C-NMR of compound **3h**, Figure S21: ^1^H-NMR of compound **3i**, Figure S22: ^13^C-NMR of compound **3i**, Figure S23: ^1^H-NMR of compound **3j**, Figure S24: ^13^C-NMR of compound **3j**, Figure S25: ^1^H-NMR of compound **3k**, Figure S26: ^13^C-NMR of compound **3k**, Figure S27: ^1^H-NMR of compound **4i**, Figure S28: ^13^C-NMR of compound **4i**, Figure S29: ^1^H-NMR of compound **4j**, Figure S30: ^13^C-NMR of compound **4j**, Figure S31: ^1^H-NMR of compound **4k**, Figure S32: ^13^C-NMR of compound **4k**, Figure S33: ^1^H-NMR of compound **5,** Figure S34: ^13^C-NMR of compound **5**, Figure S35: ^1^H-NMR of compound **6**, Figure S36: ^13^C-NMR of compound **6**, Figure S37: ^1^H-NMR of compound **7**, Figure S38: ^13^C-NMR of compound **7**, Figure S39: ^1^H-NMR of compound **8f**, Figure S40: ^13^C-NMR of compound **8f**, Figure S41: ^1^H-NMR of compound **9f**, Figure S42: 13C-NMR of compound **9f**, Figure S43: ^1^H-NMR of compound **10f**, Figure S44: ^13^C-NMR of compound **10f**, Figure S45: ^1^H-NMR of compound **11f**, Figure S46: ^13^C-NMR of compound **11f**, Figure S47: ^1^H-NMR of compound **12f**, Figure S48: ^13^C-NMR of compound **12f**, Figure S49: ^1^H-NMR of compound **13f**, Figure S50: ^13^C-NMR of compound **13f**, Figure S51: ^1^H-NMR of compound **14f**, Figure S52: ^13^C-NMR of compound **14f**, Figure S53: ^1^H-NMR of compound **15f**, Figure S54: ^13^C-NMR of compound **15f**, Figure S55: ^1^H-NMR of compound **16c**, Figure S56: ^13^C-NMR of compound **16c**, Figure S57: ^1^H-NMR of compound **16f**, Figure S58: ^13^C-NMR of compound **16f**, Figure S59: ^1^H-NMR of compound **16h**, Figure S60: ^13^C-NMR of compound **16h**, Figure S61: ^1^H-NMR of compound **17c**, Figure S62: ^13^C-NMR of compound **17c**, Figure S63: ^1^H-NMR of compound **17f**, Figure S64: ^13^C-NMR of compound **17f**, Figure S65: ^1^H-NMR of compound **17h**, Figure S66: ^13^C-NMR of compound **17h.** References [86,87] are cited in the supplementary materials.
