# Supplementary material for: Synthesis and Development of N-2,5-Dimethylphenylthioureido Acid Derivatives as Scaffolds for New Antimicrobial Candidates Targeting Multidrug-Resistant Gram-Positive Pathogens
Source: Antibiotics (Basel). 2023 Jan 20;12(2):220. doi: 10.3390/antibiotics12020220 (PMC9952208; doi:10.3390/antibiotics12020220)
Supplement: Supplementary file 1 [file antibiotics-12-00220-s001.zip › antibiotics-2158541-supplementary.pdf]

Supplementary Materials

Synthesis and Development of *N*-2,5-Dimethylphenylthioureido Acid Derivatives as Scaffolds for New Antimicrobial Candidates Targeting Multi-Drug-Resistant Gram-Positive Pathogens

Povilas Kavaliauskas [1,2,3,4], Birutė Grybaitė [1], Rita Vaickelionienė [1], Birutė Sapijanskaitė-Banevič [1], Kazimieras Anusevičius [1]*, Agnė Kriaučiūnaitė [1], Gabrielė Smailienė [1], Vidmantas Petraitis [2,4], Rūta Petraitienė [2,4], Ethan Naing [2], Andrew Garcia [3], and Vytautas Mickevičius [1]

[1] Department of Organic Chemistry, Kaunas University of Technology, Radvilėnų Rd. 19, LT-50254 Kaunas, Lithuania

[2] Weill Cornell Medicine of Cornell University, 527 East 68th Street, New York, NY 10065, USA
[3] Institute for Genome Sciences, School of Medicine, University of Maryland, 655 W. Baltimore Street, Baltimore, MD 21201, USA
[4] Institute of Infectious Diseases and Pathogenic Microbiology, Birštono Str. 38A, LT-59116 Prienai, Lithuania
* Correspondence: kazimieras.anusevicius@ktu.lt; Tel.: +370-646 21841

NMR Spectra (1-17, in DMSO-d_6 *or $CDCl_3$, Figures S1-S66*)

3-[Carbamothioyl(2,5-dimethylphenyl)amino]propanoic acid (**1**)

Figure S1. ^{1}H NMR of compound **1.** Additional acetic acid peaks at 1.96 ppm in Figure S1 and 20.92; 176.47 ppm in Figure S2 are observed and identified based on chemical shifts reported in the publication [1,2].

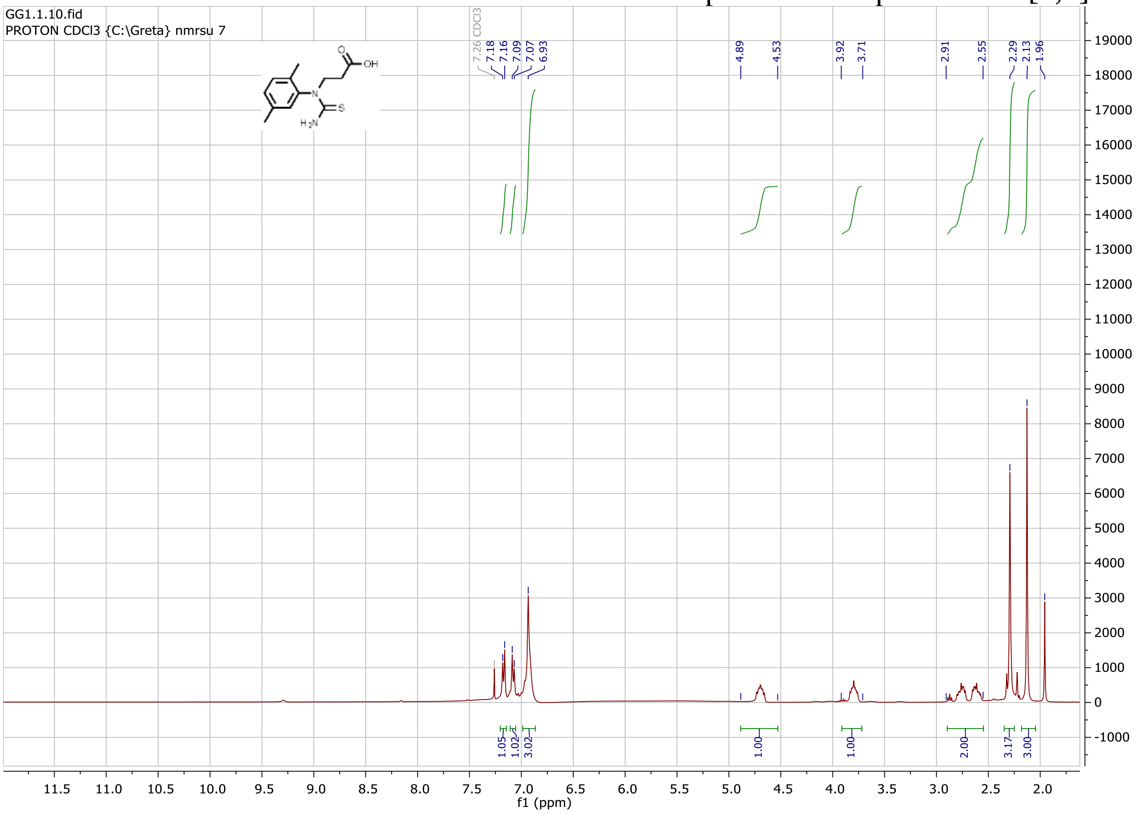

Figure S2. ^{13}C NMR of compound **1.**

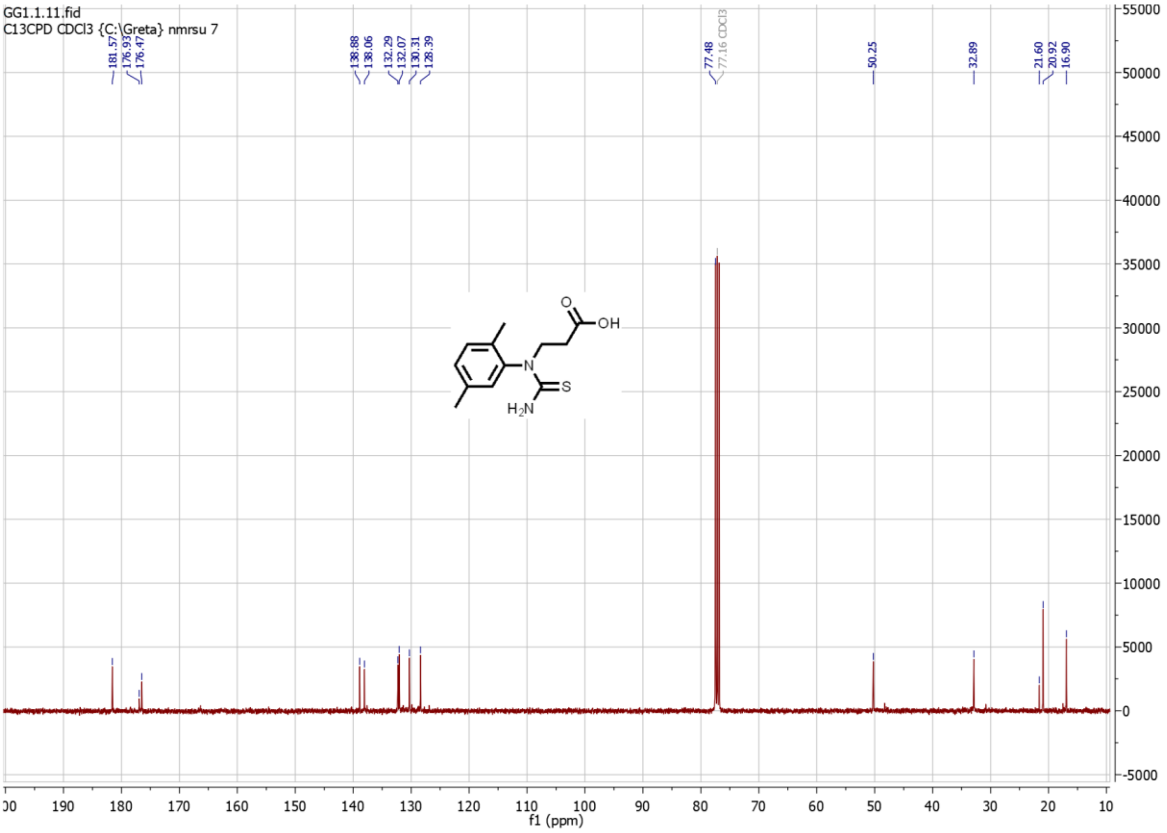

3-[(2,5-Dimethylphenyl)(4-oxo-4,5-dihydro-1,3-thiazol-2-yl)amino]propanoic acid **(2)**

Figure S3. ^{1}H NMR of compound **2.**

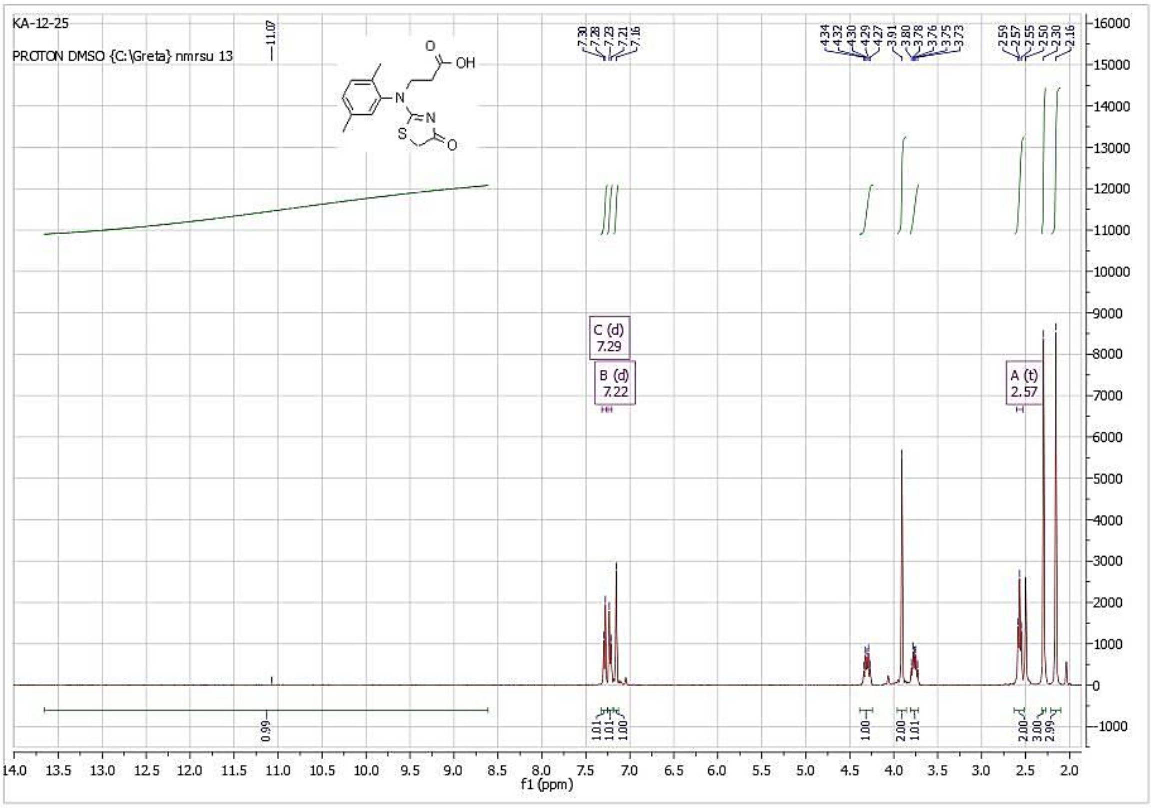

Figure S4. ^{13}C NMR of compound **2.**

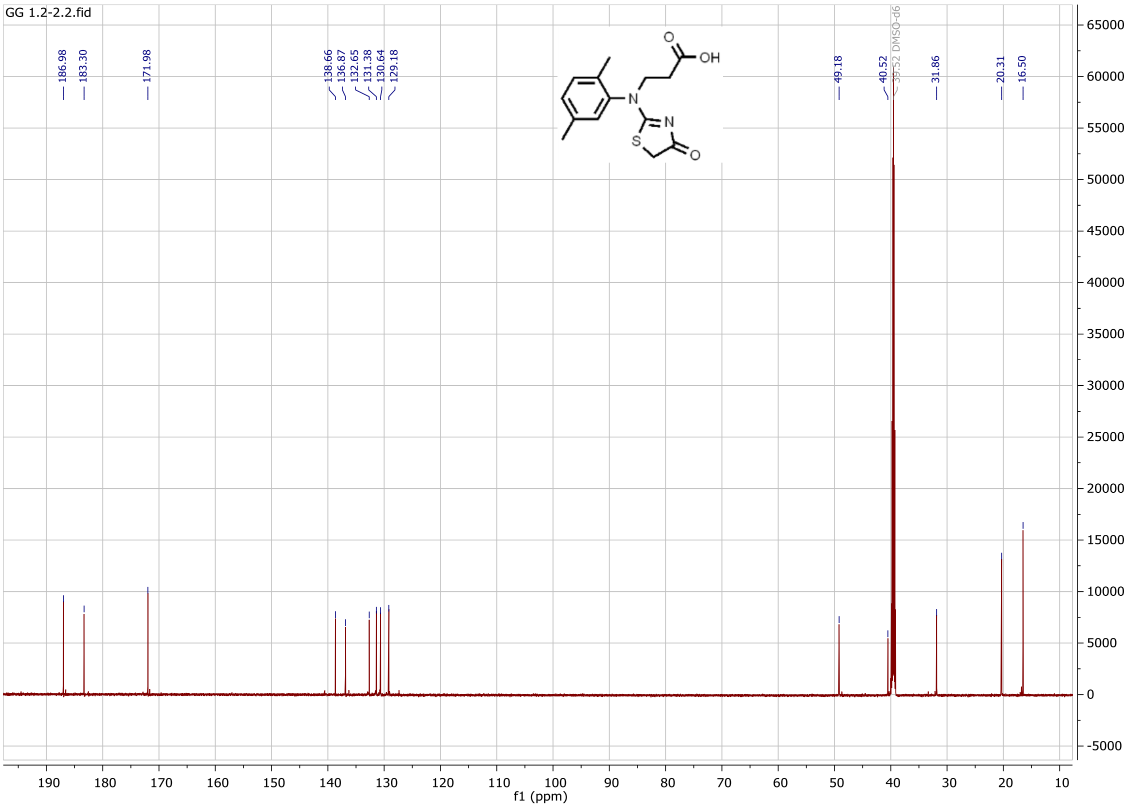

3-[(2,5-Dimethylphenyl)(1,3-thiazol-2-yl)amino]propanoic acid **(3a)**

Figure S5. ^{1}H NMR of compound **3a**. A broad peak for water at 3.33 ppm is observed in Figure S5 and identified by chemical shifts reported in the publication [1,2].

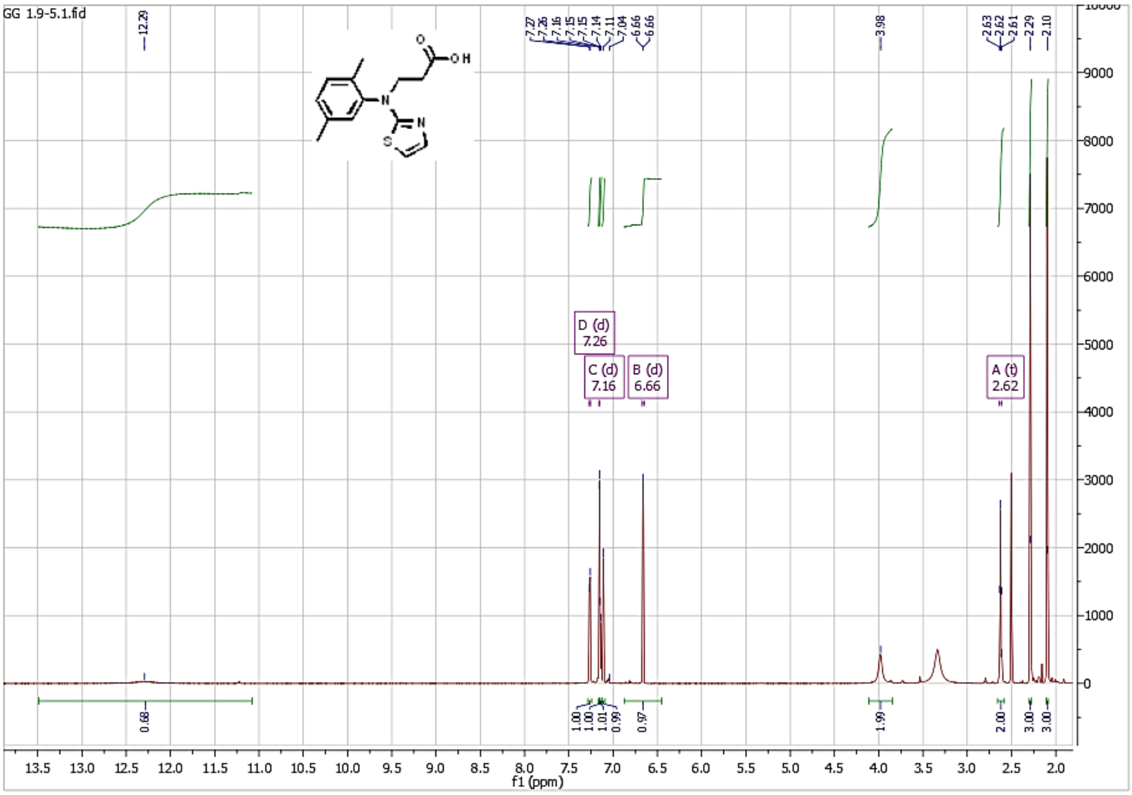

Figure S6. ^{13}C NMR of compound **3a.**

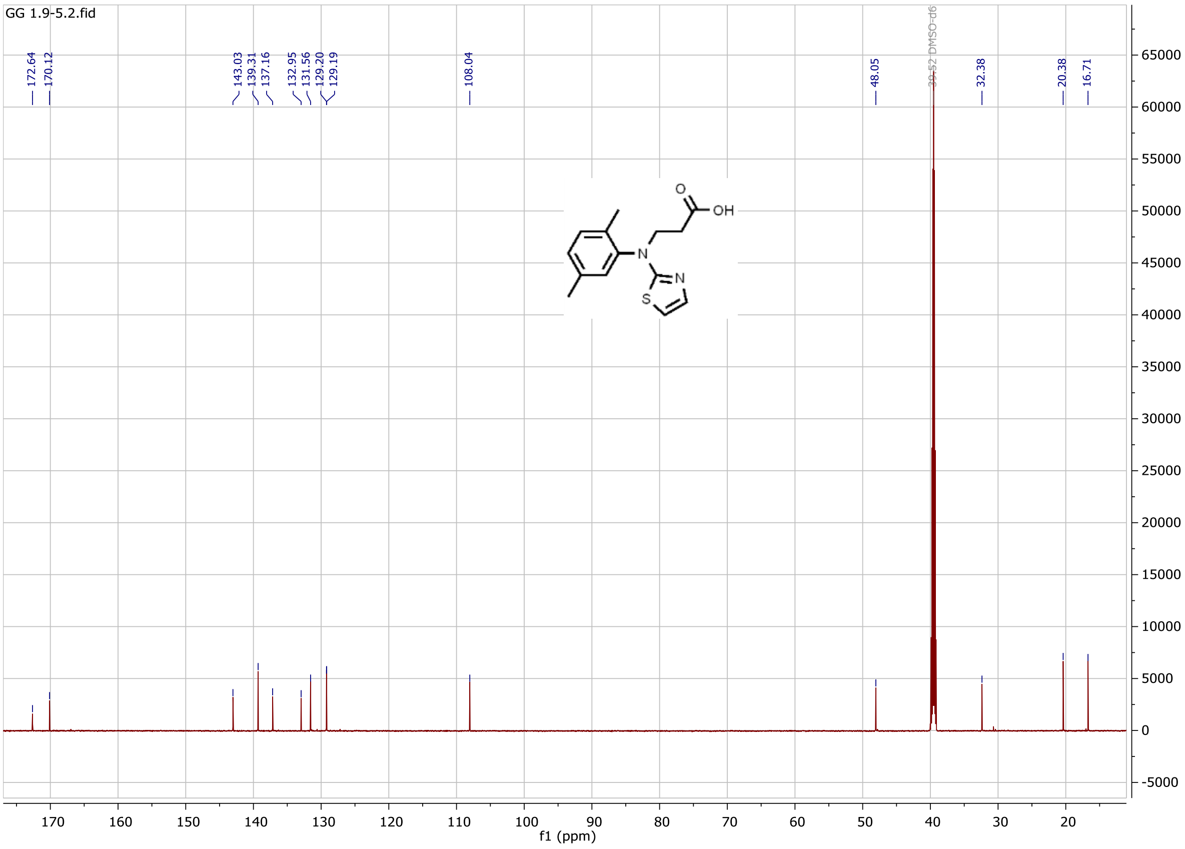

3-[(2,5-Dimethylphenyl)(4-methyl-1,3-thiazol-2-yl)amino]propanoic acid **(3b)**

Figure S7. ^{1}H NMR of compound **3b.**

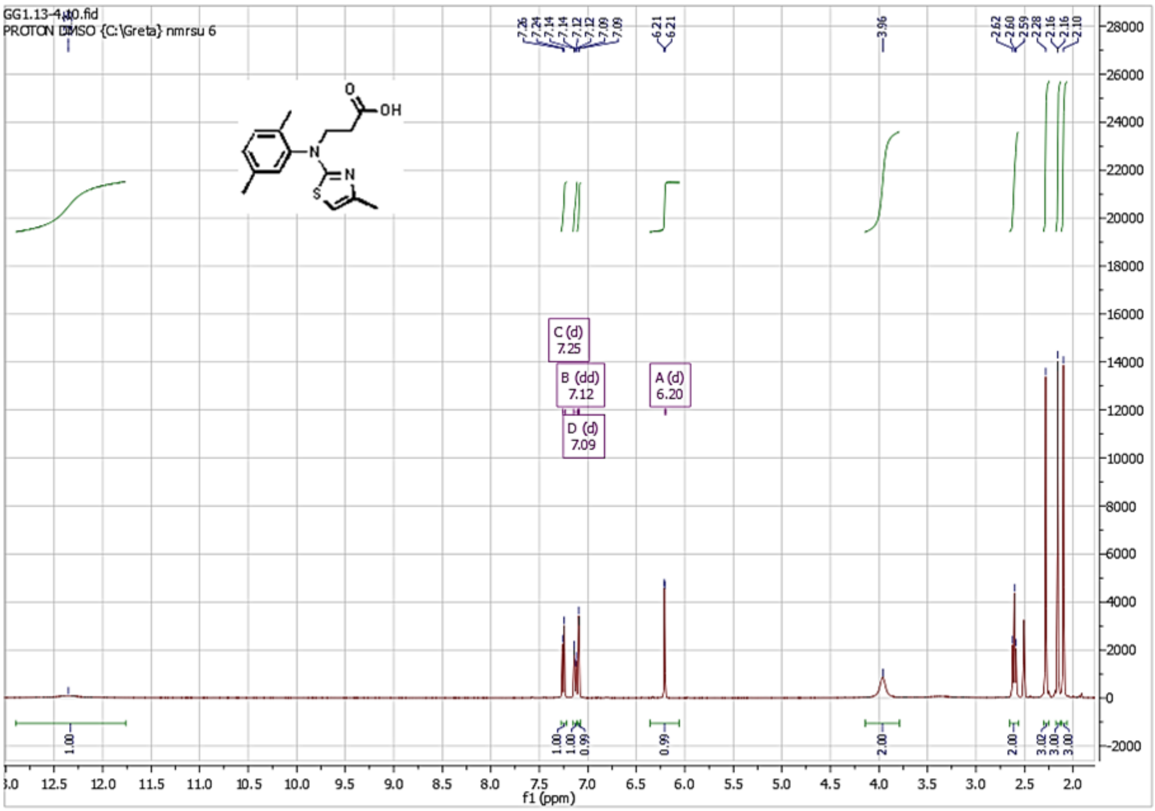

Figure S8. ^{13}C NMR of compound **3b.**

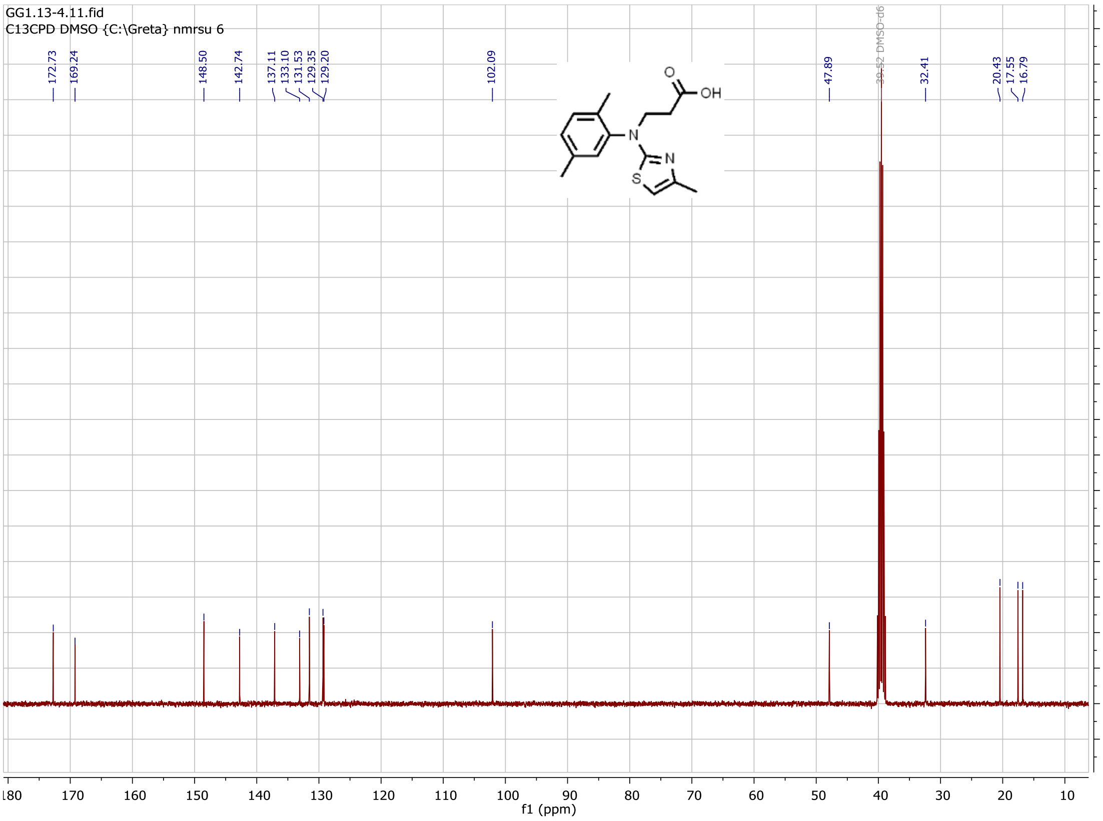

General synthesis of compounds 3c-k

3-[(2,5-Dimethylphenyl)(4-phenyl-1,3-thiazol-2-yl)amino]propanoic acid **(3c)**

Figure S9. ^{1}H NMR of compound **3c.**

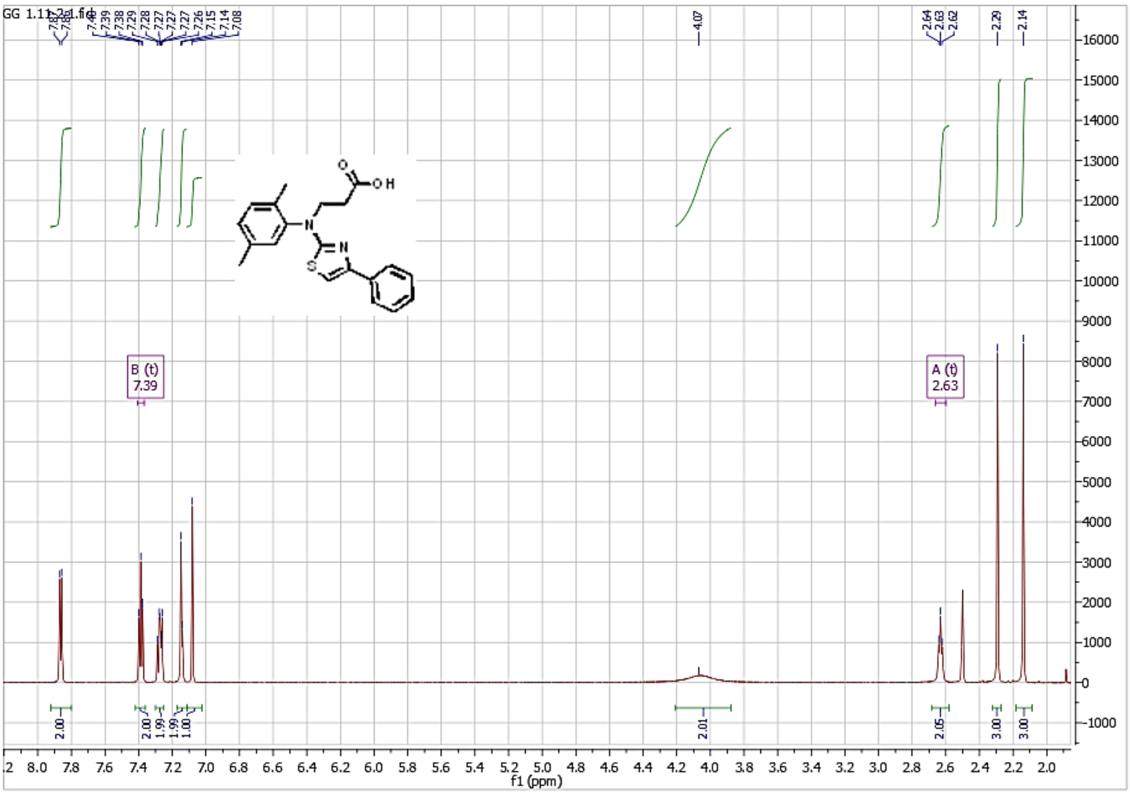

Figure S10. ^{13}C NMR of compound **3c.**

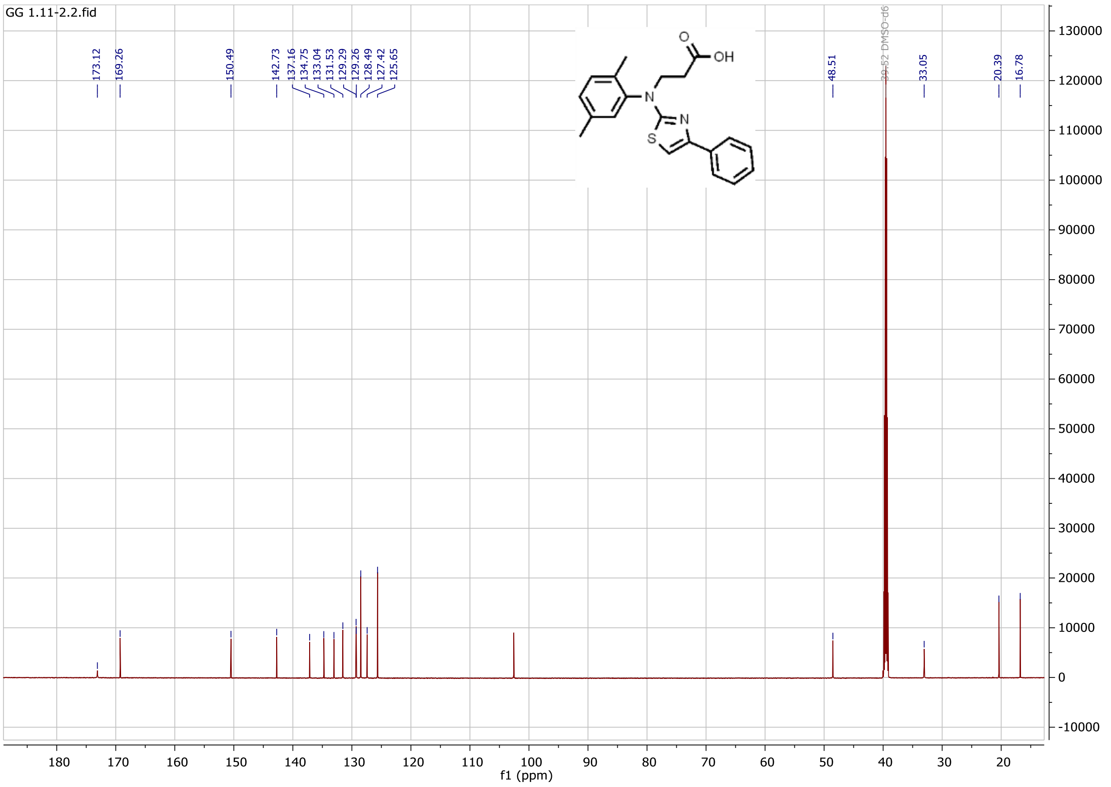

3-{(2,5-Dimethylphenyl)[4-(4-fluorophenyl)-1,3-thiazol-2-yl]amino}propanoic acid (**3d)**

Figure S11. ^{1}H NMR of compound **3d.**

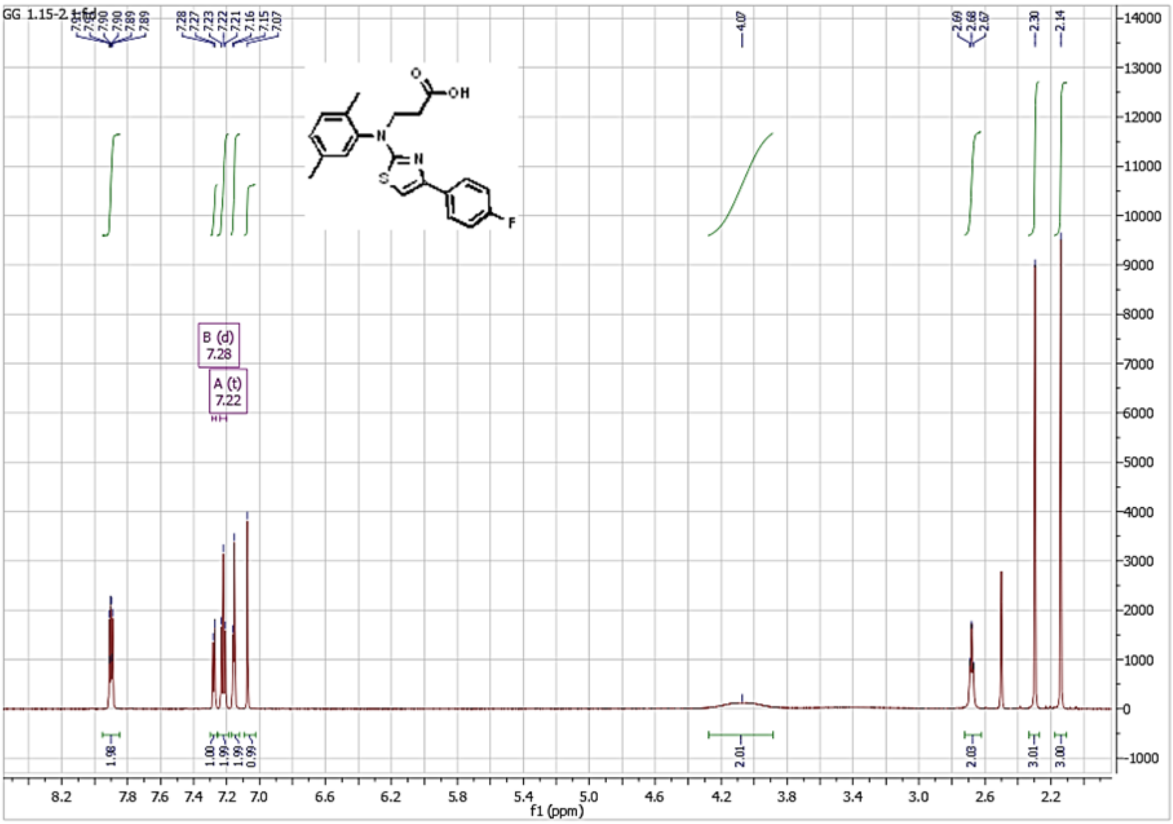

Figure S12. ^{13}C NMR of compound **3d.**

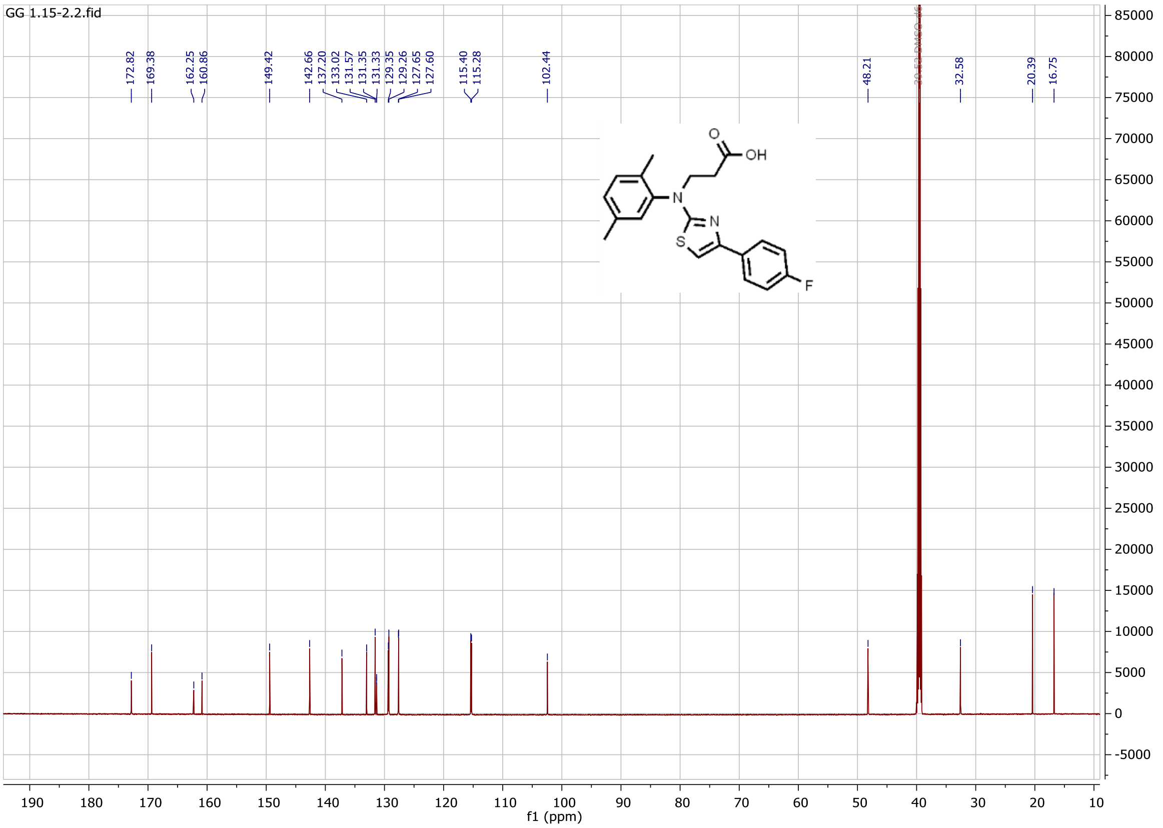

3-{[4-(4-Cyanophenyl)-1,3-thiazol-2-yl](2,5-dimethylphenyl)amino}propanoic acid **(3e)**

Figure S13. ^{1}H NMR of compound **3e.** A broad peak at 3.33 ppm for water is observed in Figure S13 and identified by chemical shifts reported in the publication [1,2].

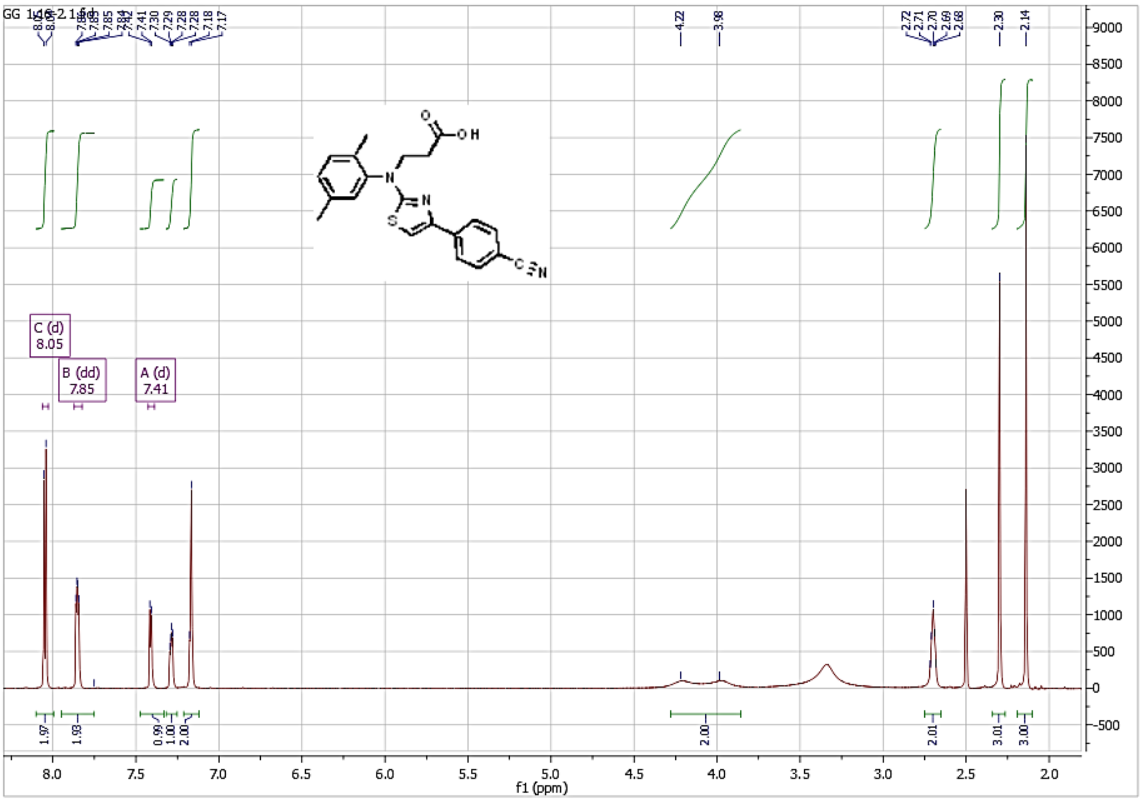

Figure S14. ^{13}C NMR of compound **3e.**

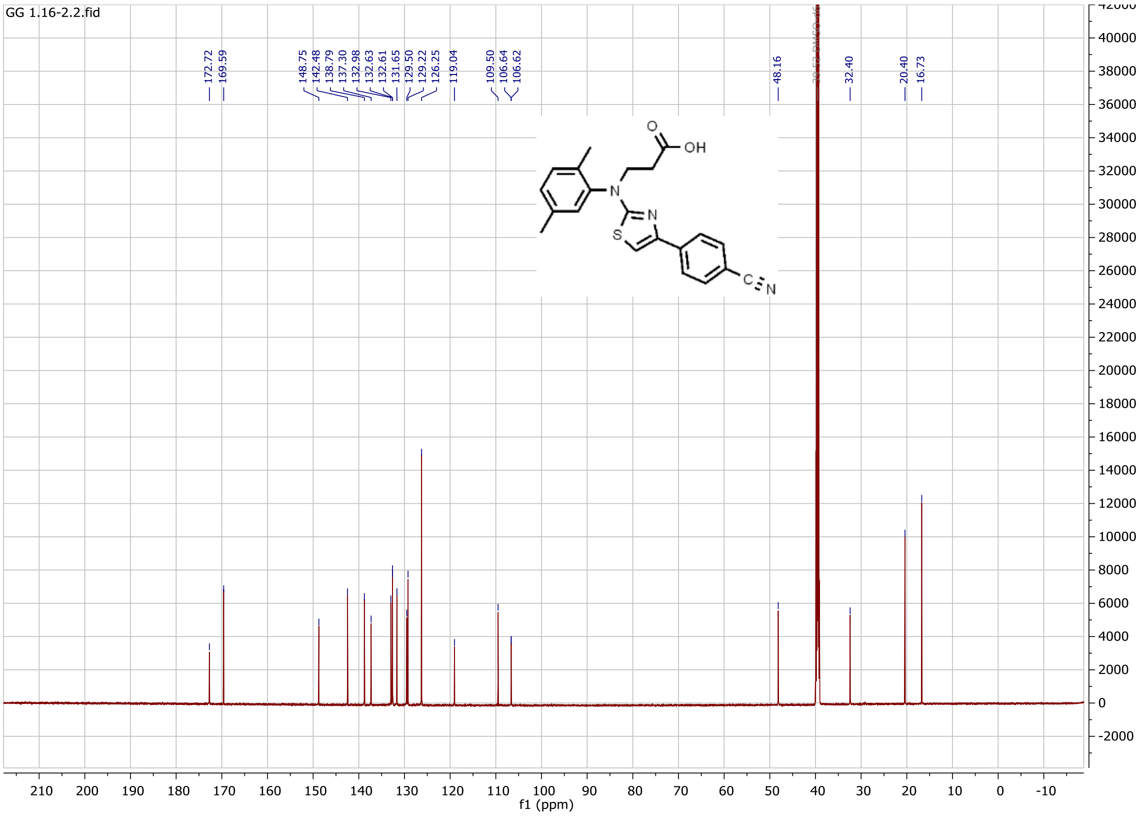

3-{[4-(4-Chlorophenyl)-1,3-thiazol-2-yl](2,5-dimethylphenyl)amino}propanoic acid **(3f)**

Figure S15. ^{1}H NMR of compound **3f.**

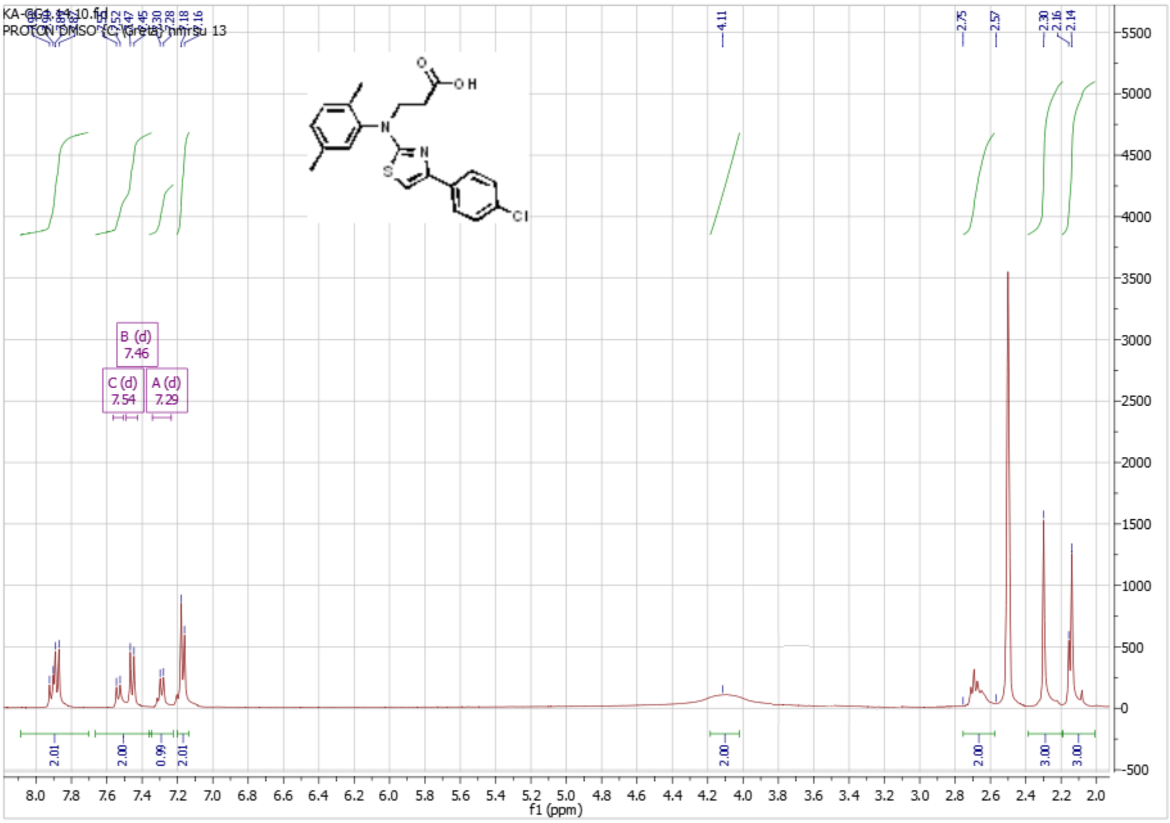

Figure S16. ^{13}C NMR of compound **3f.**

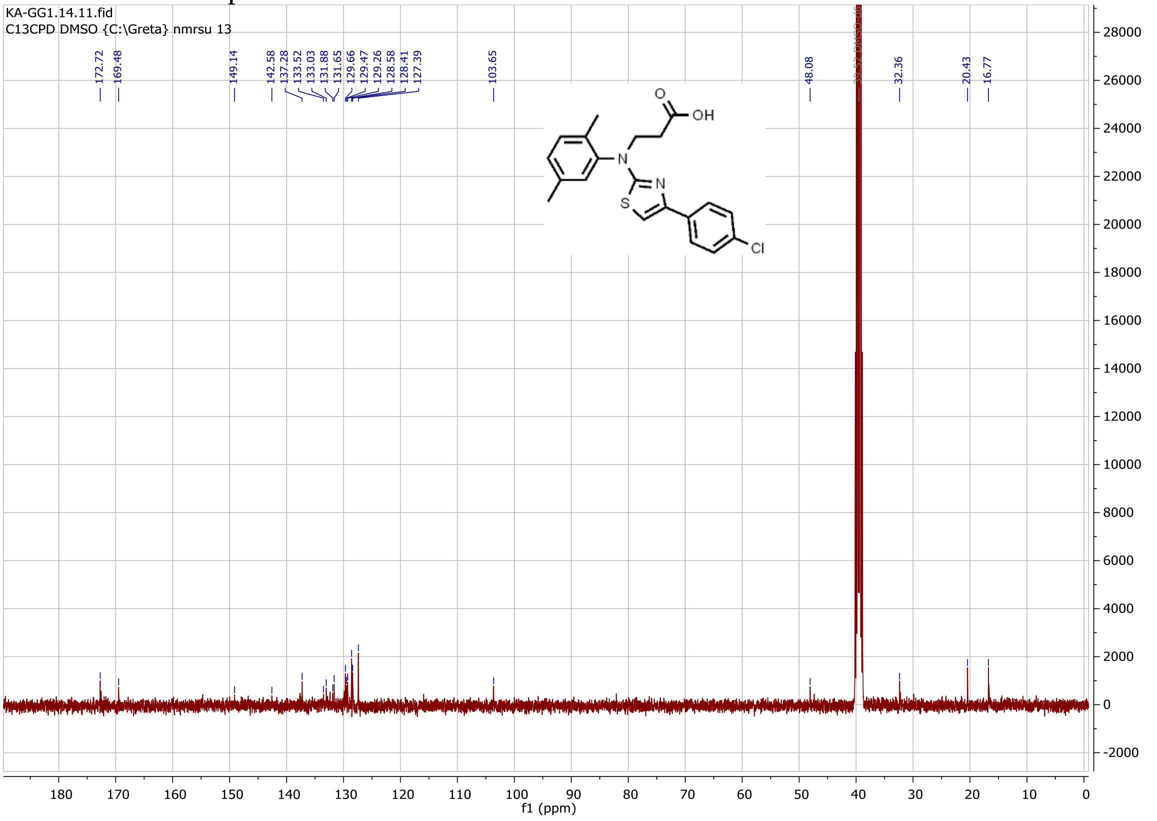

3-{(2,5-Dimethylphenyl)[4-(4-nitrophenyl)-1,3-thiazol-2-yl]amino}propanoic acid **(3g)**

Figure S17. ^{1}H NMR of compound **3g.**

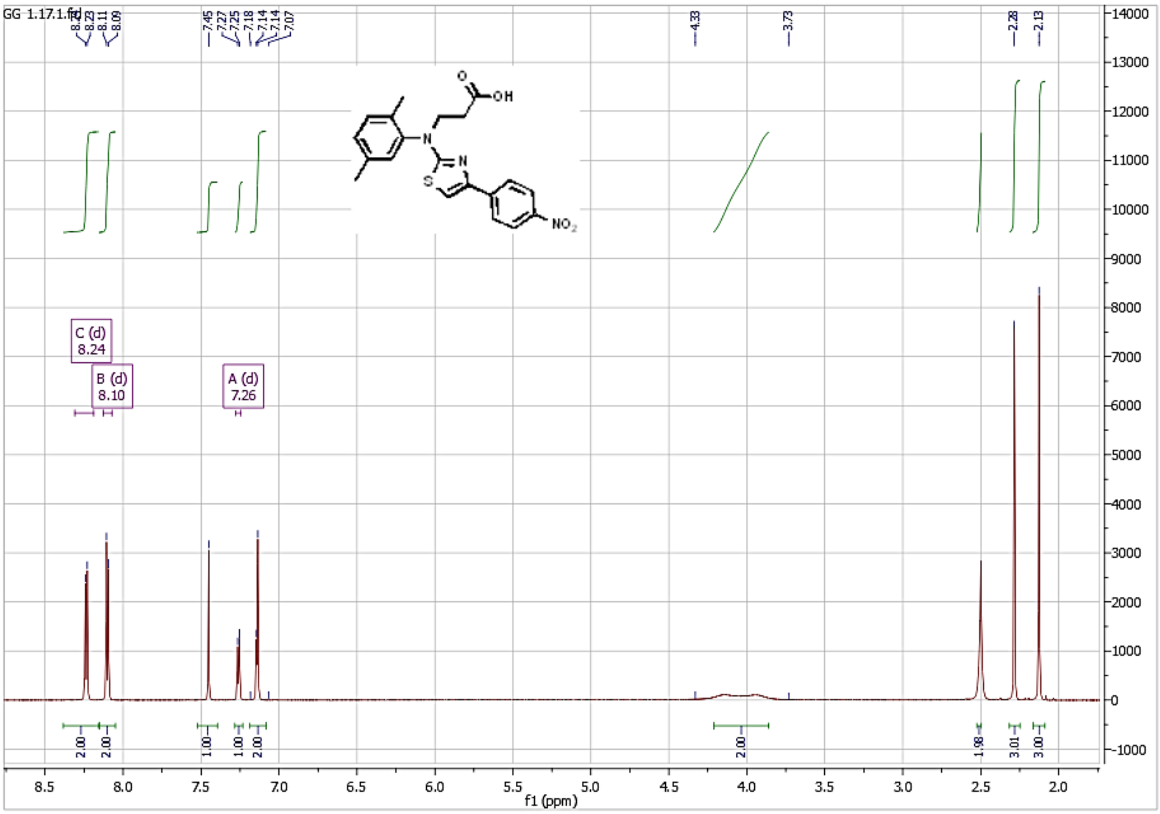

Figure S18. ^{13}C NMR of compound **3g.**

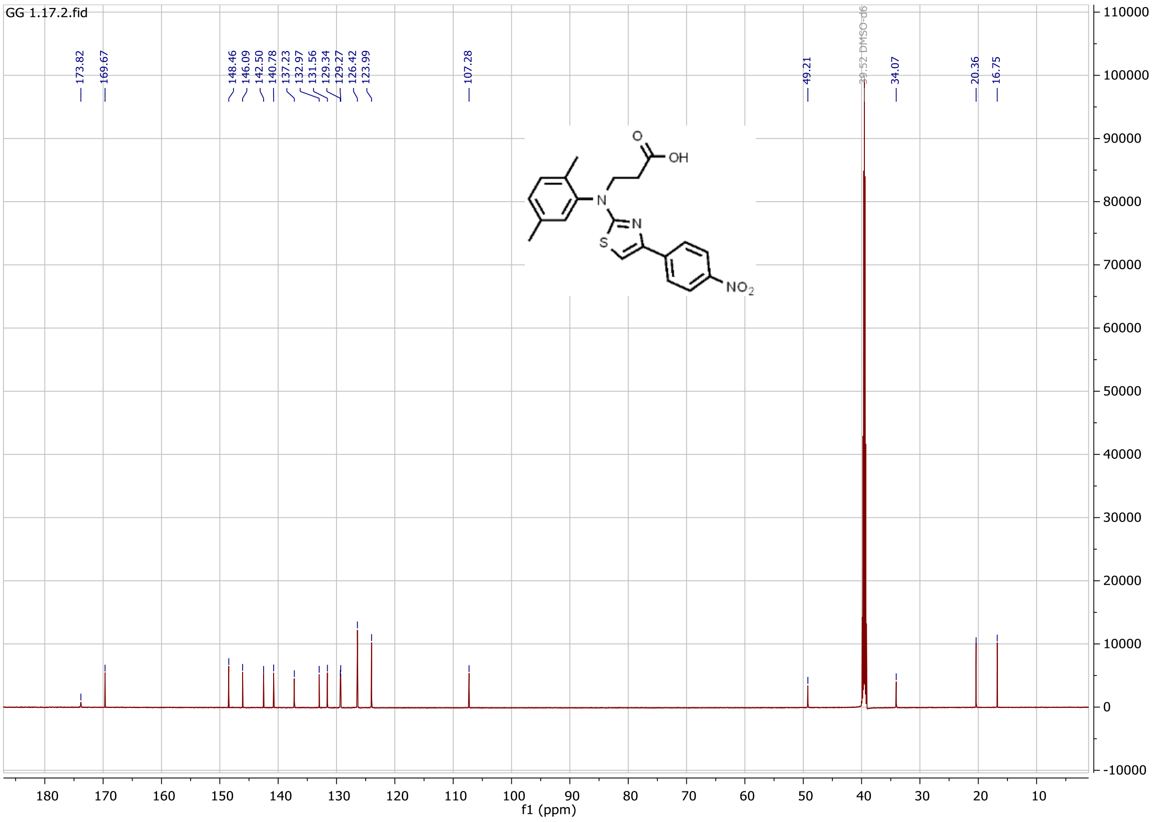

3-*{[4-(3,4-Dichlorophenyl)-1,3-thiazol-2-yl](2,5-dimethylphenyl)amino}propanoic acid* **(3h)**

Figure S19. ^{1}H NMR of compound **3h.** A peak at 3.53 ppm for water is observed in Figure S19, and identified by chemical shifts reported in the publication [1,2].

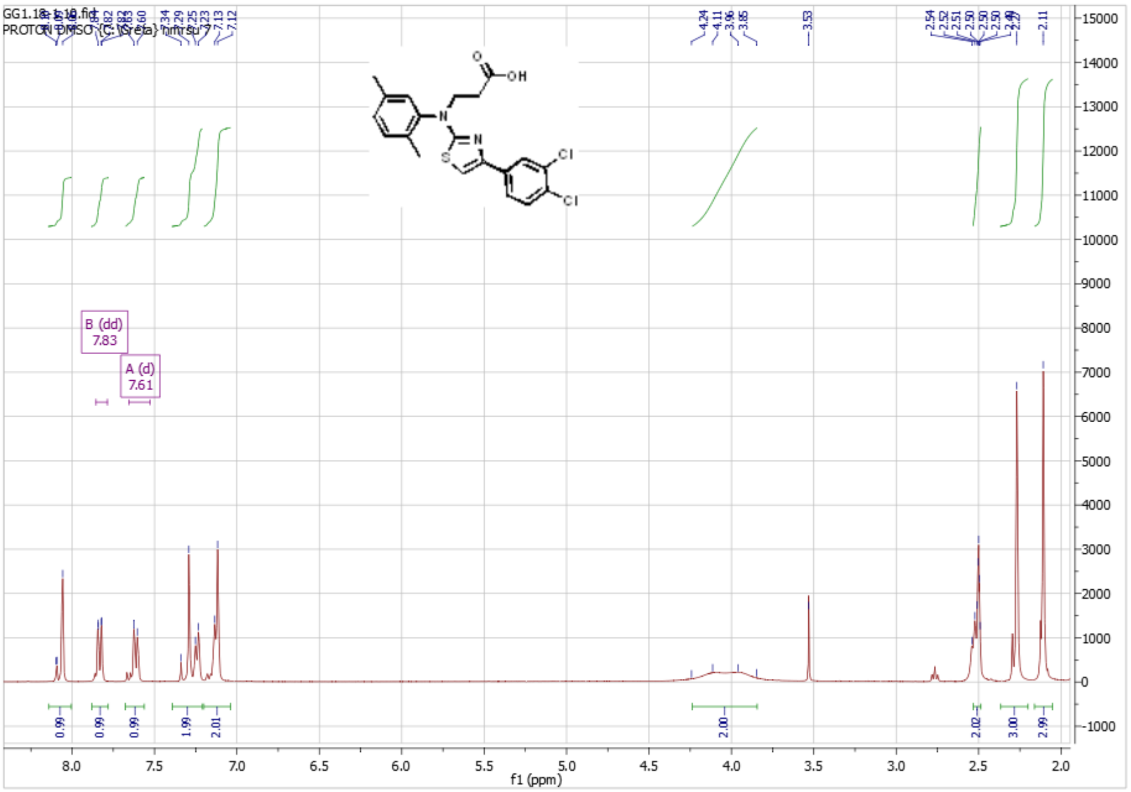

Figure S20. ^{13}C NMR of compound **3h.**

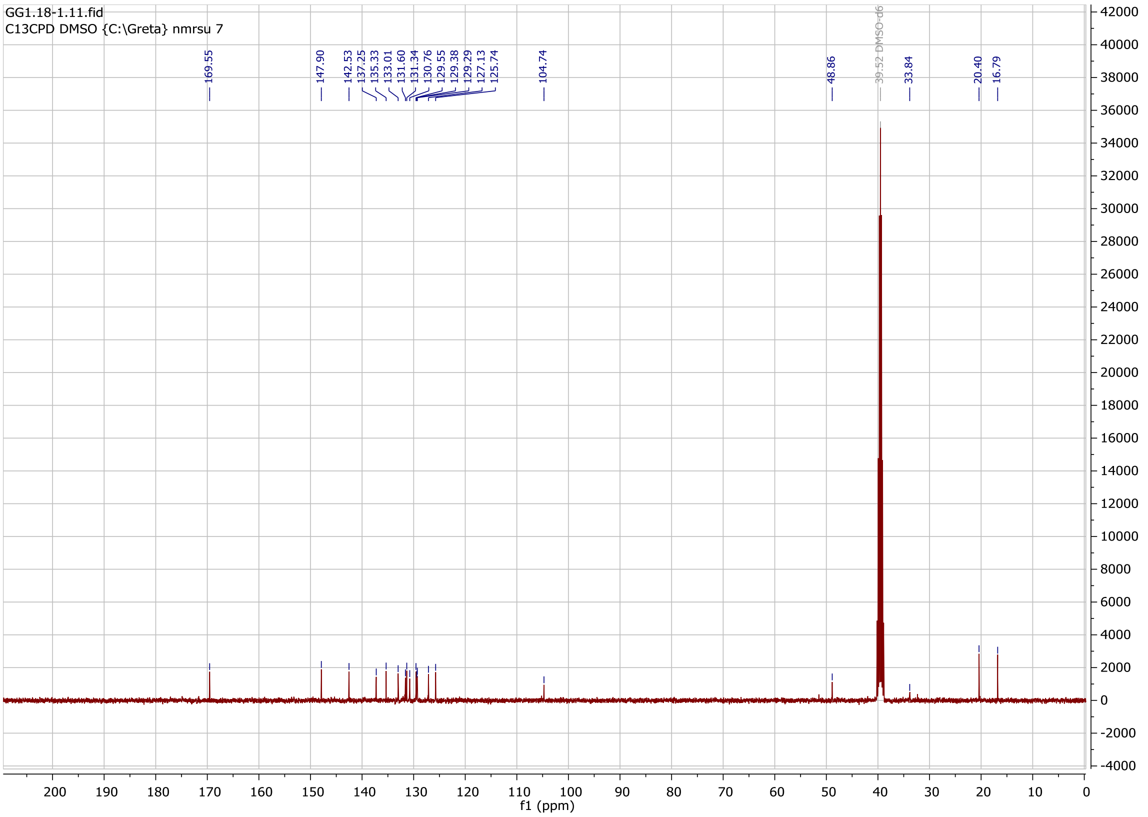

3-{[4-(4-Bromophenyl)-1,3-thiazol-2-yl](2,5-dimethylphenyl)amino}propanoic acid **(3i)**
Figure S21. ^{1}H NMR of compound **3i.**

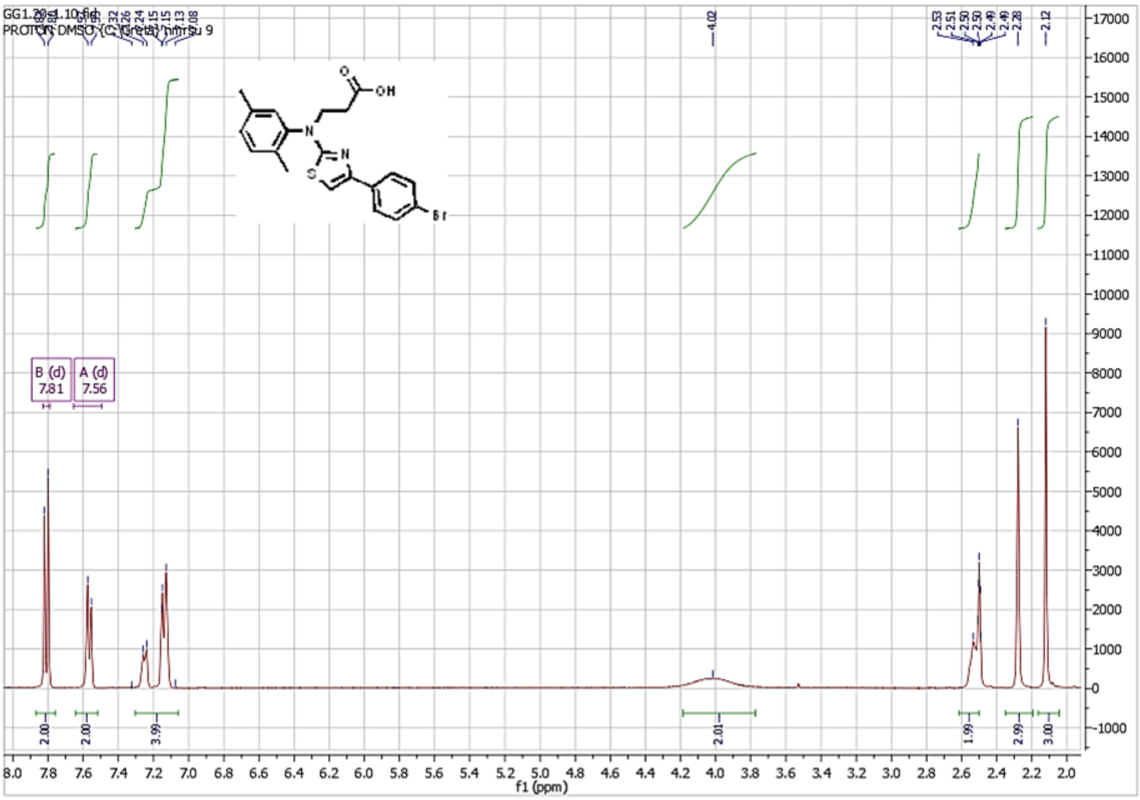

Figure S22. ^{13}C NMR of compound **3i.**

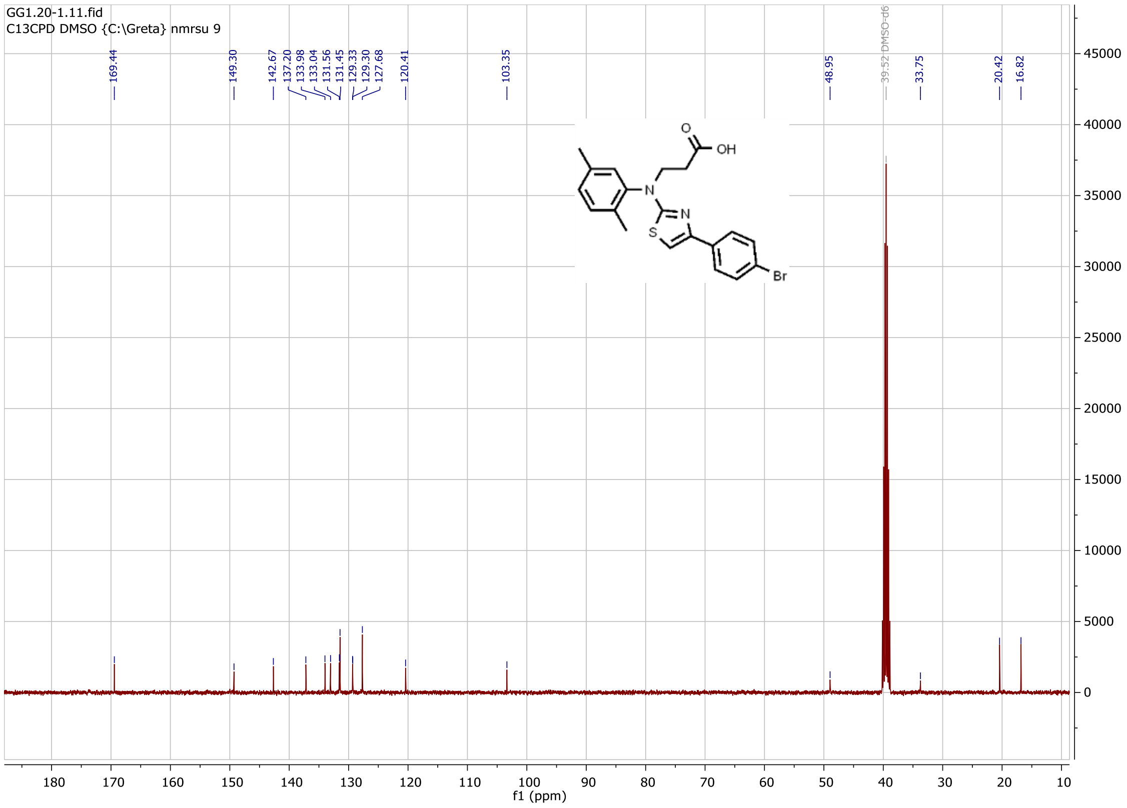

3-*{(2,5-Dimethylphenyl)[4-(naphthalen-2-yl)thiazol-2-yl]amino}propanoic acid* **(3j)**

Figure S23. ^{1}H NMR of compound **3j.**

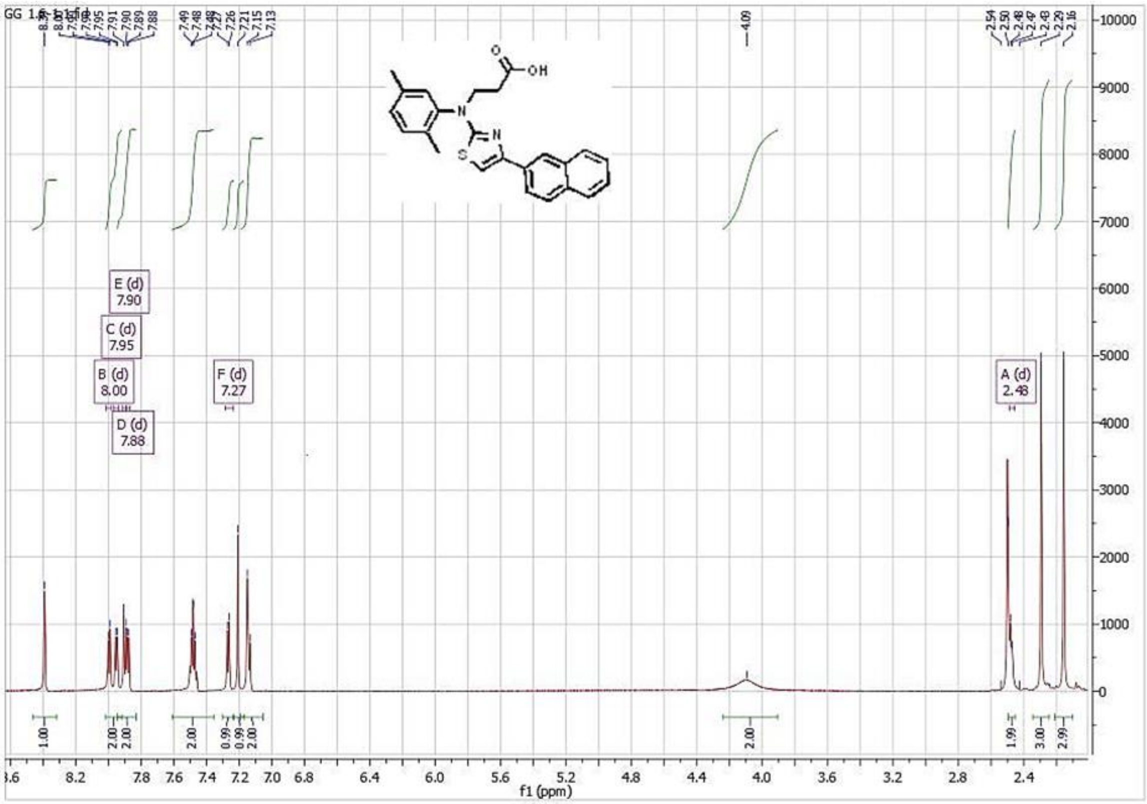

Figure S24. ^{13}C NMR of compound **3j.**

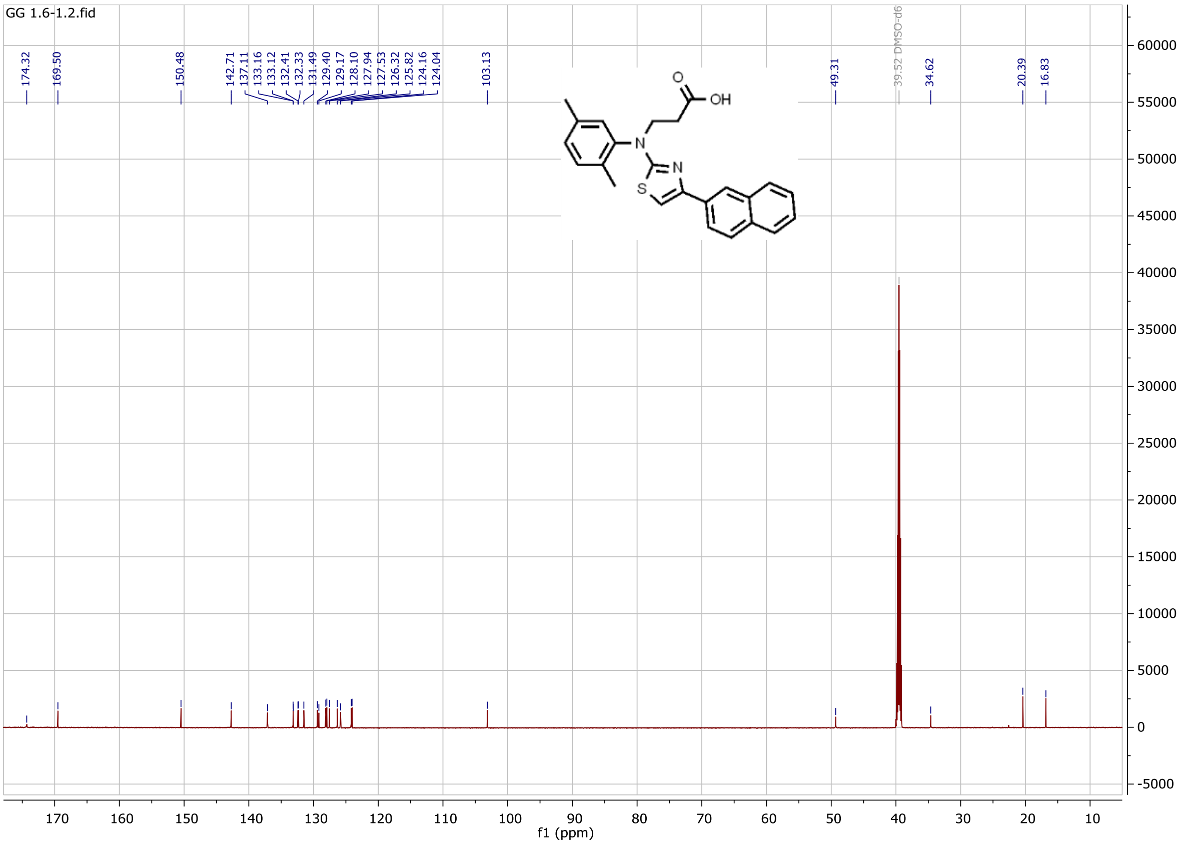

3-{(2,5-Dimethylphenyl)[4-(2-oxo-2H-chromen-3-yl)thiazol-2-yl]amino}propanoic acid **(3k)**

Figure S25. ^{1}H NMR of compound **3k.**

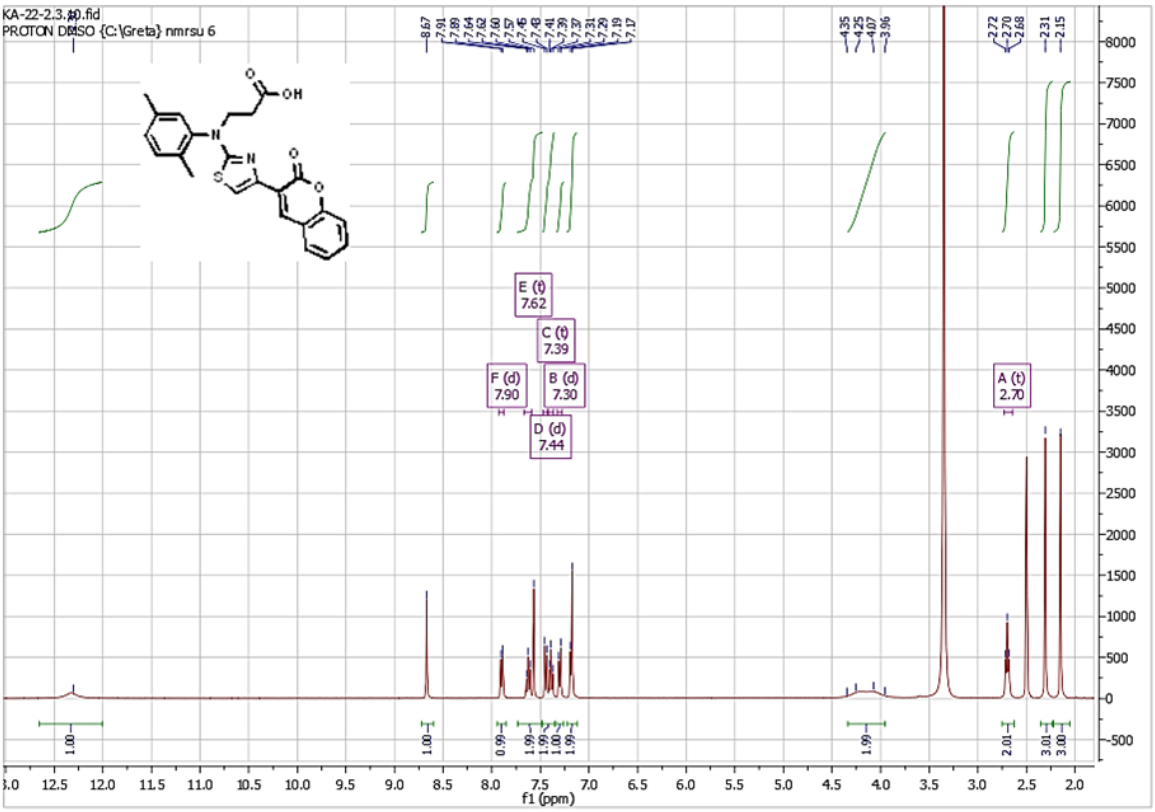

Figure S26. ^{13}C NMR of compound **3k.**

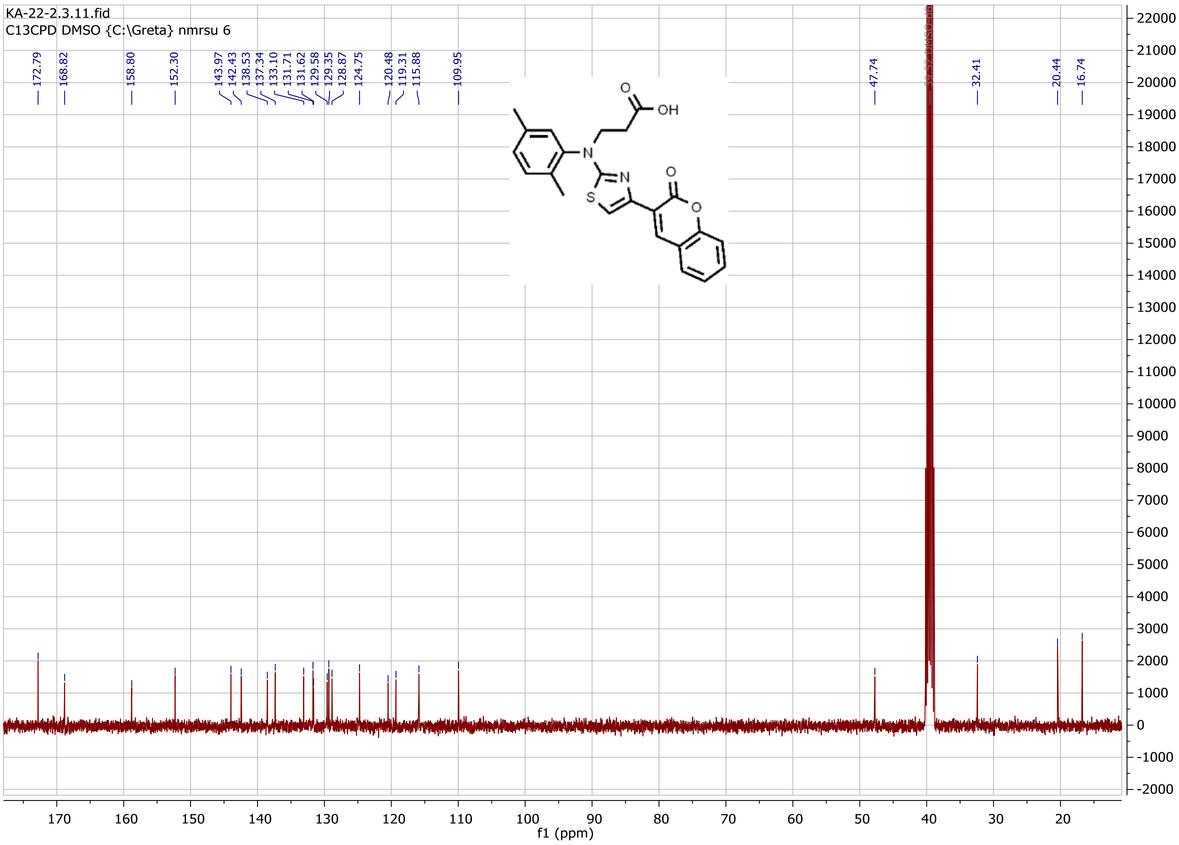

General synthesis of compounds 4i-k

1-[4-(4-Bromophenyl)-1,3-thiazol-2-yl]-5,8-dimethyl-2,3-dihydroquinolin-4(1H)-one **(4i)**

Figure S27. ^{1}H NMR of compound **4i.** A peak at 3.33 ppm for water is observed in Figure S27 and identified by chemical shifts reported in the publication [1,2].

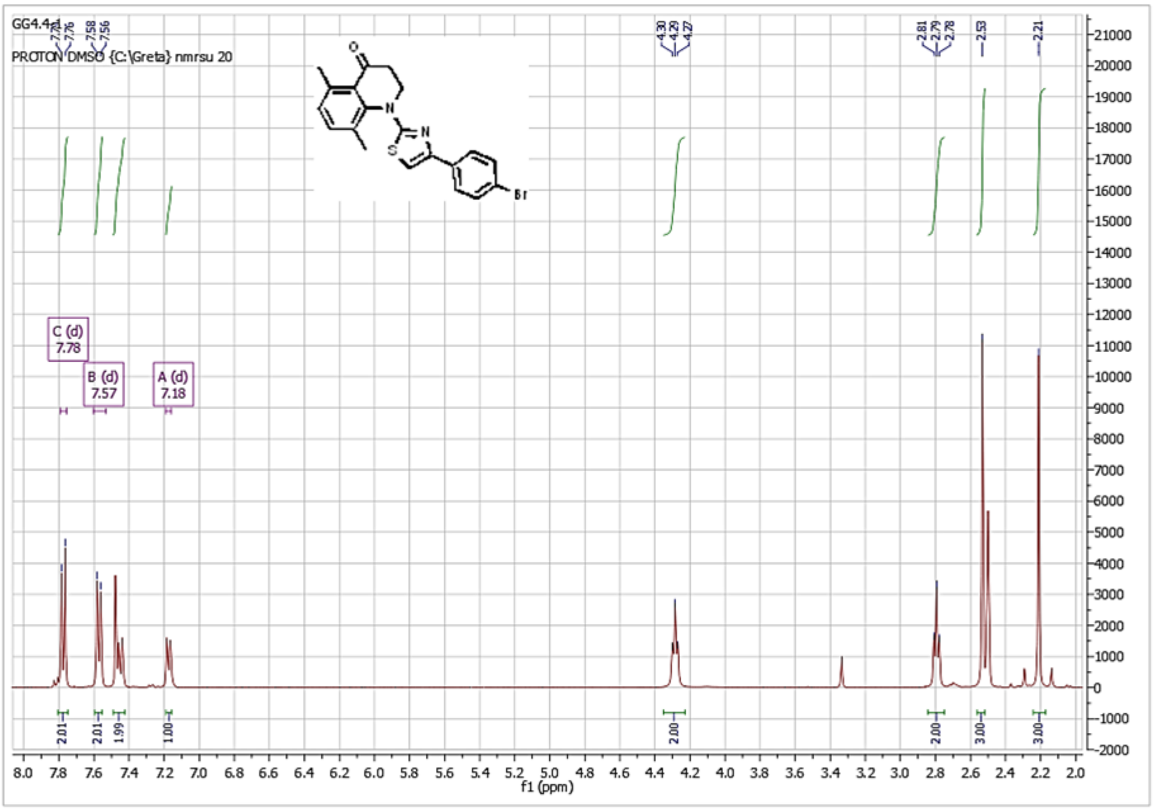

Figure S28. ^{13}C NMR of compound **4i.**

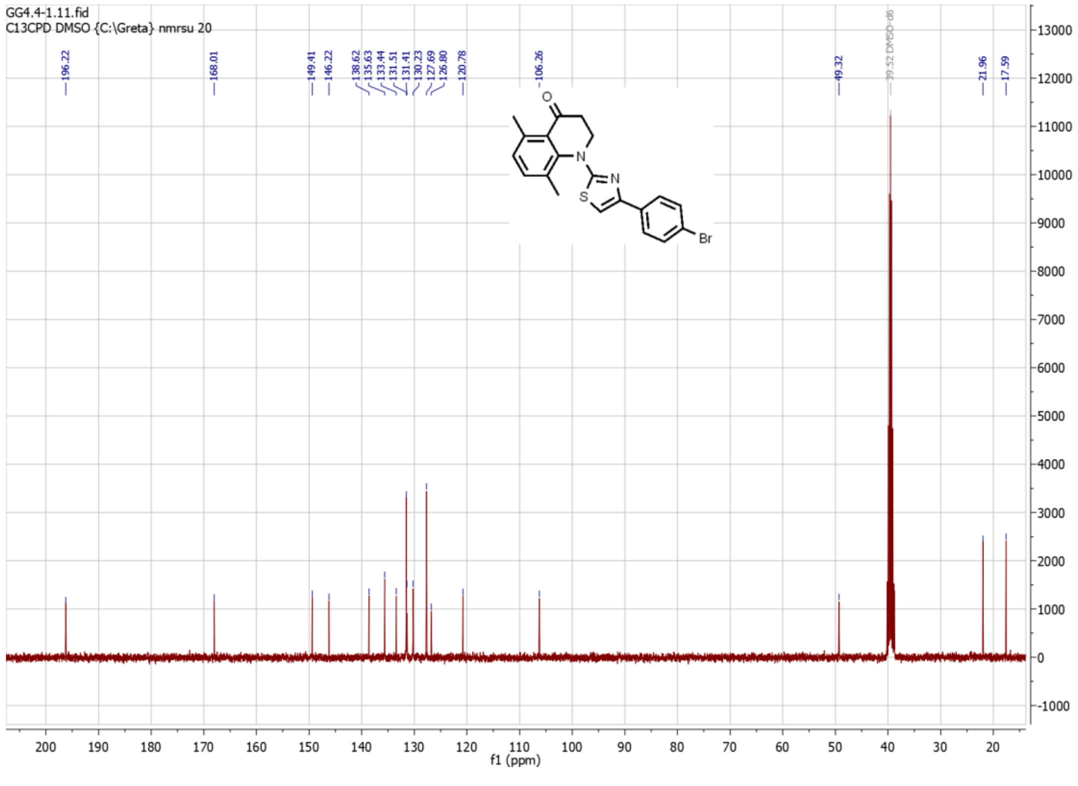

5,8-Dimethyl-1-[4-(naphthalen-2-yl)-1,3-thiazol-2-yl]-2,3-dihydroquinolin-4(1H)-one **(4j)**
Figure S29. ^{1}H NMR of compound **4j**

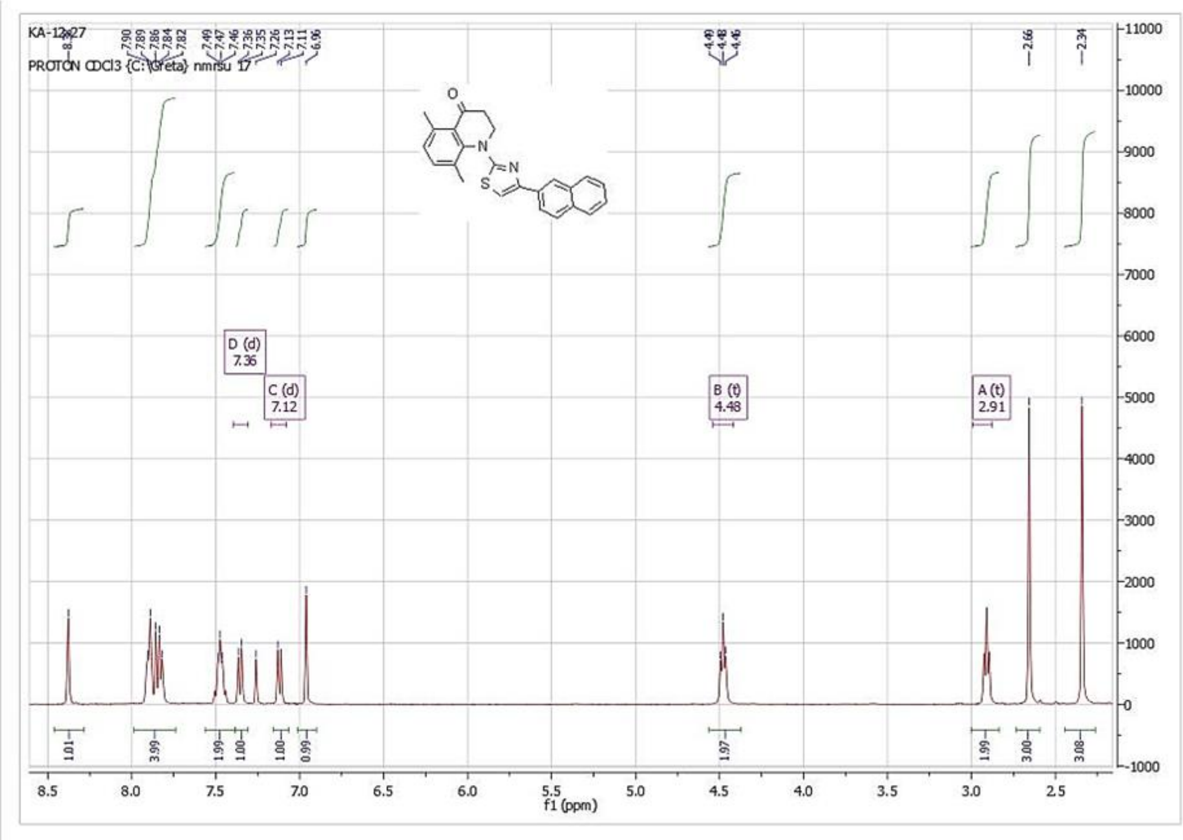

Figure S30. ^{13}C NMR of compound **4j.**

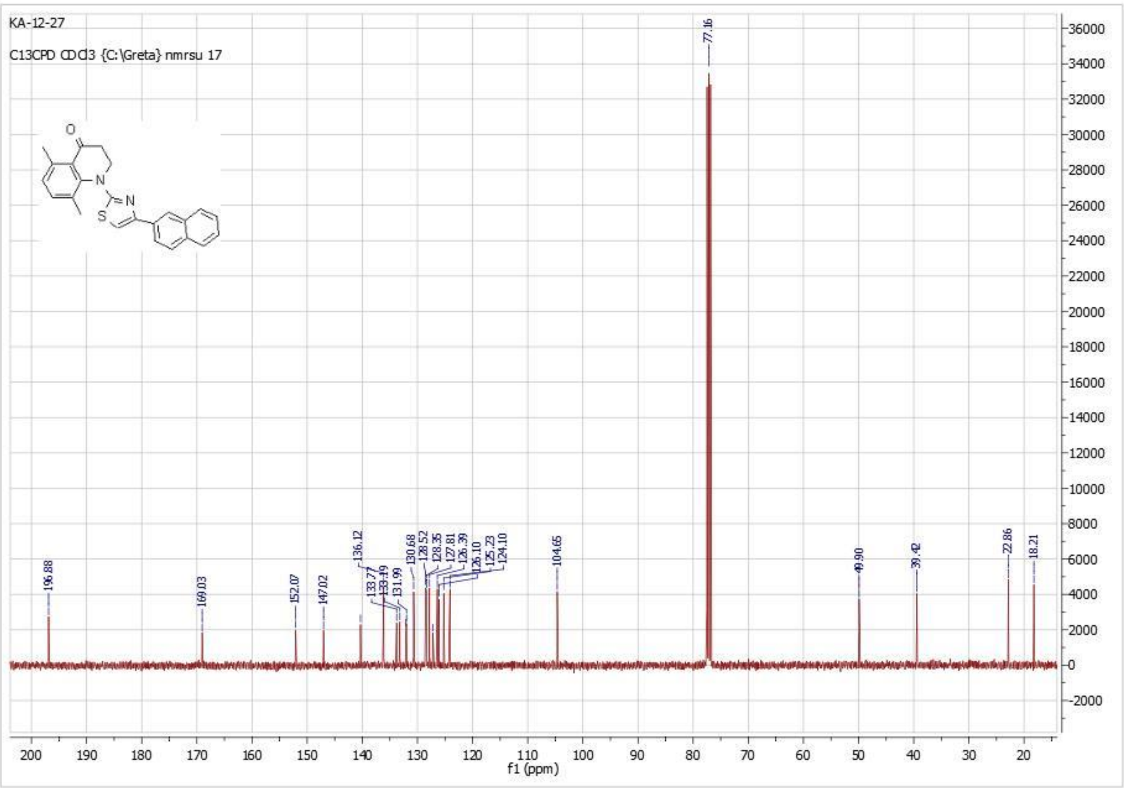

5,8-Dimethyl-1-[4-(2-oxo-2H-1-benzopyran-3-yl)-1,3-thiazol-2-yl]-2,3-dihydroquinolin-4(1H)-one **(4k)**

Figure S31. ^{1}H NMR of compound **4k.** A broad peak at 3.33 ppm for water is observed in Figure S31 and identified by chemical shifts reported in the publication [1,2].

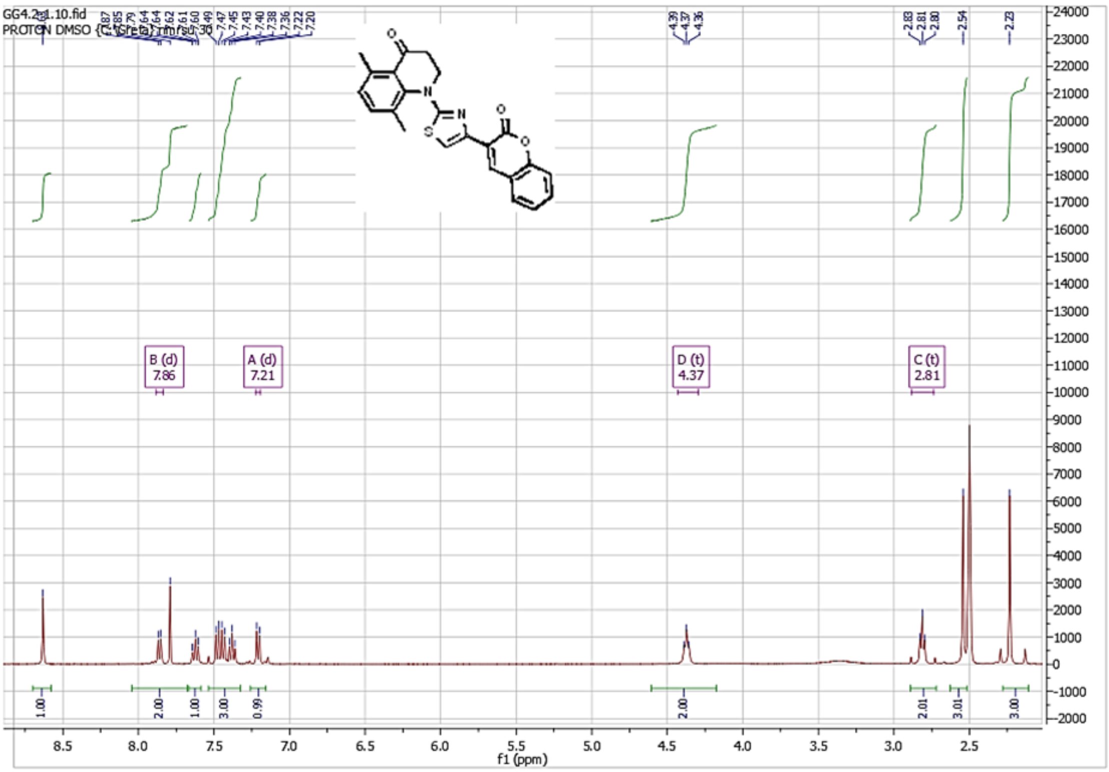

Figure S32. ^{13}C NMR of compound **4k.**

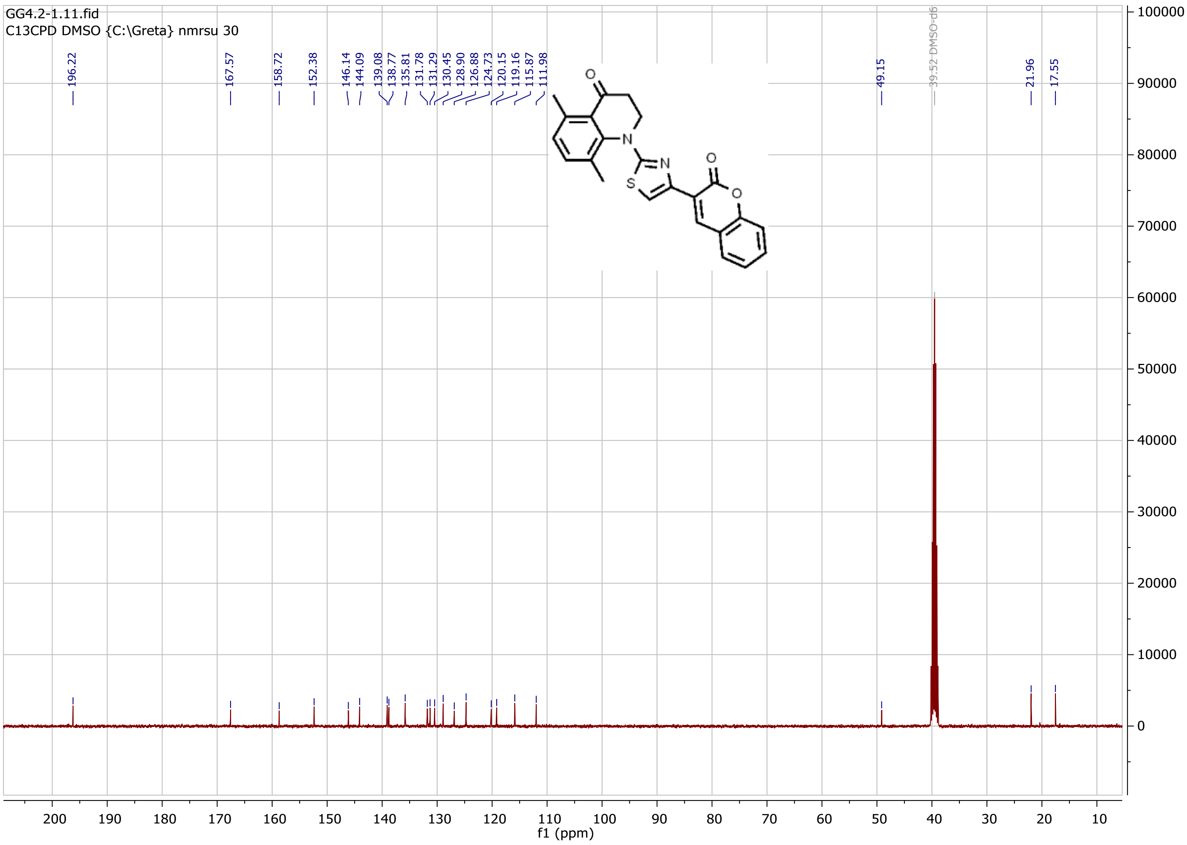

3-[(5-Acetyl-4-methyl-1,3-thiazol-2-yl)(2,5-dimethylphenyl)amino]propanoic acid **(5)**

Figure S33. ^{1}H NMR of compound **5.**

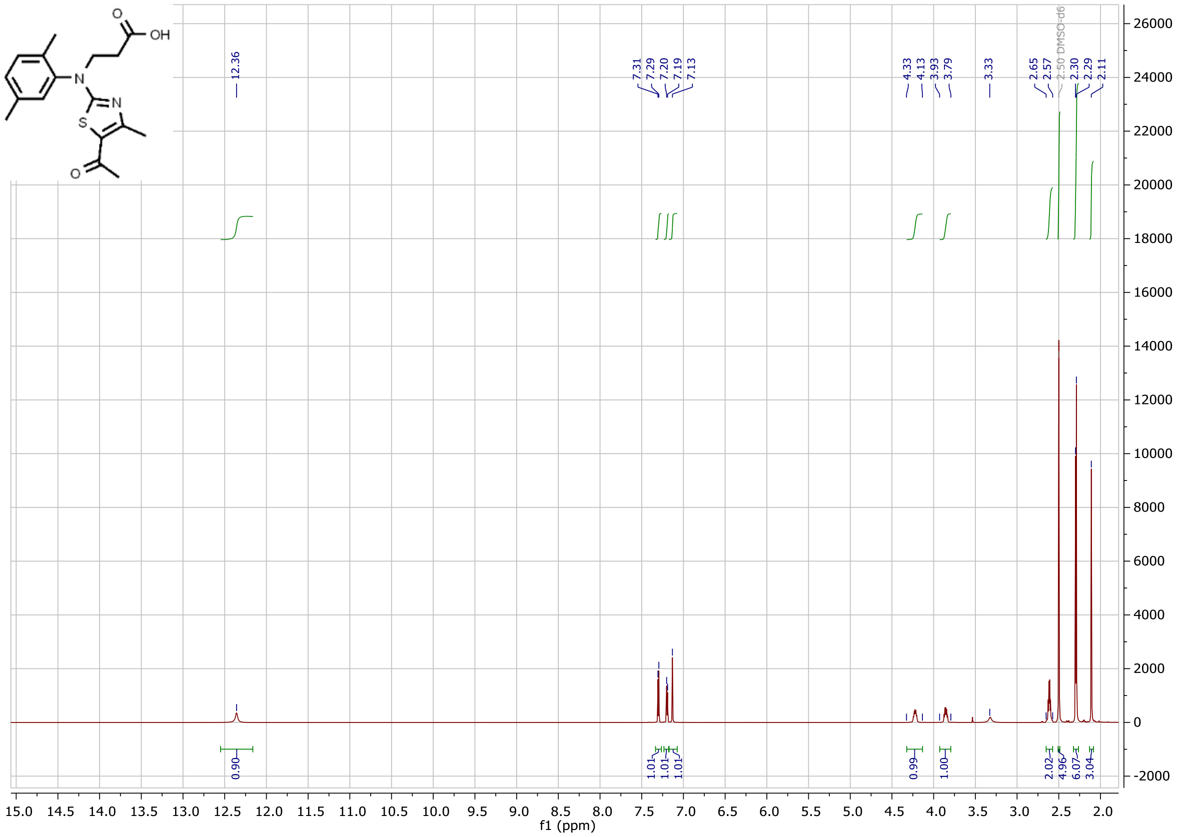

Figure S34. ^{13}C NMR of compound **5.**

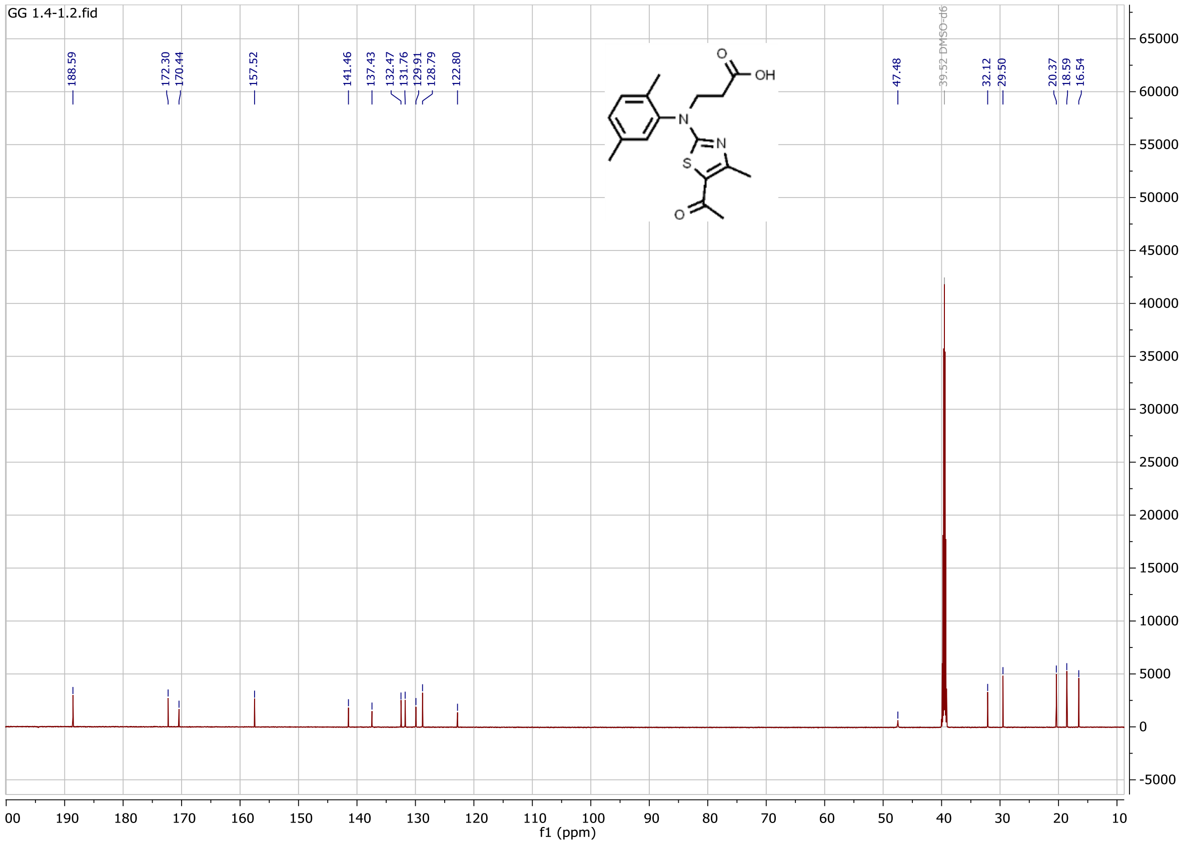

3-[(2,5-Dimethylphenyl)([1,3]thiazolo[4,5-b]quinoxalin-2-yl)amino]propanoic acid (**6**)

Figure S35. ^{1}H NMR of compound **6.** A broad peak at 3.33 ppm for water is observed in Figure S35 and identified by chemical shifts reported in the publication [1,2].

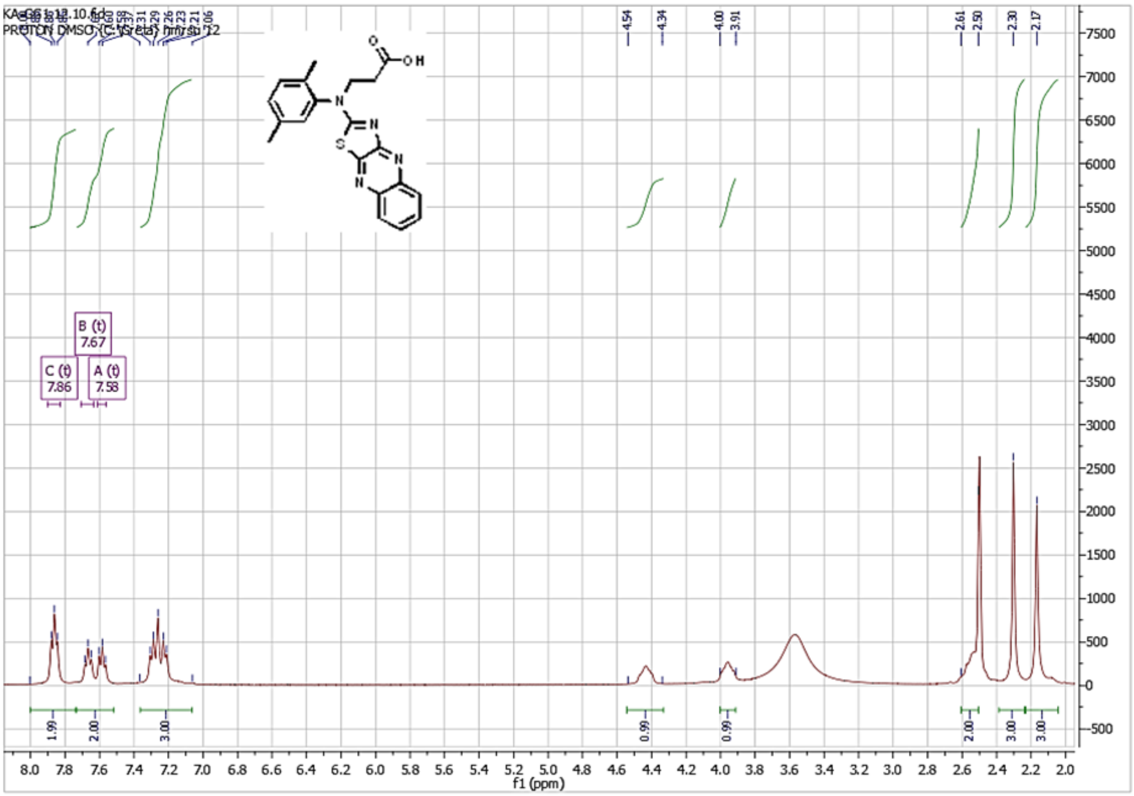

Figure S36. ^{13}C NMR of compound **6.**

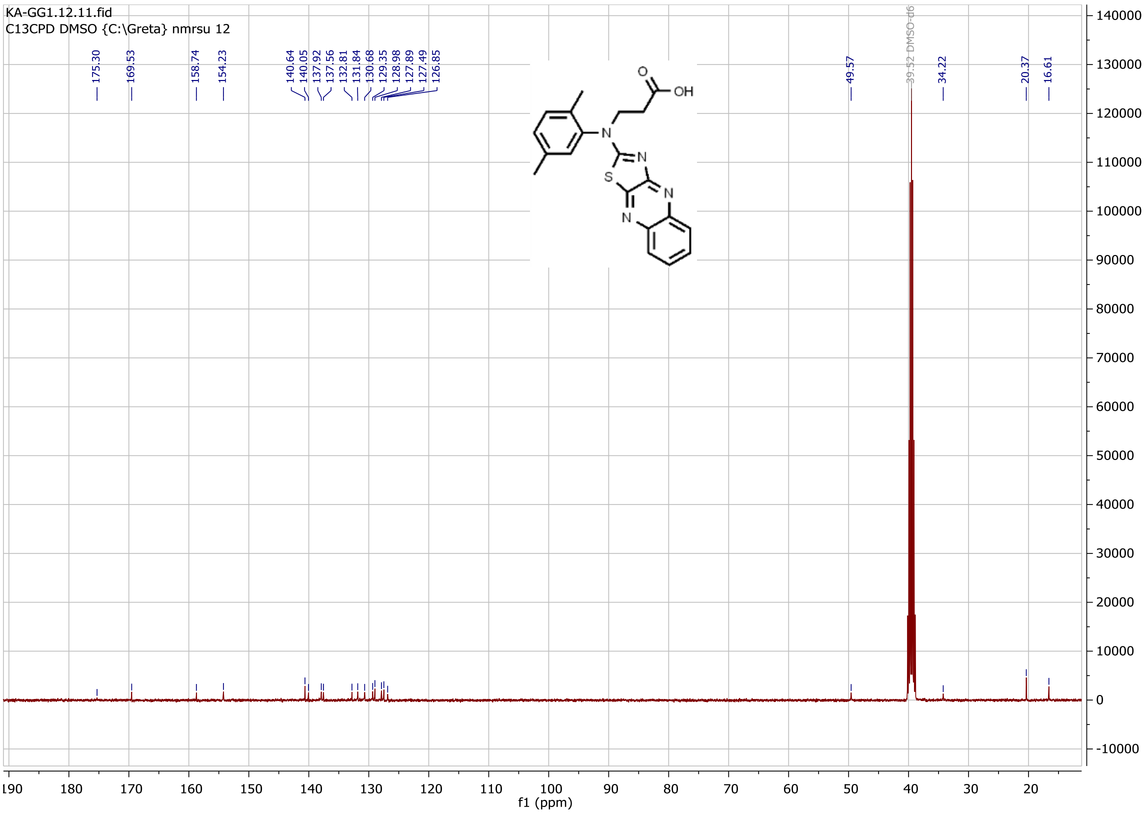

3-[(2,5-Dimethylphenyl)(4,9-dioxo-4,9-dihydronaphtho[2,3-d][1,3]thiazol-2-yl)amino]propanoic acid **(7)**
Figure S37. ^{1}H NMR of compound **7.**

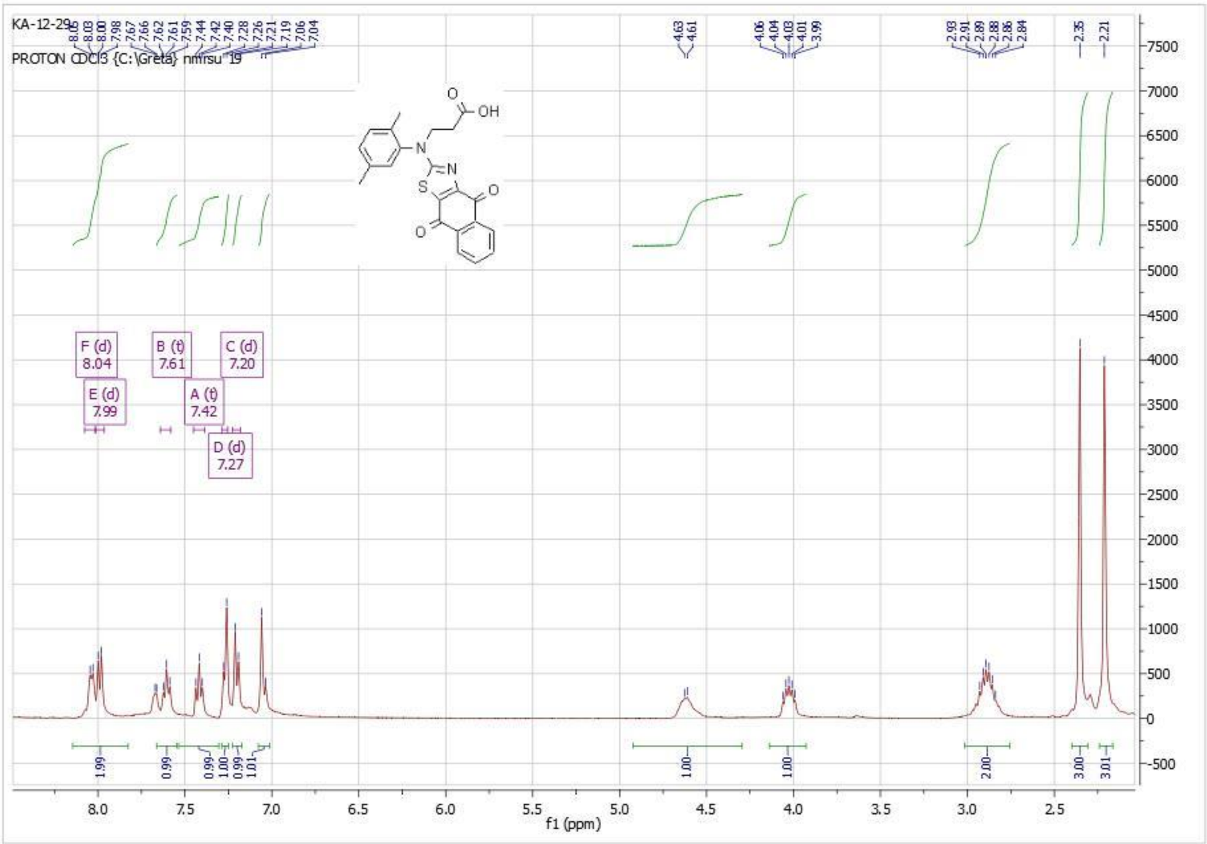

Figure S38. ^{13}C NMR of compound **7.**

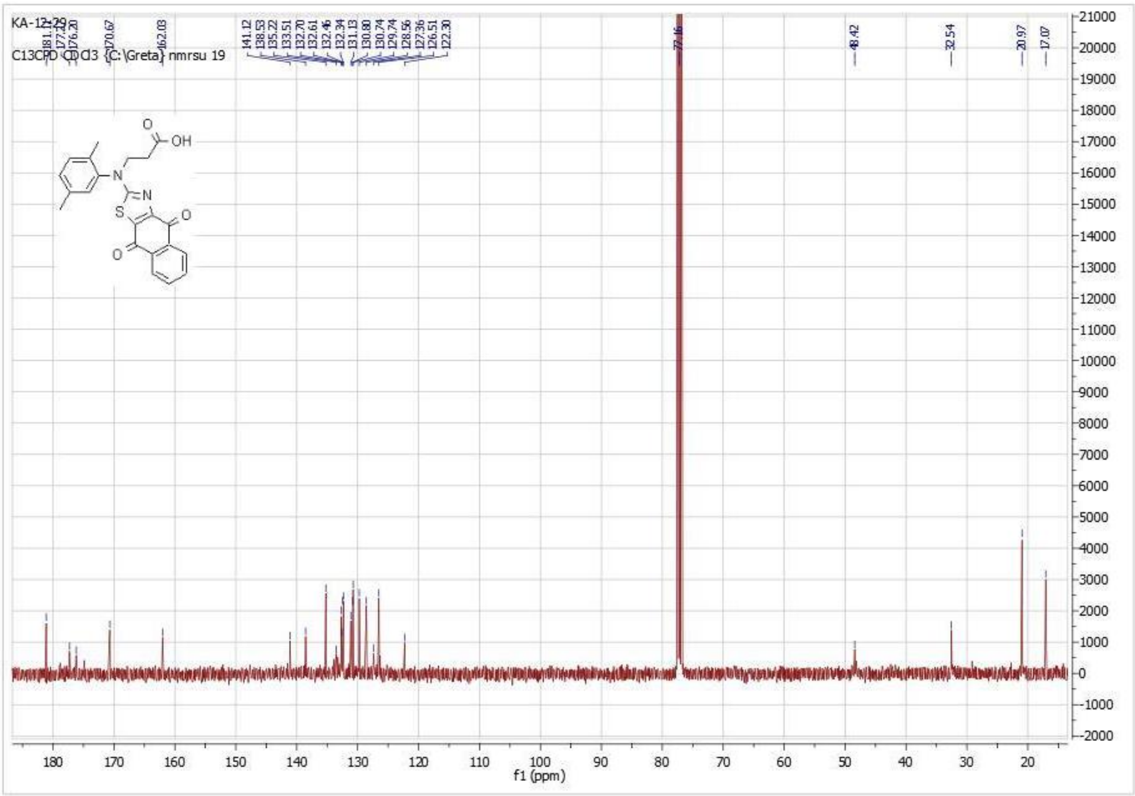

Methyl 3-{[4-(4-chlorophenyl)-1,3-thiazol-2-yl](2,5-dimethylphenyl)amino}propanoate **(8f)**

Figure S39. ^{1}H NMR of compound **8f.** A peak at 3.33 ppm for water is observed in Figure S5 and identified by chemical shifts reported in the publication [1,2].

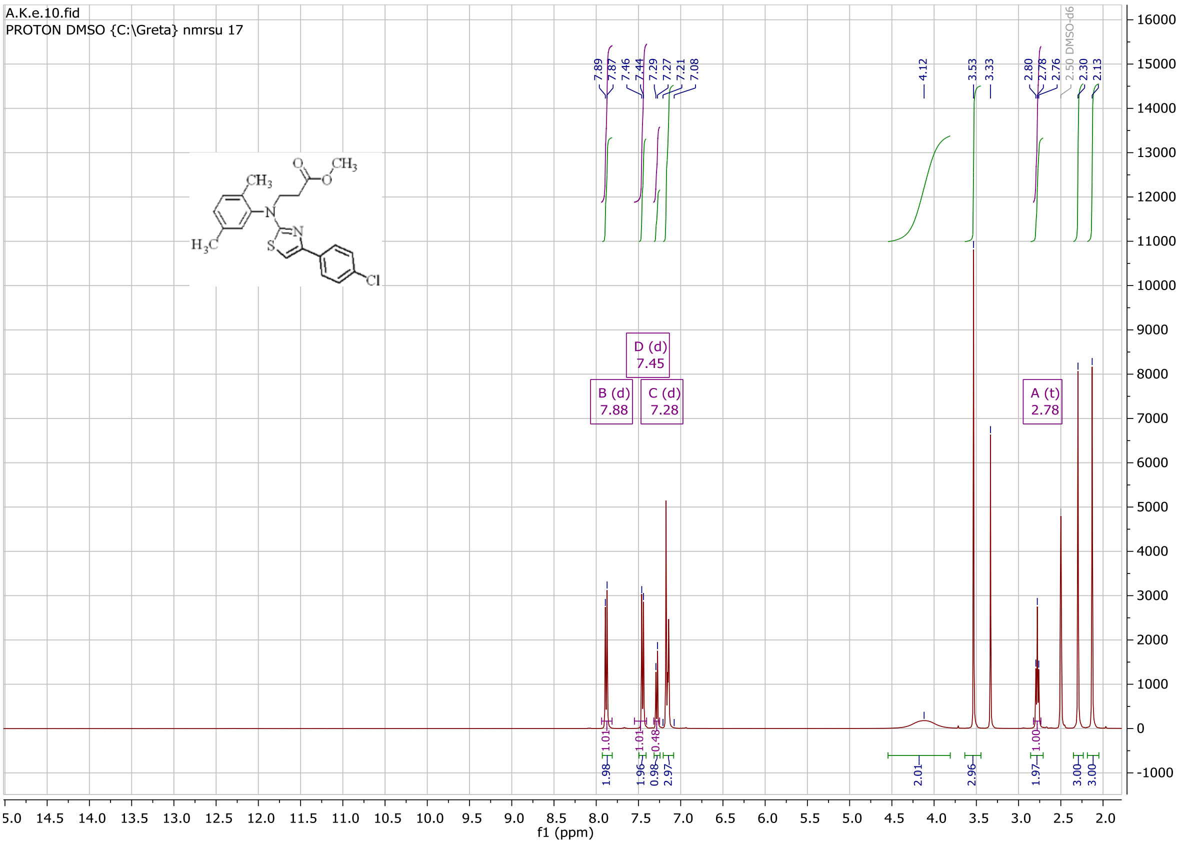

Figure S40. ^{13}C NMR of compound **8f.**

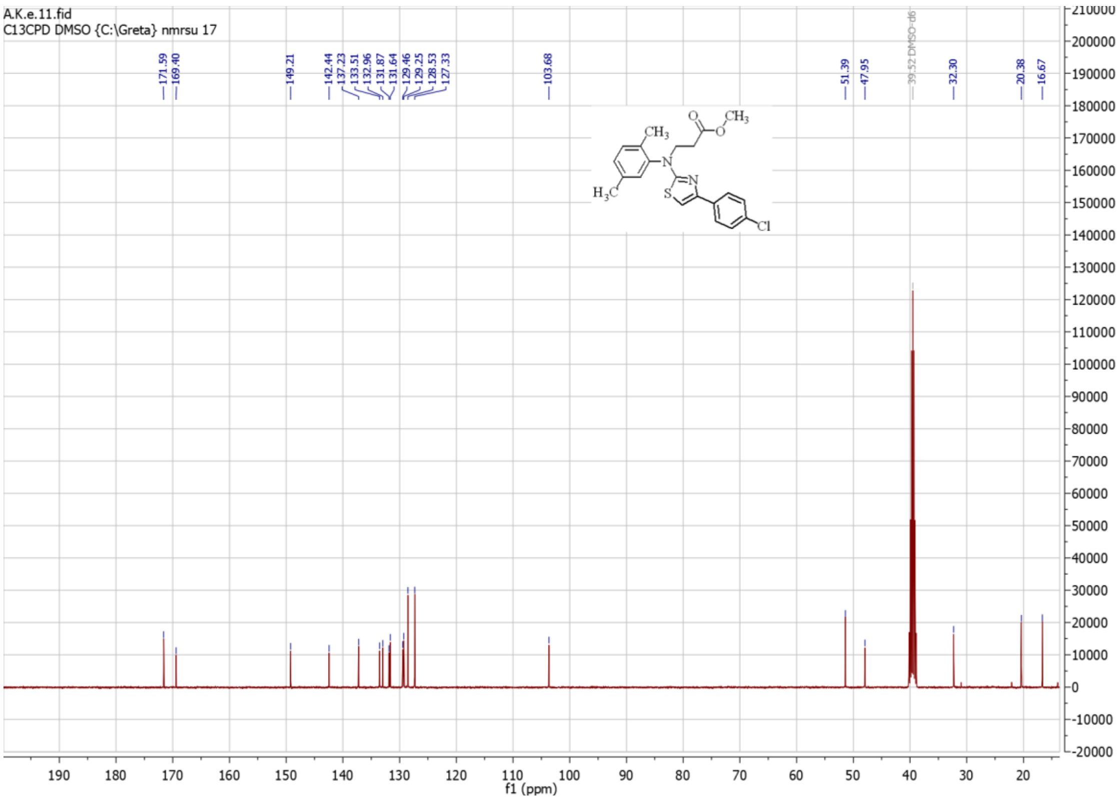

3-{[4-(4-Chlorophenyl)-1,3-thiazol-2-yl](2,5-dimethylphenyl)amino}propanehydrazide **(9f)**

Figure S41. ^{1}H NMR of compound **9f.** A peak at 3.33 ppm for water is observed in Figure S41 and identified by chemical shifts reported in the publication [1,2].

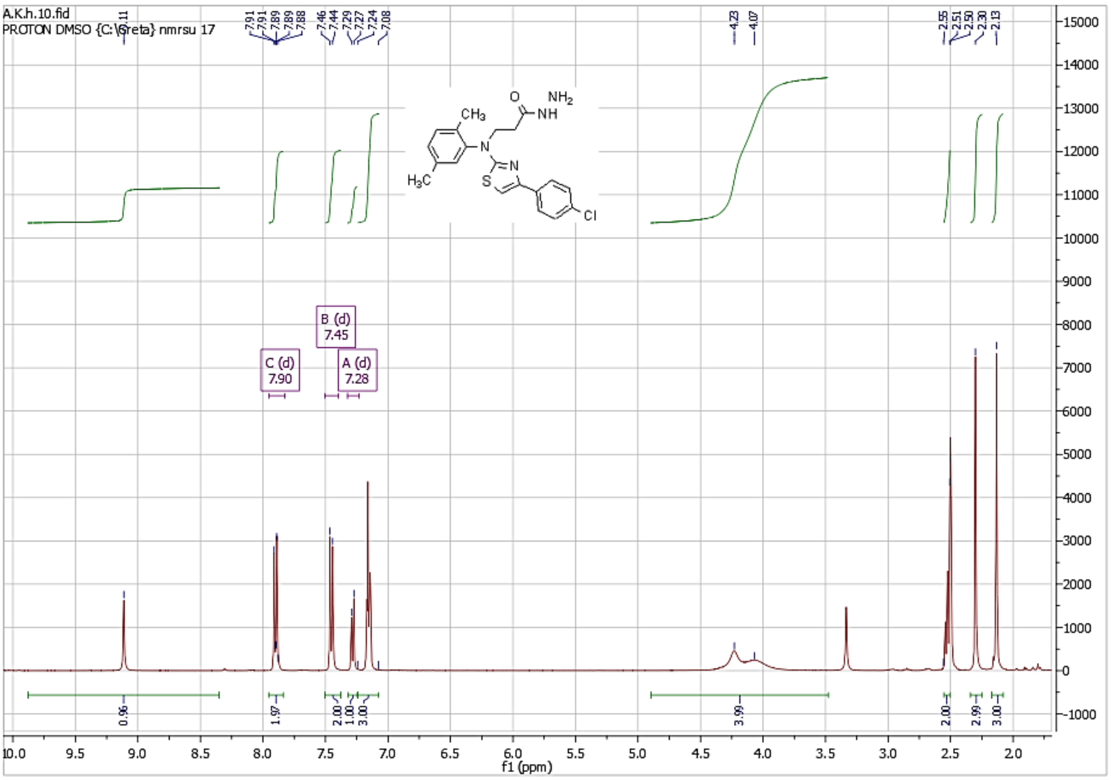

Figure S42. ^{13}C NMR of compound **9f.**

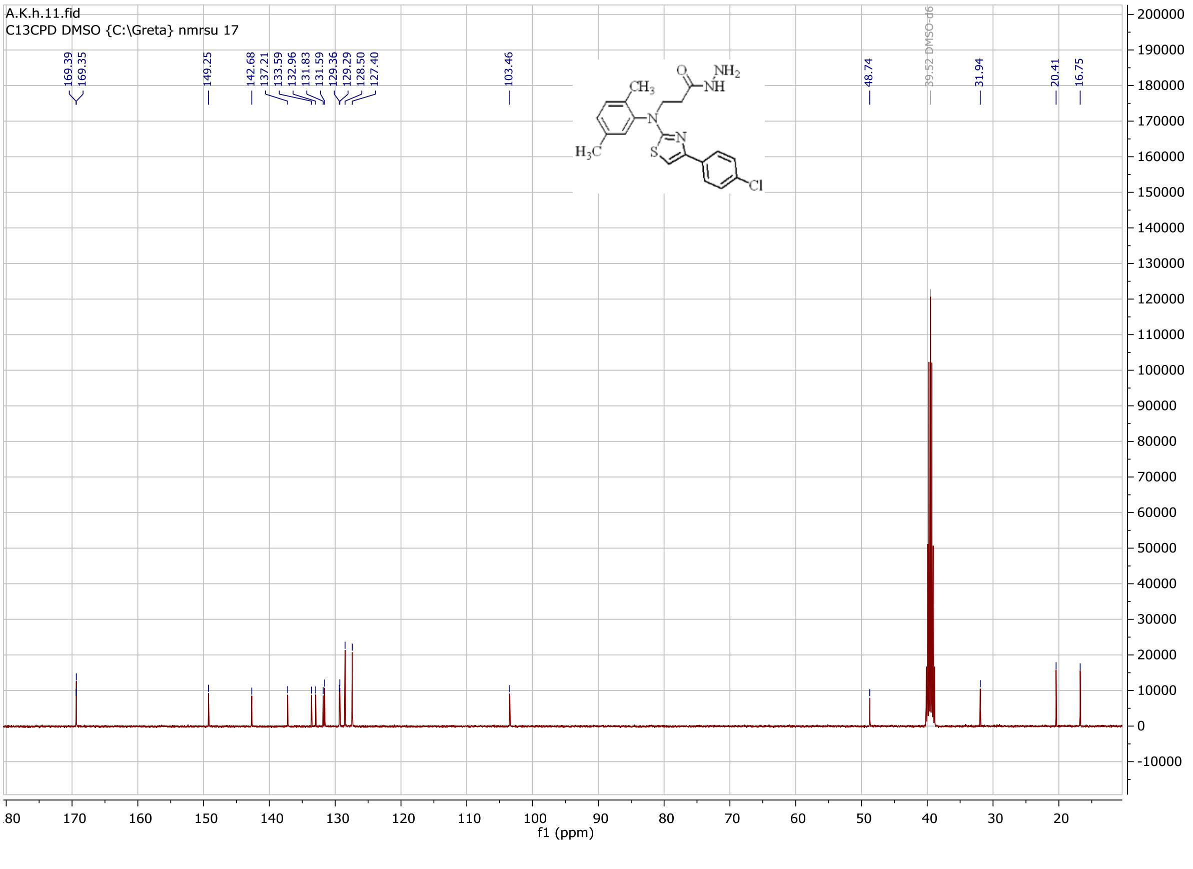

General synthesis of compounds 10–12f

(Z/E)-3-{[4-(4-Chlorophenyl)thiazol-2-yl](2,5-dimethylphenyl)amino}-N'-[(5-nitrothiophen-2-yl)methylene]propanehydrazide **(10f)**

Figure S43. ^{1}H NMR of compound **10f.** The peaks for 1,4-dioxane at 3.53 ppm in figure S43 and 66.35 ppm in figure S44 is noticed and identified by reference to chemical shifts described in the publication [1].

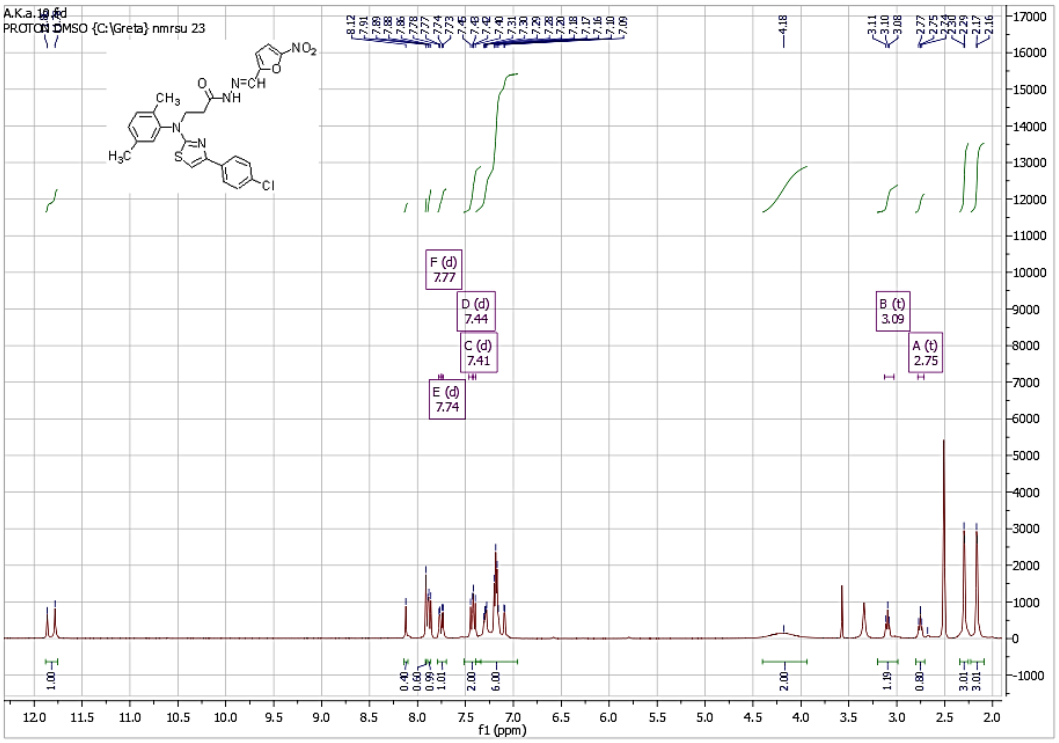

Figure S44. ^{13}C NMR of compound **10f.**

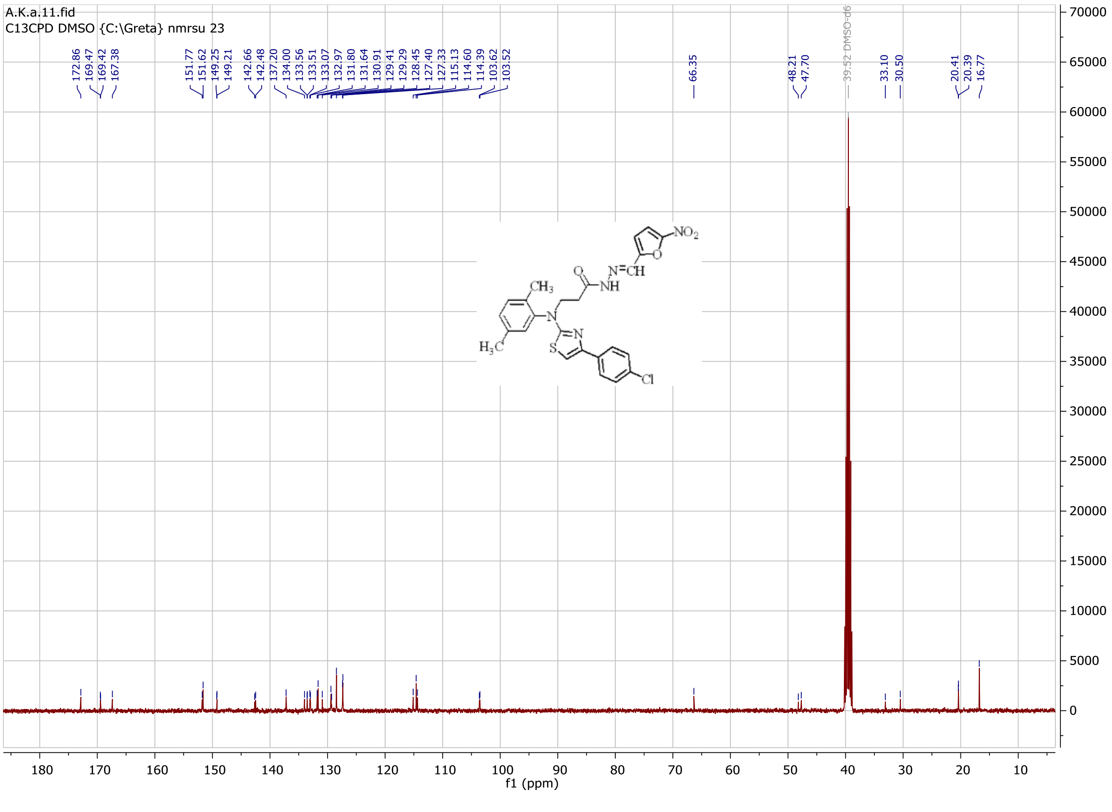

(Z/E)-3-((4-(4-Chlorophenyl)thiazol-2-yl)(2,5-dimethylphenyl)amino)-N'-((5-nitrofuran-2-yl)methylene)propanehydrazide **(11f)**

Figure S45. ^{1}H NMR of compound **11f.** A broad peak at 3.33 ppm for water is observed in Figure S45 and identified by chemical shifts reported in the publication [1,2].

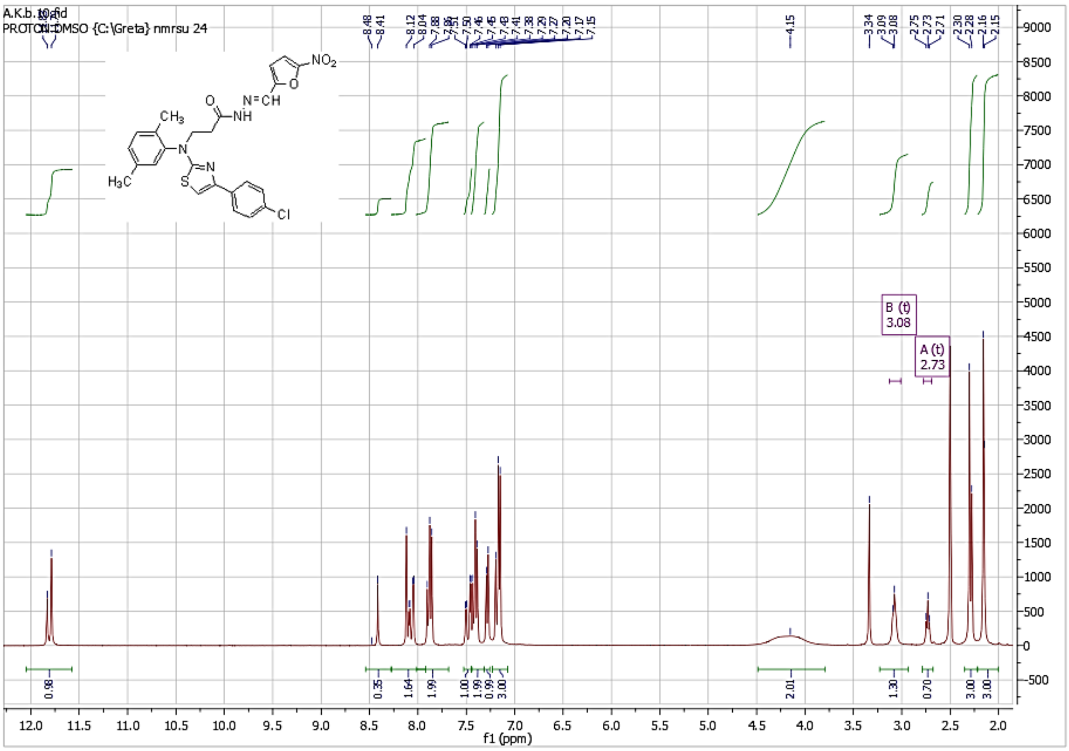

Figure S46. ^{13}C NMR of compound **11f.**

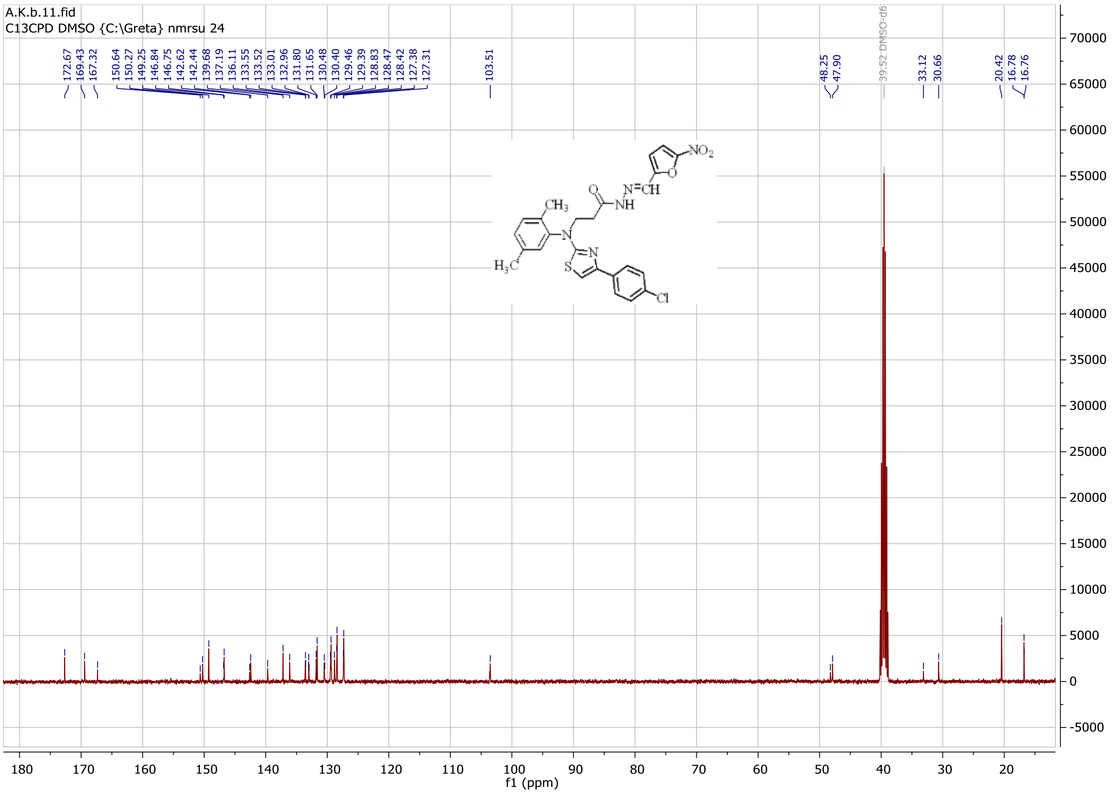

(Z/E)-N'-((1H-Indol-3-yl)methylene)-3-((4-(4-chlorophenyl)thiazol-2-yl)(2,5-dimethylphenyl)amino)propanehydrazide **(12f)**

Figure S47. ^{1}H NMR of compound **12f.** A broad peak at 3.33 ppm for water is observed in Figure S47 and identified by chemical shifts reported in the publication [1,2].

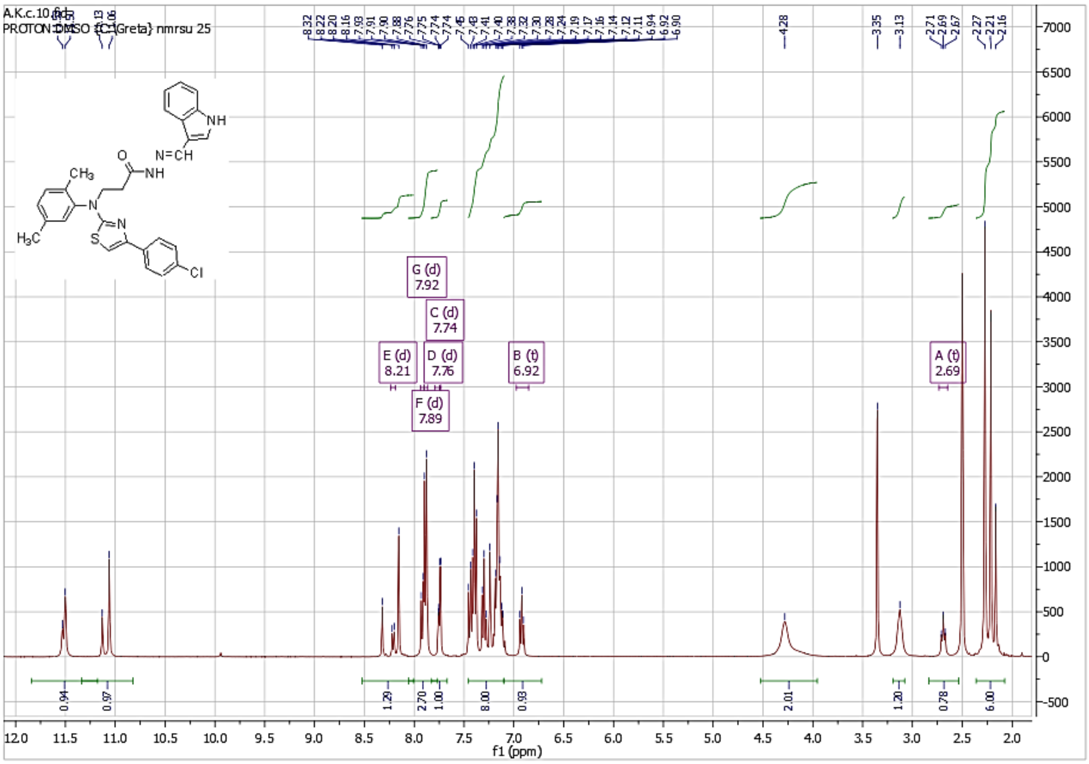

Figure S48. ^{13}C NMR of compound **12f.**

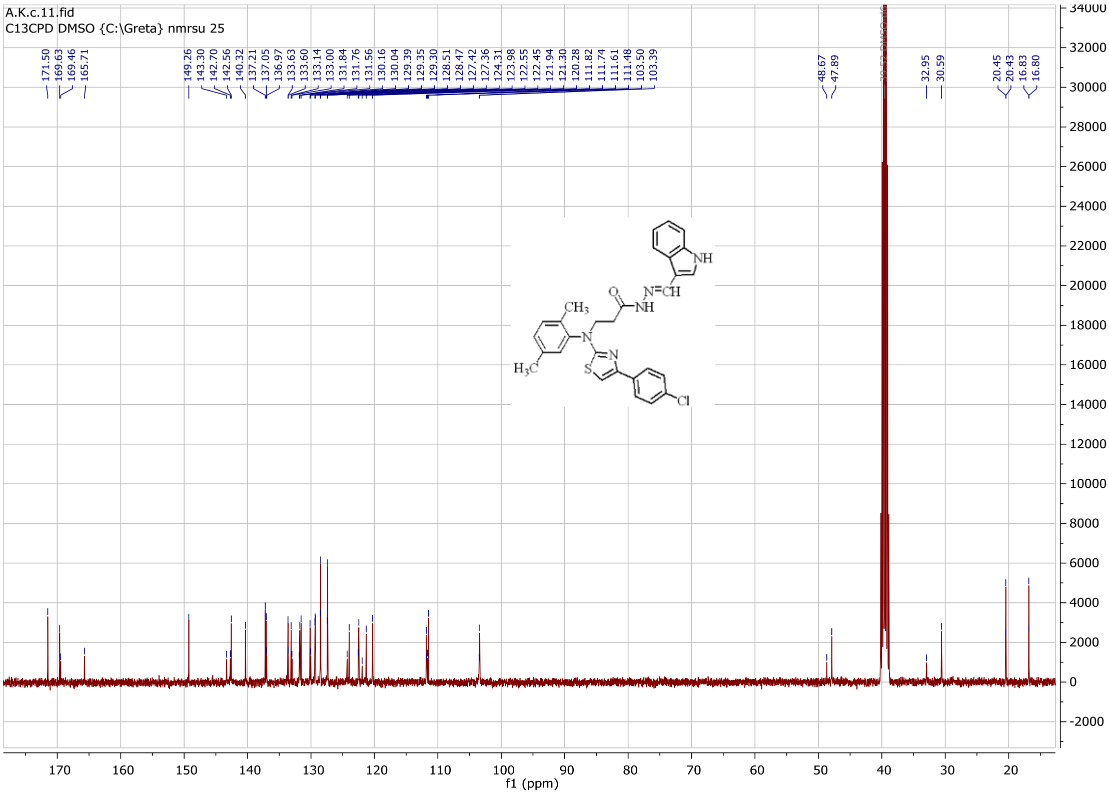

3-{[4-(4-Chlorophenyl)thiazol-2-yl](2,5-dimethylphenyl)amino}-N'-cyclopentylidenepropanehydrazide (**13f**)

Figure S49. ^{1}H NMR of compound **13f.** A broad peak at 3.33 ppm for water is observed in Figure S49 and identified by chemical shifts reported in the publication [1,2]. The peaks for 1,4-dioxane at 3.53 ppm in figure S49 and 66.35 ppm in figure S50 is noticed and identified by reference to chemical shifts described in the publication [1].

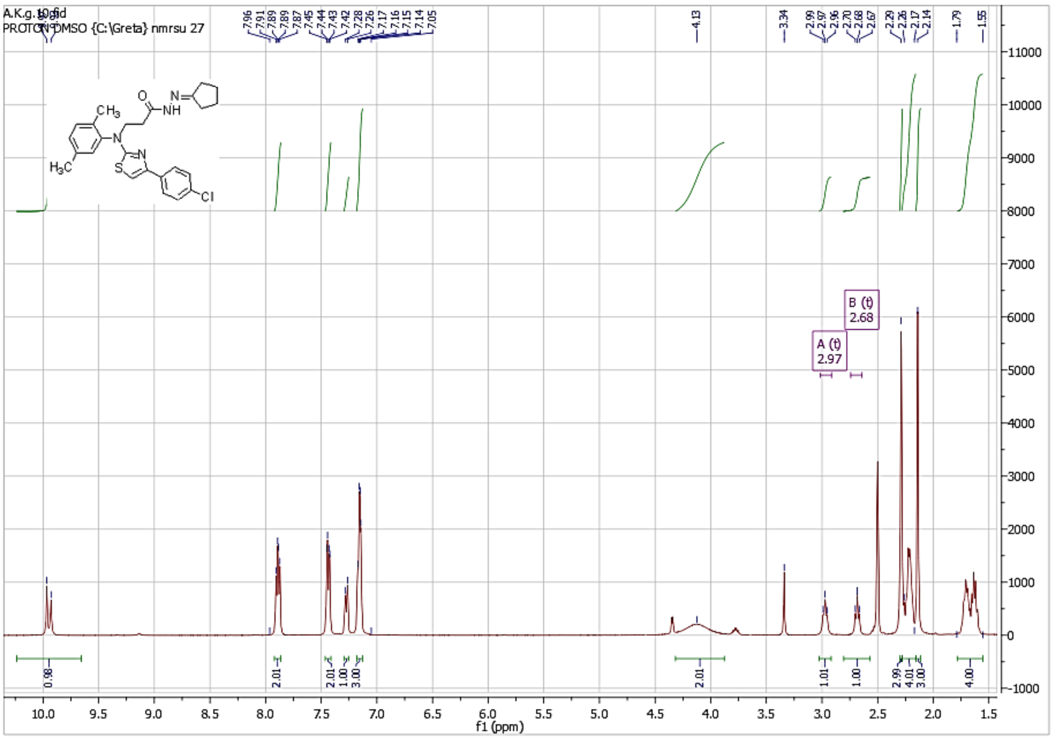

Figure S50. ^{13}C NMR of compound **13f.**

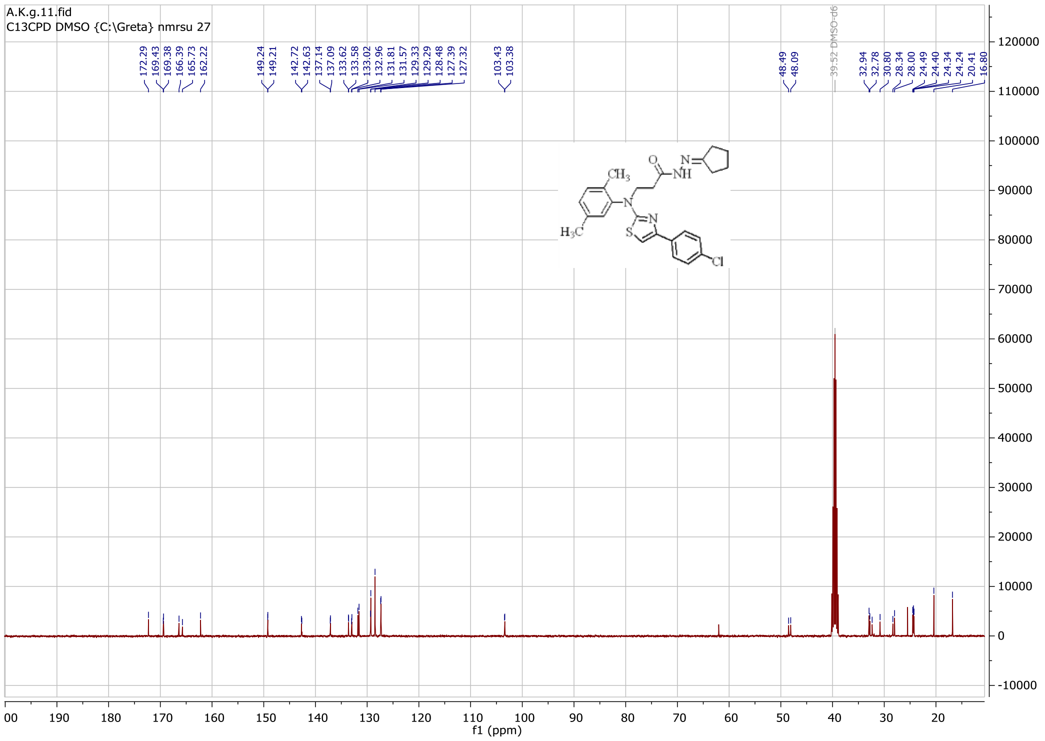

General synthesis of compounds 14f and 15f

(Z/E)-N'-(Butan-2-ylidene)-3-{[4-(4-chlorophenyl)thiazol-2-yl](2,5-dimethylphenyl)amino}propanehydrazide **(14f)**

Figure S51. ^{1}H NMR of compound **14f.** A broad peak for water at 3.33 ppm is observed in Figure S51 and identified by chemical shifts reported in the publication [1,2]. The peaks for 1,4-dioxane at 3.53 ppm in figure S51 and 66.35 ppm in figure S52 is noticed and identified by reference to chemical shifts described in the publication [1].

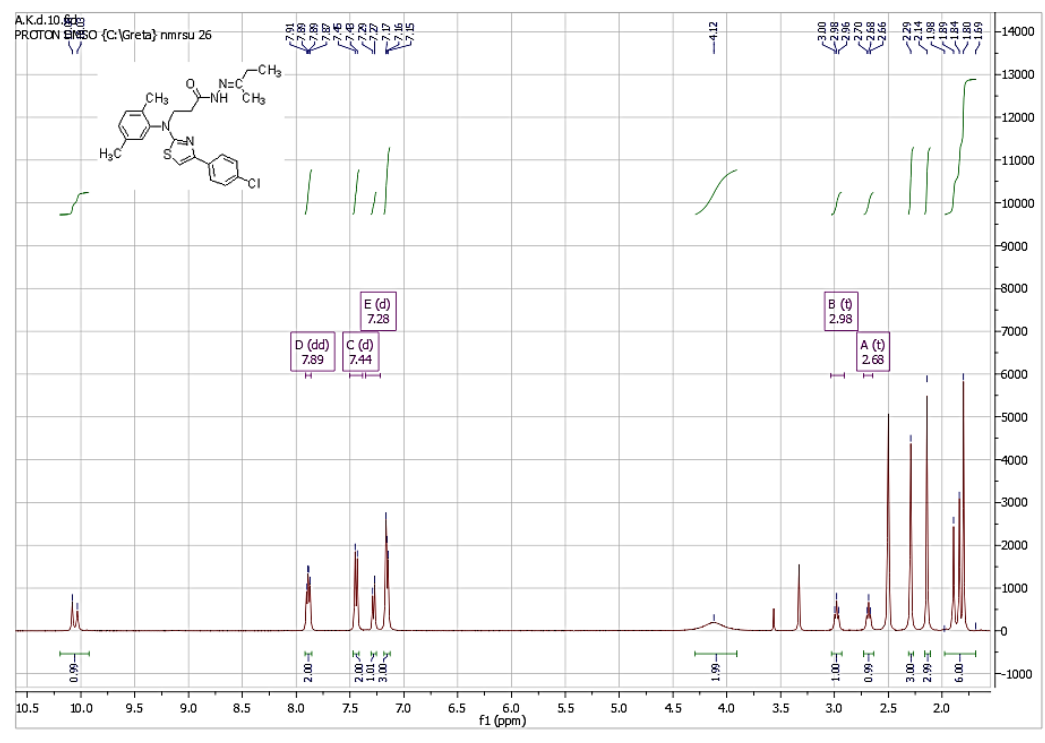

Figure S52. ^{13}C NMR of compound **14f.**

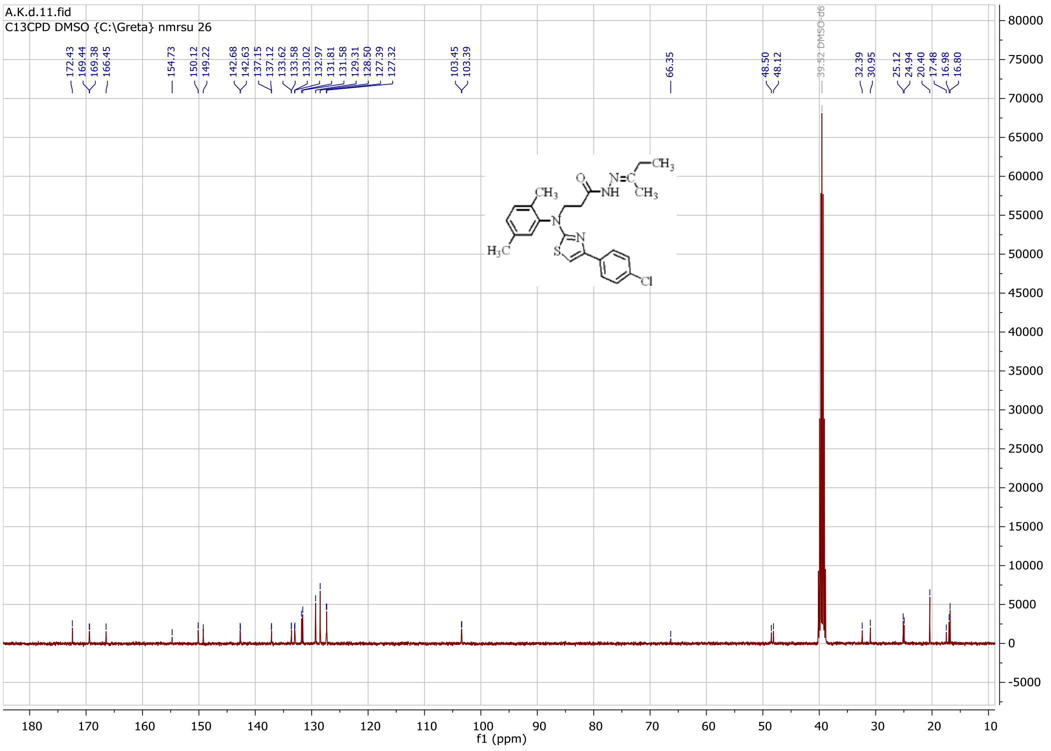

(Z/E)-3-{[4-(4-Chlorophenyl)thiazol-2-yl](2,5-dimethylphenyl)amino}-N'-(octan-2-ylidene)propanehydrazide **(15f)**

Figure S53. ^{1}H NMR of compound **15f.** A broad peak at 3.33 ppm for water is observed in Figure S53 and identified by chemical shifts reported in the publication [1,2].

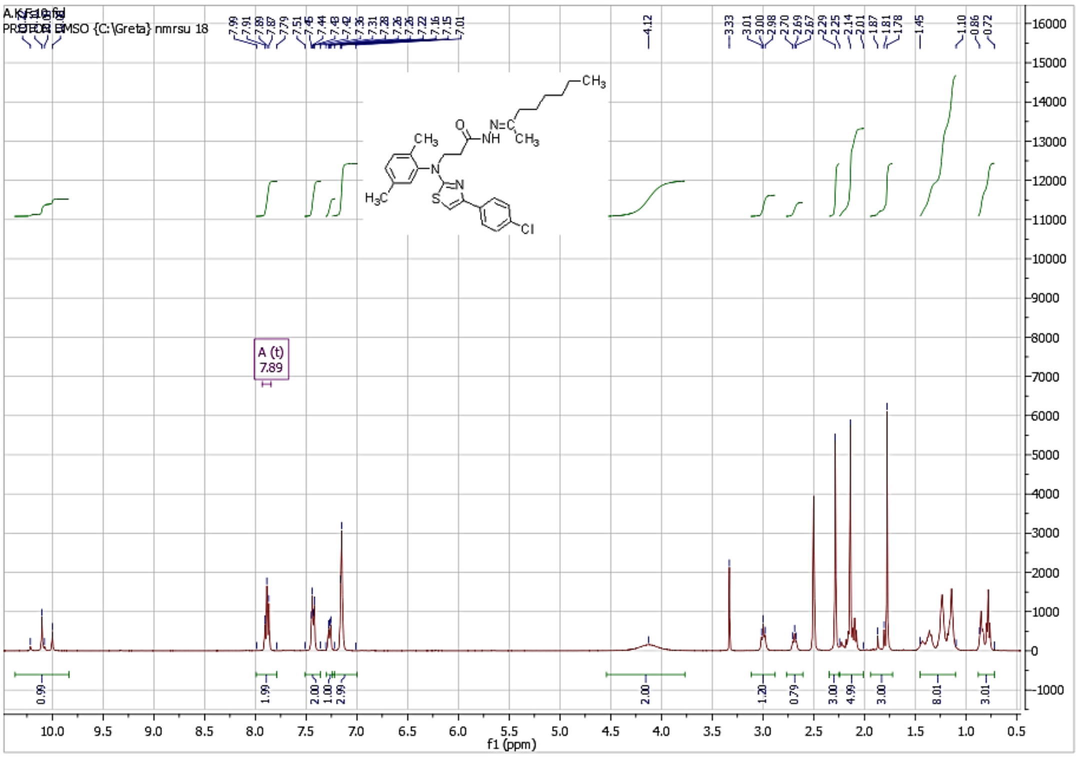

Figure S54. ^{13}C NMR of compound **15f.**

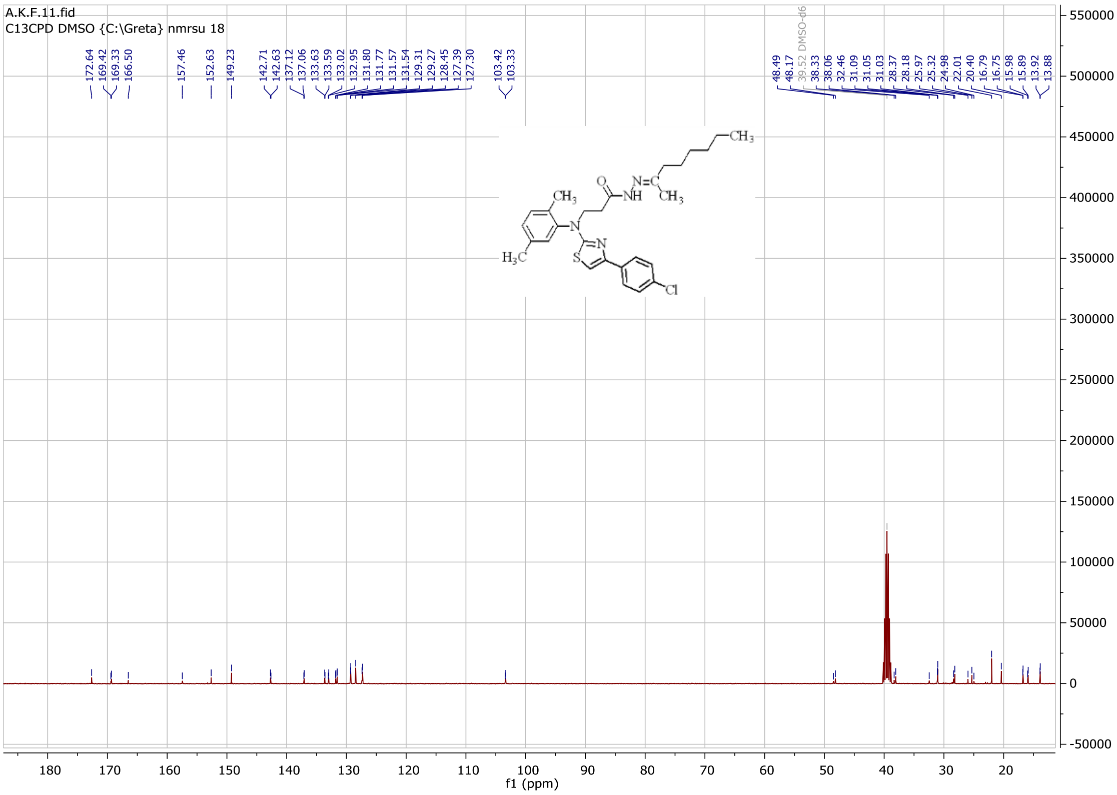

General synthesis of compounds 16c, f, h

N-[2-(1H-Benzo[d]imidazol-2-yl)ethyl]-N-(2,5-dimethylphenyl)-4-phenylthiazol-2-amine **(16)**

Figure S55. ^{1}H NMR of compound **16c.**

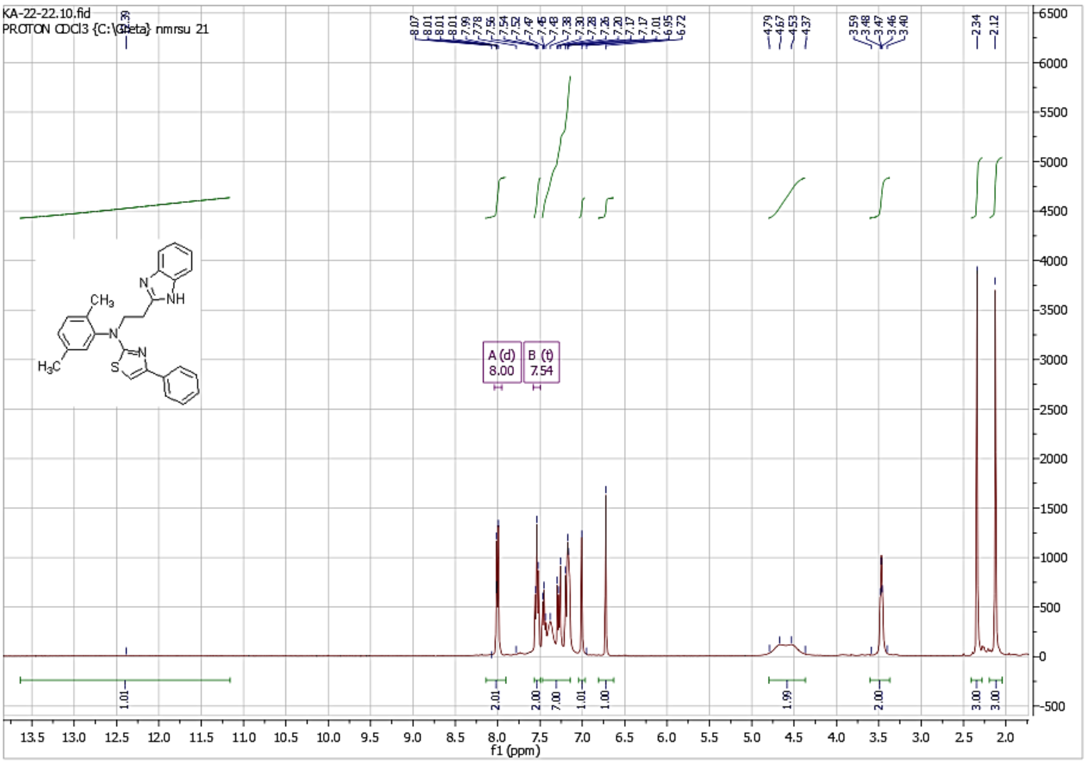

Figure S56. ^{13}C NMR of compound **16c.**

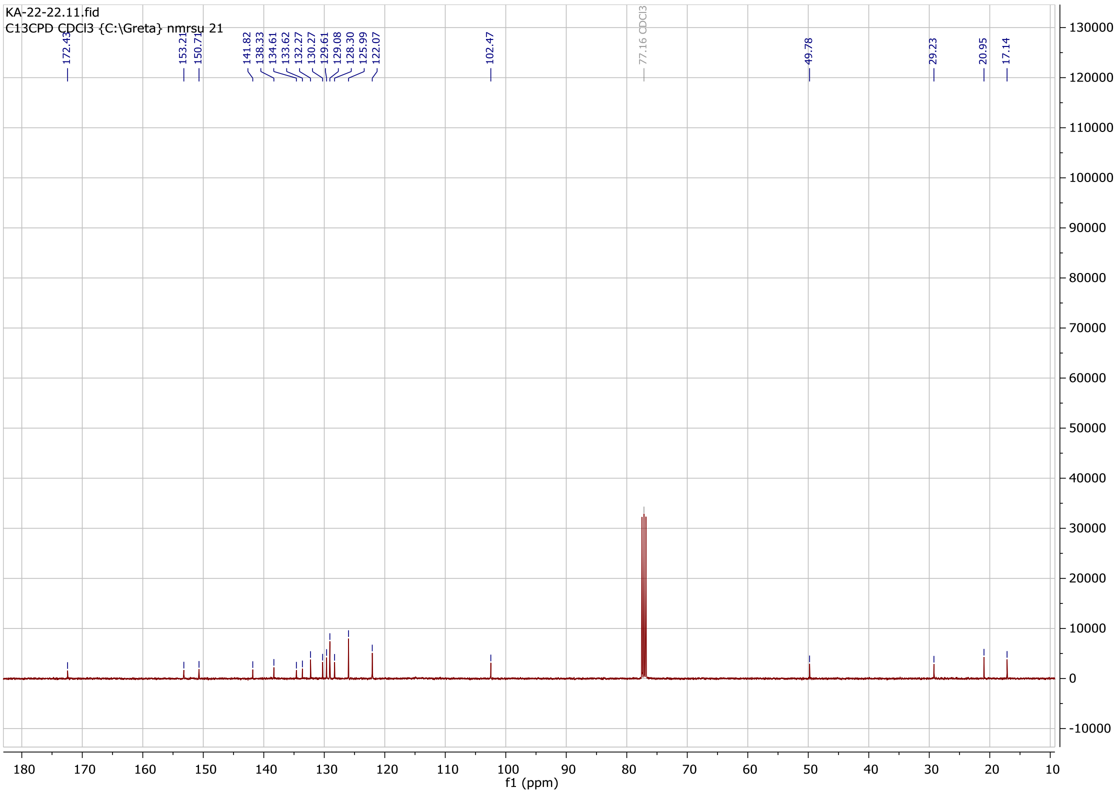

N-[2-(1H-Benzo[d]imidazol-2-yl)ethyl]-4-(4-chlorophenyl)-N-(2,5-dimethylphenyl)thiazol-2-amine **(16f)**

Figure S57. ^{1}H NMR of compound **16f.**

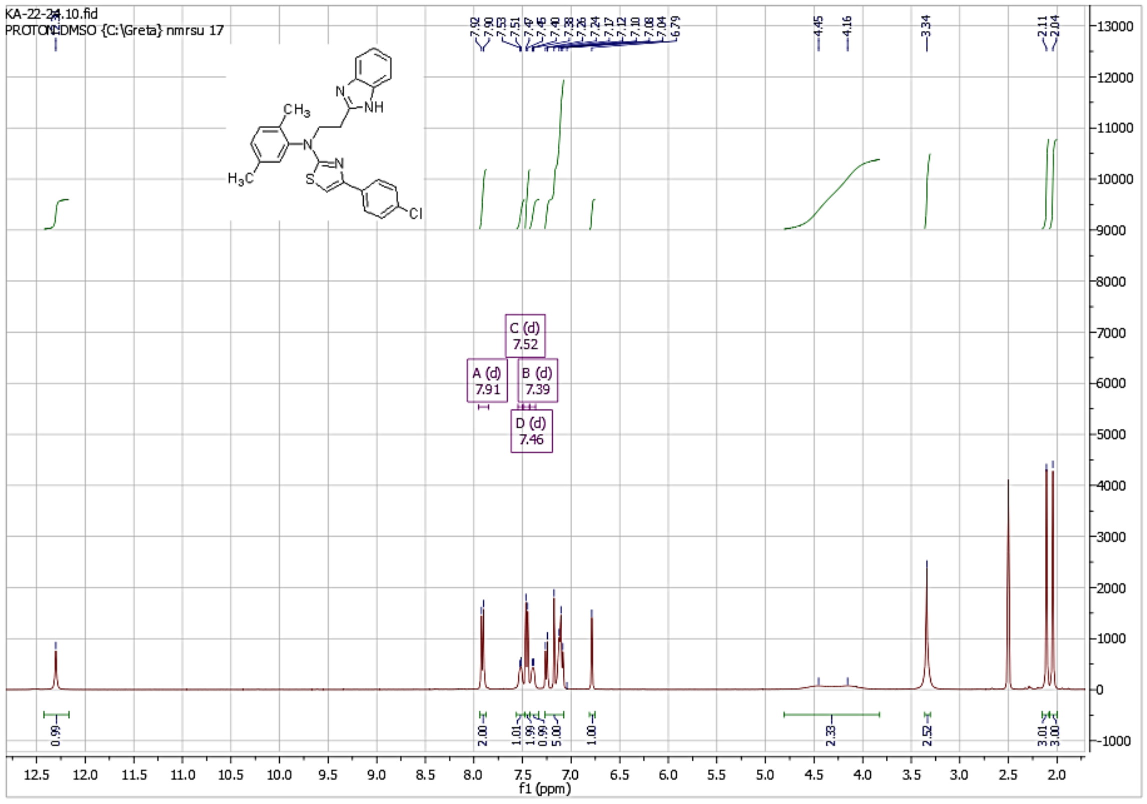

Figure S58. ^{13}C NMR of compound **16f.**

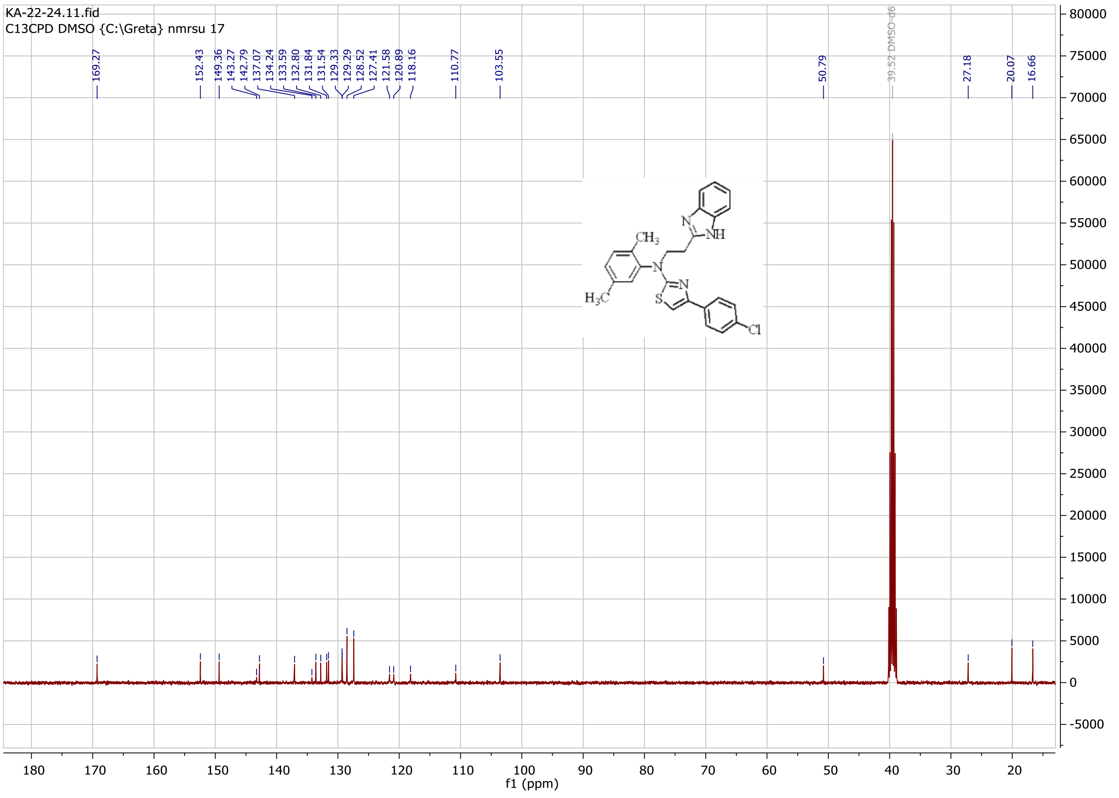

N-[2-(1H-Benzo[d]imidazol-2-yl)ethyl]-4-(3,4-dichlorophenyl)-N-(2,5-dimethylphenyl)thiazol-2-amine **(16h)**

Figure S59. ^{1}H NMR of compound **16h.**

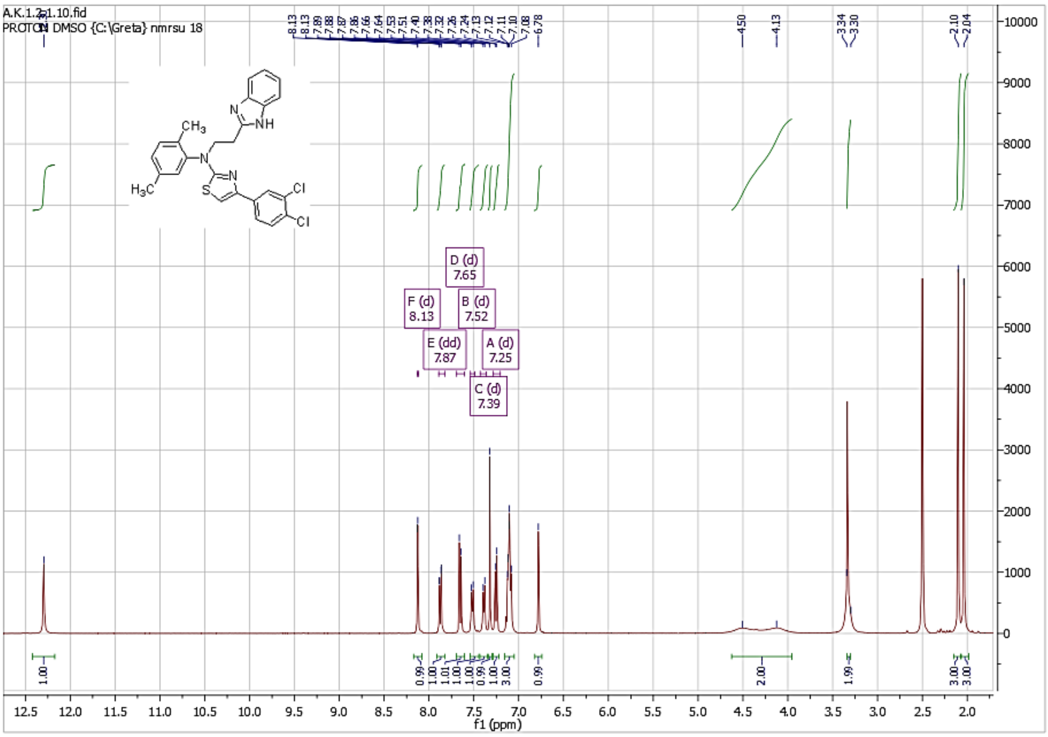

Figure S60. ^{13}C NMR of compound **16h.**

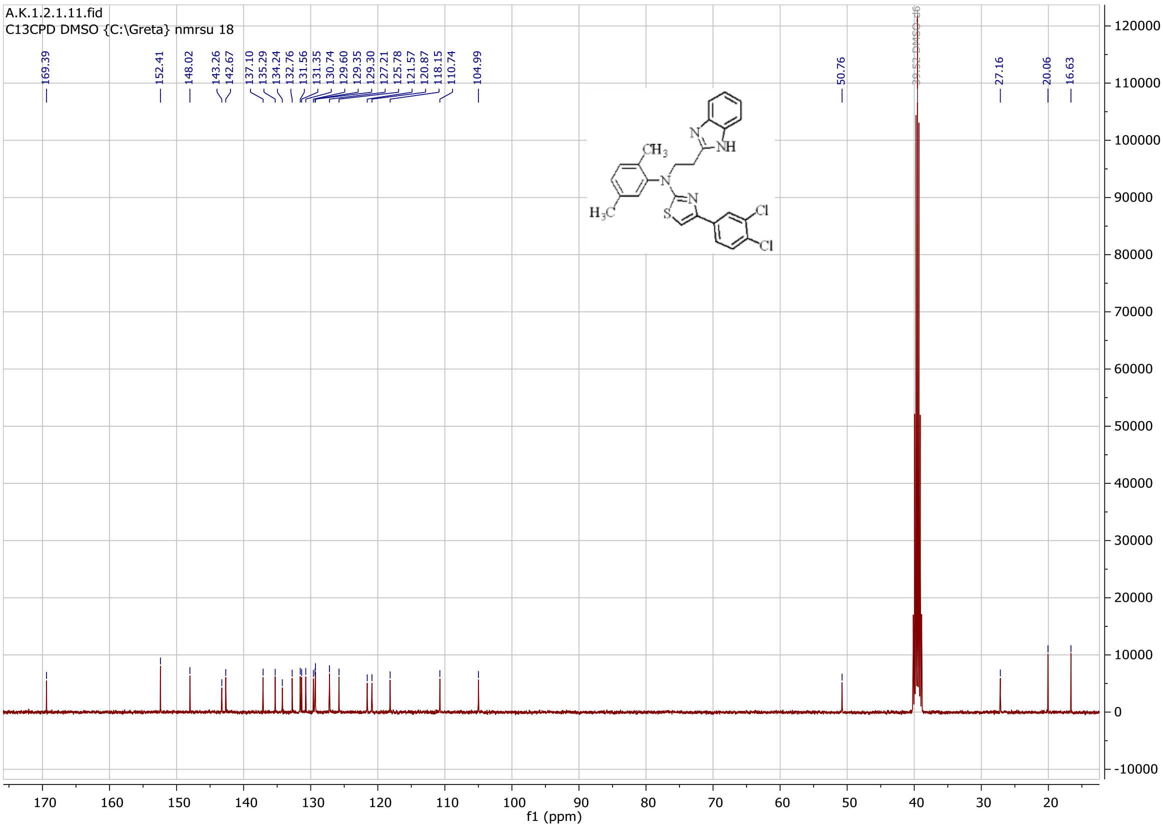

General synthesis of compounds 17c, f, h

3-[(2,5-Dimethylphenyl)(4-phenylthiazol-2-yl)amino]-N-(4-sulfamoylphenyl)propanamide **(17c)**

Figure S61. ^{1}H NMR of compound **17c.** A broad peak at 3.33 ppm for water is observed in Figure S61 and identified by chemical shifts reported in the publication [1,2].

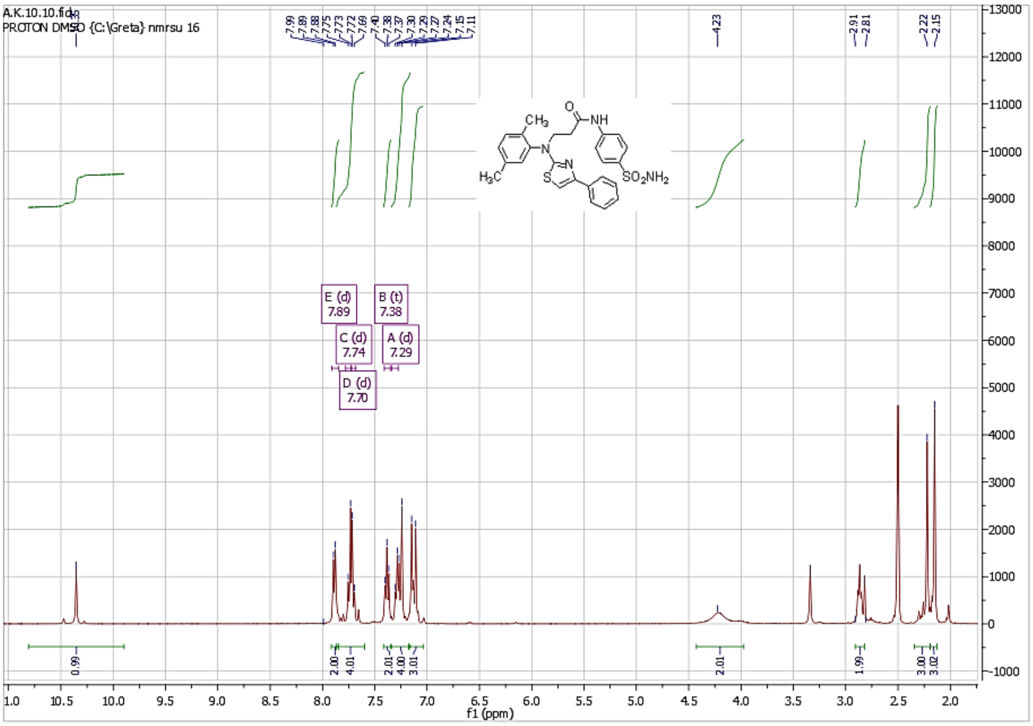

Figure S62. ^{13}C NMR of compound **17c.**

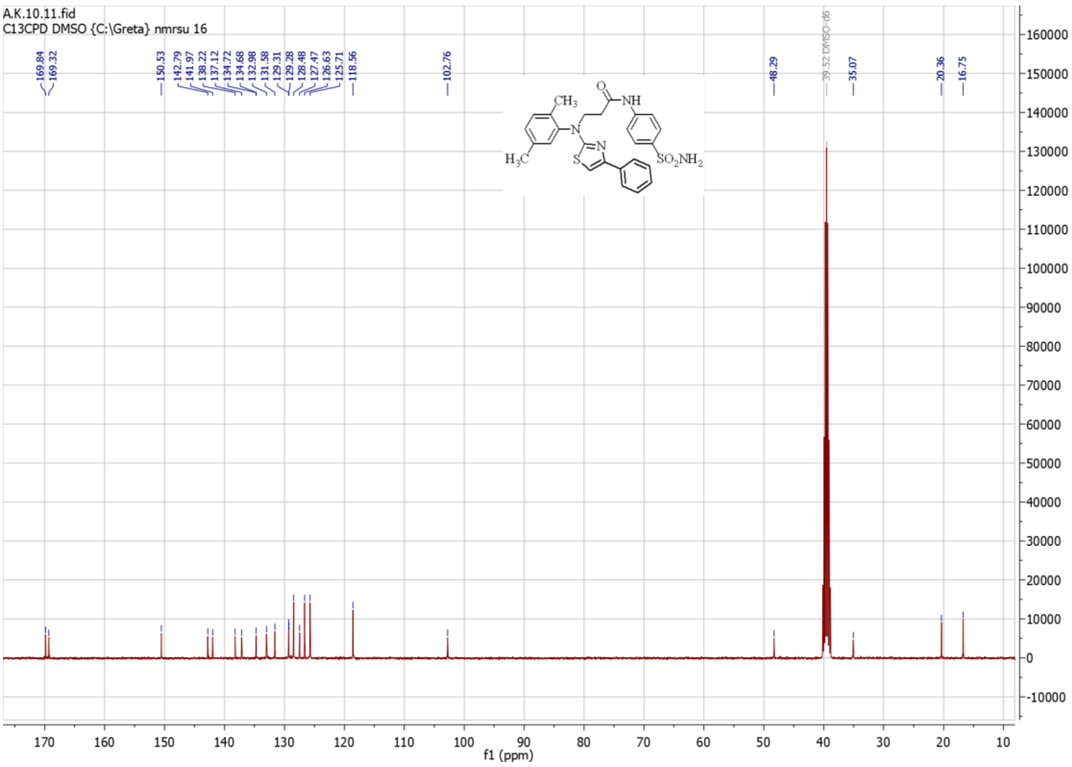

3-[(4-(4-Chlorophenyl)thiazol-2-yl)(2,5-dimethylphenyl)amino]-N-(4-sulfamoylphenyl)propanamide **(17f)**

Figure S63. ¹H NMR of compound **17f.**

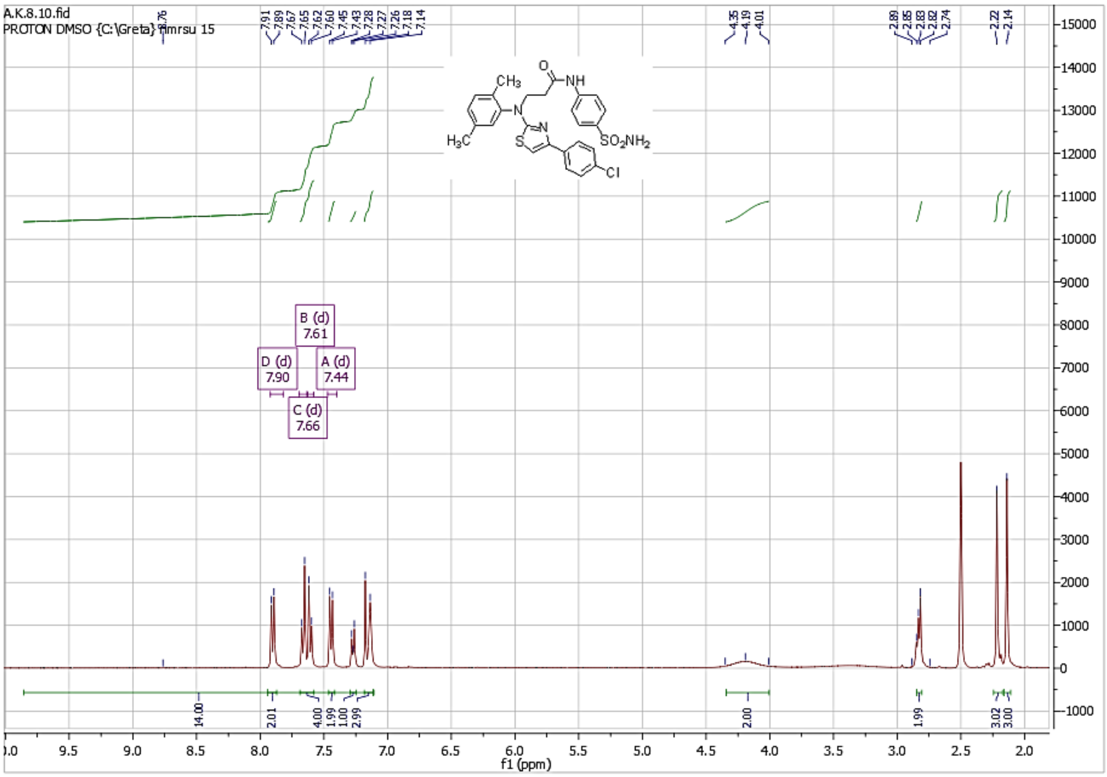

Figure S64. ¹³C NMR of compound **17f.**

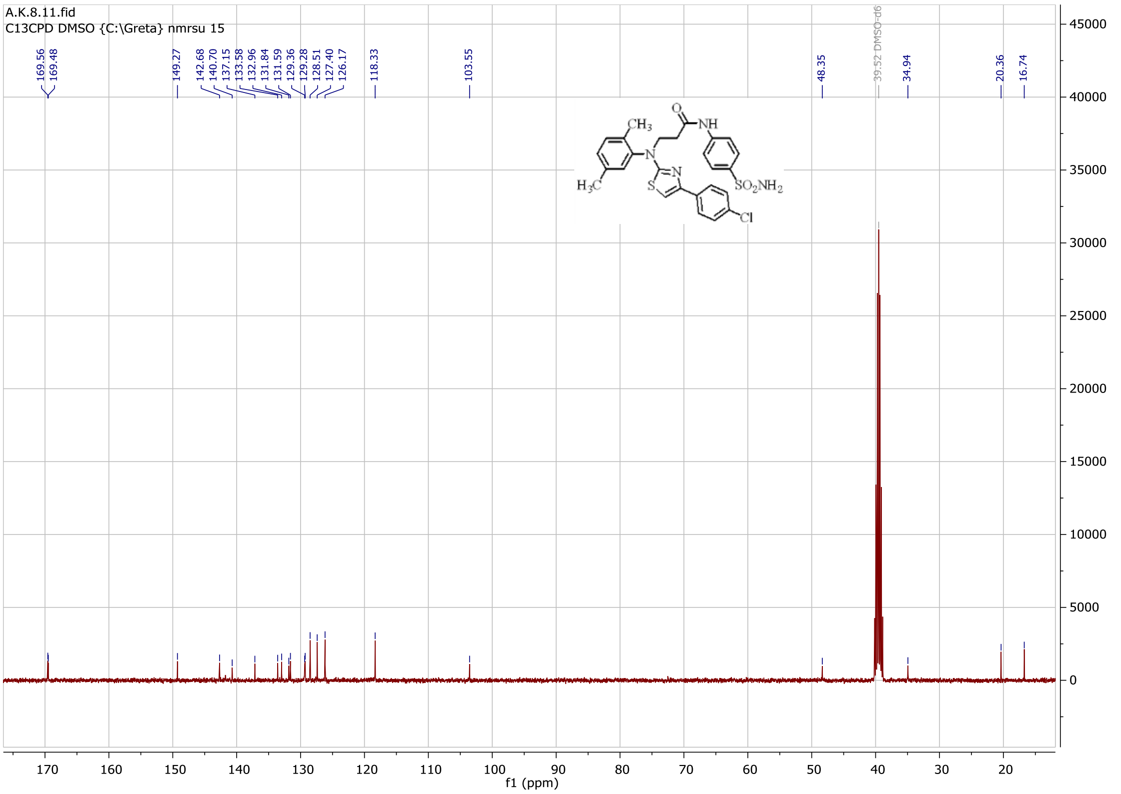

3-((4-(3,4-Dichlorophenyl)thiazol-2-yl)(2,5-dimethylphenyl)amino)-N-(4-sulfamoylphenyl)propanamide **(17h)**

Figure S65. ^{1}H NMR of compound **17h.** A broad peak at 3.33 ppm for water is observed in Figure S65 and identified by chemical shifts reported in the publication [1,2].

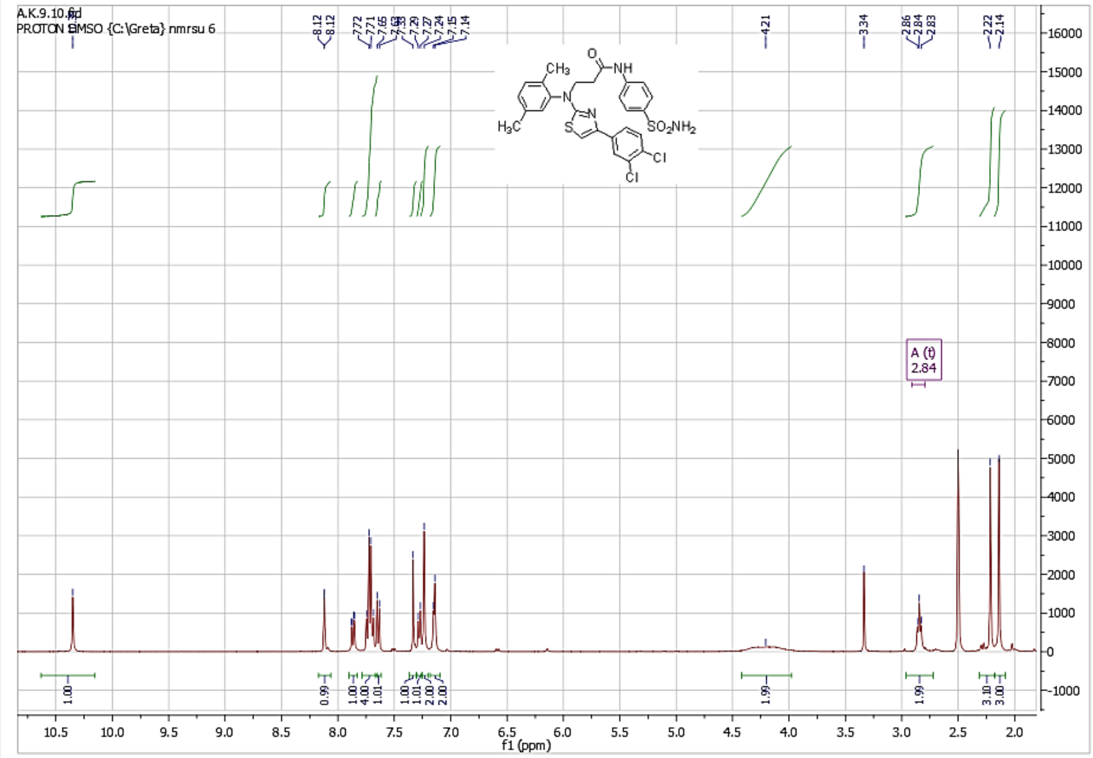

Figure S66. ^{13}C NMR of compound **17h.**

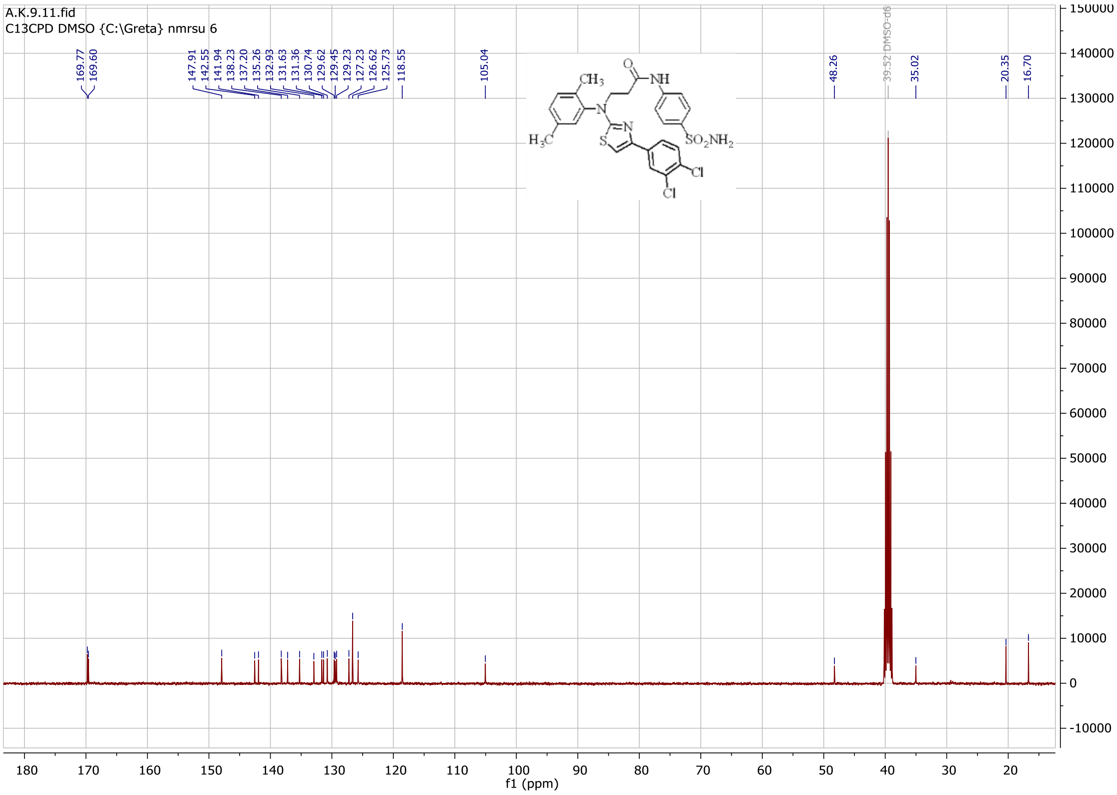

References

1. Fulmer, G. R.; Miller, A. J. M.; Sherden, N. H.; Gottlieb, H. E.; Nudelman, A.; Stoltz, B. M.; Bercaw, J. E.; Goldberg, K. I. NMR Chemical Shifts of Trace Impurities: Common Laboratory Solvents, Organics, and Gases in Deuterated Solvents Relevant to the Organometallic Chemist. *Organometallics* **2010**, *29*, 2176–2179, doi:10.1021/om100106e.
2. Babij, N. R.; McCusker, E. O.; Whiteker, G. T.; Canturk, B.; Choy, N.; Creemer, L. C.; Amicis, C. V. De; Hewlett, N. M.; Johnson, P. L.; Knobelsdorf, J. A.; et al. NMR Chemical Shifts of Trace Impurities: Industrially Preferred Solvents Used in Process and Green Chemistry. *Org. Process Res. Dev.* **2016**, *20*, 661–667, doi:10.1021/acs.oprd.5b00417.
